# Oxidative Stress Indexes for Diagnosis of Health or Disease in Humans

**DOI:** 10.1155/2019/4128152

**Published:** 2019-11-25

**Authors:** Martha A. Sánchez-Rodríguez, Víctor Manuel Mendoza-Núñez

**Affiliations:** Unidad de Investigación en Gerontología, Facultad de Estudios Superiores Zaragoza, Universidad Nacional Autónoma de México, Ciudad de México, México. Av. Guelatao No. 66, Col. Ejército de Oriente, Iztapalapa, Ciudad de Mexico, CP 09230, Mexico

## Abstract

Oxidative stress (OS) is the imbalance between oxidant and antioxidant molecules, in favor of oxidants, that causes aging and disease. Many studies have been published that demonstrate the relationship between OS and human health and disease; however, the following questions arise: (i) how are we sure that the OS is present in a biological process? (ii) Is the OS reported in the different investigations equivalent? (iii) What are the best oxidant and antioxidant markers for OS diagnosis? (iv) Can we establish the types and the intensity of the OS? (v) Does OS index could be useful for research and/or application in clinical medicine? In this regard, several indexes have been proposed to measure OS in humans relative to the state of health and disease, among which the following can be highlighted: *Oxidative Stress Index* (OSI), *Tiol Ratios* (-SH/TT, -SS/-SH, and-SS/TT), *Glutathione Ratio* (GSSG/GSH), *Oxidative Stress Score* (OSS), and *OXY-index*. Therefore, the aim of this review is to present the state of the art of knowledge about OS indexes for diagnosis of health or disease in humans. We searched for articles in English or Spanish in the PubMed/MEDLINE and Scopus electronic databases published up until May 2019. The keywords used were “oxidative stress,” “index,” and “oxidative stress index.” It was identified 11479 records in both databases, and 490 articles were analyzed. Our review suggests that all indexes analyzed allow diagnose and differentiate the OS related to human health and disease. Also, the studies on OSI, Oxy-score, and OSS indexes have proven to be reliable, practical, and with clinical utility. However, it is necessary to continue with longitudinal studies, especially assess the usefulness of the indexes in the clinical prognosis, and make comparative studies between the different indexes.

## 1. Introduction

Since that Gerschman et al. and Harman in the 50s proposed the Free Radicals Theory to explain the disease and aging, respectively [1, 2], this theoretical orientation has had a great impulse, mainly after the description of superoxide dismutase (SOD), an antioxidant enzyme, in 1969 [3]. During these years, many researchers have sought the relation among different pathological events and the aging, with the free radicals (FR), oxidized molecules, and antioxidants, based on Free Radicals Theory.

The term “oxidative stress” (OS) arises in 1985, as a proposal of Helmut Sies that expresses the imbalance between oxidant and antioxidant molecules, in favor of oxidants, that causes aging and disease [4]. This concept has revolutionized the field of knowledge of FR; thus, most of the research about the oxidants and antioxidants is referred as OS. Likewise, the studies of OS mechanisms had described that the reactions involved in this process are oxidation-reduction reactions (redox) [4, 5].

In parallel, the knowledge about the homeostatic function of the FR, oxidants, and antioxidants has been emerging, highlighting its importance in the signaling and maintenance of cellular mechanisms, because FR and oxidants (called reactive species) are essential components in several processes such as phagocytosis and maintenance of the cell membrane, and physiological function such as immunological mechanisms and vascular function, among others [6, 7]. In this sense, actually it is recognized that OS may be separated in oxidative eustress and oxidative distress. In the oxidative eustress, the physiological mechanisms of the reactive species (RS) intervene, mainly by H_2_O_2_ reactions, and in the oxidative distress, there are the oxidative damage to biomolecules with disrupted redox signaling that may be causing disease and aging [4, 8]. Although these terms are little used, most researchers use the term OS when they refer at the imbalance between oxidant and antioxidant components.

The central problem for the application of OS in biology and clinical medicine is its measurement, considering the complexity of the process and the number of components oxidants and antioxidants that make it up. In this sense, the OS has been measured using different markers, both biomolecules oxidized (lipids, proteins, and DNA) and antioxidants (enzymes and nonenzymatic antioxidants), besides of oxidants (reactive oxygen species), everything in biological samples (Table 1) [4, 9–12].

Another problem is the variety of methods used to measure the markers in biological samples due to the difference among them. Those methods have different sensibility and reliability and advantages and disadvantages, which hindering its interpretation, and some require very sophisticated techniques [9–11, 13], which have been widely discussed by Forman et al. and Marrocco et al. [9, 13].

On the other hand, the measurements of OS for diagnosis of health or disease in humans must be with noninvasive procedures, so that the employment of blood, urine, or other biological fluids is the commonest.

In this context, the following questions arise: (i) who are we sure that the OS is present in a biological process? (ii) Is the OS reported in the different investigations equivalent? (iii) What are the best oxidant and antioxidant markers for OS diagnosis? (iv) Can we establish the types and the intensity of the OS? (v) Does OS index could be useful for research and/or application in clinical medicine?

Thus, starting from the theory of OS proposed by Sies et al. [4], if exposure to exogenous or endogenous oxidants increases or is insufficiently balanced by antioxidants, oxidative damage occurs to biomolecules, which gives the option to select different oxidants, oxidized molecules, and antioxidants to be measured in a research, making the comparability of the results difficult and generating confusion, perhaps because with individual markers, it is not considered that OS is an integral and dynamic process.

With this view, several proposals have been published to evaluate the oxidant/oxidized components and the antioxidants integrally considering a global index to avoid the bias of each marker measurement, which are intended to demonstrate their ability to differentiate the health of the disease, as well the changes produced by the aging process, although the clinical value of the different proposals has not been systematized and/or analyzed.

Therefore, the aim of this review of the literature is to present the state of the art of knowledge about OS indexes for diagnosis of health or disease in humans.

## 2. Material and Methods

### 2.1. Data Sources and Search Strategy

We searched for articles in English or Spanish in the PubMed/MEDLINE and Scopus electronic databases published up until May 2019, which included the measurement of oxidative stress using oxidative/antioxidant ratios or indexes. The keywords used were “oxidative stress,” “index,” and “oxidative stress index,” and the specific name of the index was found such as “OSI,” “glutathione ratio,” “thiol ratio,” “oxidative index,” “oxidative balance score,” “OBS,” “OXY score,” and “oxidative stress score.” References in each article were searched to identify missed studies.

### 2.2. Eligibility Criteria and Study Selection

We included the studies that involved human subjects, with experimental or observational design (cross-sectional or longitudinal), assessed any disease or health state as primary event, and used biological samples, no tissues, any age, and sex. We excluded animal and cell culture studies, conference abstracts without full text, case reports and case series, reviews of any kind, editorials, and opinion articles. Also, we excluded the studies that measured oxidants and antioxidants, but the authors did not relate them, that is, they did not calculate a sum or ratio between them.

### 2.3. Analysis of the Information

After removing the duplicate records, we screened the relevant studies from the references retrieved from the databases, reviewing the titles and abstracts. We obtained the full texts of the relevant studies to assess for inclusion or exclusion in this study.

The author name, publication year, study design, country, population included (age group, sex), sample size (subjects included in the study), biological sample, main outcome, results, and conclusion related to the ratio or index were extracted from each included study.

We found a great number of studies that use the oxidative stress index (OSI) and thiol ratios, so we analyzed this information in a different way to the rest of the ratios and indexes; in fact, the reference list of those indexes is in supplement files ([Supplementary-material supplementary-material-1]).

The topics included in this review are (i) oxidative balance, (ii) glutathione ratio, (iii) thiol ratios, (iv) oxidative stress index, (v) oxidative index, (vi) oxidative stress profile, (vii) OXY score, and (viii) oxidative stress score.

## 3. Results and Discussion

**Figure 1:**
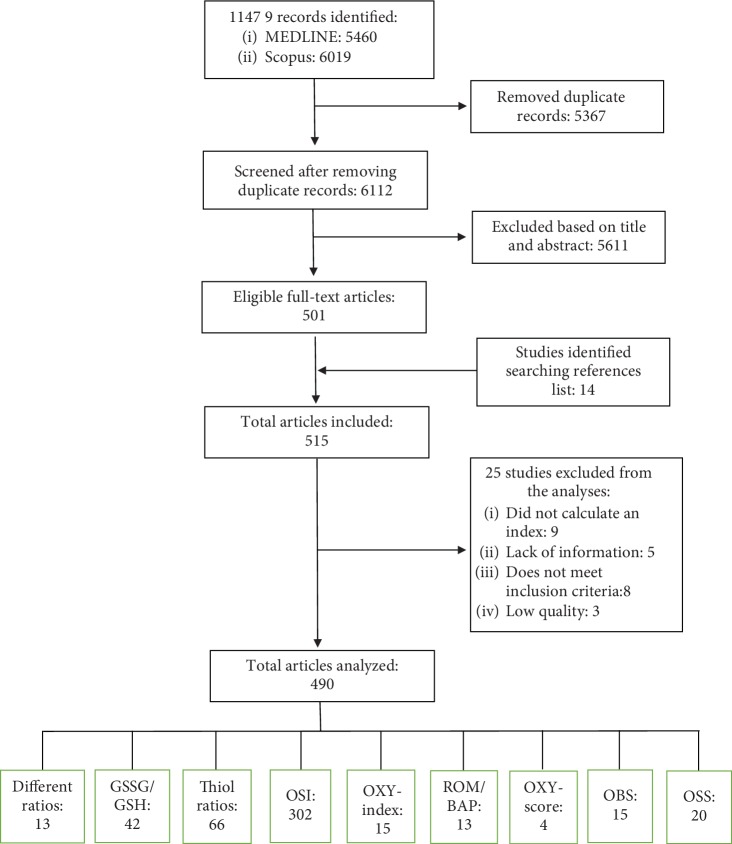
Diagram of study selection for the review.

It was identified 11479 records, in both databases, with the selected keywords. After removing the duplicate records and that did not meet the inclusion criteria, 501 full-text articles plus 14 records identified searching on articles references were eligible. After reviewing the full-text articles, 25 were excluded by different reasons. A total of 490 articles were analyzed (Figure 1).

The information is ordered according to the name of the index, trying to maintain a chronological order and with an increase in the complexity of the calculation, except the first section because we included a series of different calculations that have in common the use of a ratio and they do not have a specific name.

### 3.1. Oxidative Balance

One simple form to measure the oxidative/antioxidant balance is calculating a ratio. The first proposal to use a ratio to measure oxidative balance was the oxidized/reduced glutathione (GSSG/GSH). Initial studies in animal models indicated that its increase is an index of OS *in vivo* and suggests that it can be used as a complement of other parameters [14]. Thus, this ratio has been used in many studies in humans over the past 10 years, so we analyzed its importance in a separate section.

The first reports of an attempt to find the relationship between the oxidant and antioxidant counterparts of a biological sample, and not the evaluation of individual markers, are two studies conducted on seminal samples. In these studies, reactive oxygen species (ROS) and total antioxidant capacity were measured, both by chemiluminescence, and a score called ROS-TAC score was calculated. The computation of the ROS-TAC score is complex, with mathematical transformations, standardizations, and a principal component analysis as part of the computation [15, 16], which makes it an impractical score; thus, there is neither study with it.

To avoid complex calculations that are not easy to apply to the clinical setting, the researchers resorted to obtaining ratios between an oxidized molecule and an antioxidant. In this sense, the most used oxidation markers have been lipoperoxide level, measured as malondialdehyde (MDA or TBARS) or total lipid hydroperoxides (LOOH), but for the measurement of the antioxidants, a variety of parameters have been used, such as antioxidant enzymes (superoxide dismutase [SOD] or glutathione peroxidase [GPx]) and different ways to measure the nonenzymatic antioxidant capacity (total antioxidant capacity [TAC], ferric reducing antioxidant potential [FRAP], total radical-trapping antioxidant parameter [TRAP], oxygen radical antioxidant [ORAC]) [17–24]. The most commonly used biological samples are the components of the blood, although these reasons have also been applied in measurements obtained from saliva and follicular fluid (Table 2).

Regarding the name used to refer the ratio, some authors called it as “oxidative stress index” (OSI) [22, 23], although this term is most used for other relation that we discuss later. Becatti et al. called their ratio as “redox index,” and they define it as a reliable estimation of redox status in humans [24], but neither is it a good option because they measure the ORAC/MDA ratio which is not necessarily a measure of redox status. Therefore, we think it is better to express only the calculated ratio to refer it.

The ratio can be calculated from the oxidized molecule to antioxidant, since the shift of the balance toward the oxidative side is considered to represent OS. The lipids oxidized, measured as MDA, or proteins oxidized, measured as advanced oxidation protein products (AOPP), are the main used oxidized molecules, related to total antioxidant status (TAS) or TRAP as antioxidant measure; thus, the MDA/TAS and AOPP/TRAP ratios are recommended indicators of the OS [17, 21]. The use of the ratio toward the antioxidant side indicates only the antioxidant-oxidant imbalance [20, 24].

Another way to get the oxidizing/antioxidant ratio is to measure the oxidants like superoxide anion (O_2_^−·^) and nitric oxide (NO), and glutathione (GSH) or melatonin as antioxidants, although their use should not be frequent, because it exists only one report about melatonin in which it was calculated the NO/melatonin and MDA/melatonin ratios; the authors suggest that these ratios reflect an oxidant-antioxidant balance and could be used to determine OS homeostasis [25], but we should consider that within the OS homeostasis, melatonin is not one of the essential markers. On the other hand, if we calculate the ratio with the oxidants (O_2_^−·^, NO_2_^−^) and GSH as the antioxidant, a problem may arise because the measurement of the oxidants is not easy to apply at patient samples; thus, there remains doubt about its possible usefulness [26].

### 3.2. Glutathione Ratio

As we noted above, the GSSG/GSH ratio is the first attempt to assess the OS as a dynamic way, since this coupler represents the oxidant/antioxidant homeostasis. After a series of animal studies, Curello et al. in 1987 improved the assay of total and oxidized glutathione in blood samples, which has been useful in monitoring of clinical status [27]. From this, different procedures have been developed with various technologies to carry out the measurement not only in blood but also in other biological samples and tissues. In this sense, pitfalls in the measurement of both reduced and oxidized glutathione with those technologies have recently been discussed, pointed an alert in the interpretation of the results [28]; however, glutathione ratio is an indicator of OS widely used.

The ratio can be analyzed from GSH to GSSG or inversely GSSG to GSH; in either direction, it is considered as a marker of intracellular OS. If the ratio is obtained from reduced to oxidized glutathione, low values indicate OS; in the opposite direction, a high value denotes OS. Many studies have been realized using the ratio in both ways; thus, we revised them by separation. It is important to keep in mind that most of the time, the glutathione ratio is used as another marker of the OS, as was suggested from the beginning of its use [14], not as an individual indicator, like the ratios pointed above.

Furthermore, it is possible to express the glutathione ratio as redox potential (*E*_h_ GSH/GSSG), which is a way to represent the redox environment in biological fluids [29]. The redox potential is calculated by the Nernst equation as follows [30]:

(1) $$\begin{array}{c} E_{\text{h}}\left(\text{mV}\right)=E_{0}+\frac{RT}{2F\;\ln\left(\text{GSSG}/{\left(\text{GSH}\right)}^{2}\right)}, \end{array}$$

where *E*_0_ = −264 mV (GSH/GSSG couple standard potential for pH 7.4), *R* is the gas constant (8.314 J/°Kmol), *T* is the absolute temperature (°K), *F* is the Faraday constant (9.6485 × 10^4^ C/mol), and GSH and GSSG are the concentrations in moles/liter. In the interpretation, higher *E*_h_ values indicate a greater potential for oxidation or OS.

The first studies in humans that use this ratio have begun in the 90s. In this review, we found three reports in this decade. Two who obtained the GSSG/GSH ratio of measurements made in the blood of newborns and whose results suggest its use as a noninvasive method to evaluate the OS [31, 32]. The other research is an experimental study with free fatty acid (FFA) infusions in which the lipoperoxide level was measured through the MDA and LOOH, in addition to the GSH/GSSG ratio. In this study, the role of the ratio in the effect sought is not directly concluded, but an inference is made when it considers that the OS intensifies with the increase of FFA [33].

Subsequently, in this century, an avalanche of information has emerged. Twenty-four articles located inform the use of the GSH/GSSG ratio to evaluate the antioxidant/oxidant balance, which is on the Table 3 [33–55]. The sample mainly used is whole blood and its derivatives (erythrocytes, polymorphonuclear cells, or plasma), and other markers are added, such as oxidation products, oxidants, and enzymatic and nonenzymatic antioxidants, to obtain a more comprehensive measurement of the OS, although it is possible to use exclusively the glutathione ratio, but remember that this ratio measures only the intracellular OS [35, 36, 49, 51, 55]. Regarding to the GSSG/GSH ratio, fifteen studies were localized, and blood is the biological sample used mainly, but also it is possible to measure glutathione in saliva [56, 57]. As in the previous ratio, it is common to add more OS markers [30, 31, 56–68], and the measurement of the ratio from oxidized to reduced form is a good marker of oxidant/antioxidant imbalance in different clinical situations (Table 4).

The redox potential (*E*_h_) is a complement of the GSH/GSSG ratio to evaluate the redox status [30, 34, 37, 43, 49, 51, 55], but it can be used as an independent measurement since it provides indirect information about the redox state of all cell types, although the measurement has been performed only in erythrocytes, because the cells have similar essential functions [69].

Nevertheless, the glutathione ratio seems to be a promise OS marker, it is limited because it only measures intracellular environment without considering other OS components as antioxidant enzymes and oxidized biomolecules.

### 3.3. Thiol Ratios

The thiols are compounds with high vulnerability to the oxidation by reactive species, mainly of oxygen, due to their -SH group, which is oxidized to their disulfide (-SS-). As this is an oxidation reaction, the imbalance between both forms in favor of the oxidized molecule is considered as OS, being a dynamic homeostasis involved in many diseases and alterations of the physiological state [70, 71]. Many proteins, nonproteins, and low molecular mass thiols have -SH group, but only cysteine and glutathione are considerate in the redox balance, where cysteine represents the extracellular environment and glutathione the intracellular. Then, the term “thiol ratio” is used to refer the measurement of these aminothiols together, both in its oxidized and reduced forms. There are some techniques to their measurement, less complex and automated what has facilitated its use in different investigations.

After the proposal of an automated procedure to evaluate native thiol (-SH), total thiol (TT), and thiol-disulfide (-SS) [70], -SH/TT, -SS/-SH, and -SS/TT ratios have been calculated; where if the value of -SH/TT ratio is low and both ratios of -SS are high, the OS is present. Also, it is possible to present the result as a percentage, multiplying the total obtained by 100.

Its use has spread widely to become the second most popular indicator of OS so far. In this review, we found sixty-six studies that refer them. All the studies, except one [72], were carried out in Turkey in a variety of age groups and clinical situations since 2015. The thiols can be measured in serum/plasma, but semen sample [72] and aqueous humor [73] also have been used, and they are applicable to evaluate the oxidative state in a variety of clinical events, from alterations during pregnancy, cardiovascular, psychiatric, neurological, hereditary, infectious diseases, etc. Although the information is abundant, the analysis result has not shown substantial differences among the studies, because all agree in recommending the thiol ratios to their use in clinical events; therefore, we only did a descriptive analysis of the information (Table 5) and presented references found in a supplementary file in case of any reader is interested in consulting the information.

It is important to note that, contrary to glutathione ratio, the thiol ratios generally do not need other markers to interpret the OS, although antioxidant enzymes, FRAP, TAC, total oxidant capacity (TOC), and ischemia-modified albumin (IMA), have been some of the other markers included to complement the interpretation.

The thiol ratios are the OS markers used more recently, but like the glutathione ratio, to make a complete interpretation of the OS, it is necessary to include antioxidant enzymes and oxidized biomolecules.

### 3.4. Oxidative Stress Index (OSI)

All the previous proposals are recognizing that OS is a dynamic process, because they consider both the oxidizing and antioxidant components in a single calculation; however, only one type of oxidant and antioxidant is related, which is a disadvantage because it is necessary to evaluate many components to establish more effective if the OS is present. Hence, other proposals have emerged that consider the measurement of oxidant and antioxidant activity whose effect is produced by the action of several components of OS measured as one.

Those are based in fact that the measurement of different oxidants and antioxidant molecules separately is not practical because they are costly and time-consuming, and frequently, the procedures are complex; moreover, their oxidant or antioxidant effects are additive [74, 75]; therefore, it is possible to measure the total oxidant or antioxidant capacities of a biological sample and related them in a ratio, called index.

The antioxidant capacity is measured by different procedures identified by their acronyms:

- Ferric reducing antioxidant potential (FRAP) or total antioxidant potential (TAOP): measurement of the ability of the sample to reduce Fe^3+^ to Fe^2+^ as a result of antioxidant activity. The formation of a complex between Fe^2+^ and 2,4,6-tripyridyl-1,3,5-triazine (TPTZ) is measured [20, 76]
- Oxygen radical absorbance capacity (ORAC): inhibition measurement of the peroxyl radical-induced oxidation initiated by thermal decomposition of 2,20-azobis(2-amidinopropane) dihydrochloride (AAPH) [24]
- Total antioxidant activity (TAA): it is the assessment of the ability of a sample to inhibit linolenic acid peroxidation. The results are expressed as percentage of inhibition of linolenic acid peroxidation produced by samples [58]
- Total antioxidant capacity (TAC): the method is based on the hydroxyl radical (OH^·^) produced via the Fenton reaction using a mixing ferrous ion solution and H_2_O_2_. The OH^·^ generated reacts with o-dianisidine molecule to form dianisidyl radicals [75]
- Total antioxidant status (TAS): the method is based on the production of the radical cation (ABTS^·+^) by the incubation of ABTS (2,20-azino-bis[3-ethylbenzothiazoline-6-sulphonic acid]) with peroxidase (metmyoglobin) and H_2_O_2_ [17]
- Total radical-trapping antioxidant parameter (TRAP): measurement of hydrosoluble and/or liposoluble plasma antioxidants using chemiluminescence inhibition time induced by 2,2-azobis(2-amidinopropane) [21]

Except in TAA, the results in all the quoted methods are expressed as Trolox equivalents (*μ*mol/L), which is the antioxidant standard.

Likewise, the measurement of the oxidant capacity can be carried out by different methods:

- Total oxidative capacity (TOC): assay based on the oxidation of 3,5,3′,5′-tetramethylbenzidine (TMB) to colored radical cations in the presence of peroxides by peroxidase. It used to determine total peroxides in *μ*mol/L [77, 78]
- Total oxidant status (TOS): it is the measurement of the oxidation of Fe^2+^ to Fe^3+^ by peroxides of the sample to produce a Fe^3+^-xylenol orange complex. This assay quantifies total peroxides; the results are expressed as H_2_O_2_*μ*mol Eq/L [75]

Some indexes are in the literature, but the most used is called “oxidative stress index” (OSI) initially proposed in 2003 [18]. This index has had few modifications across the time, but all the studies agree in that it is a ratio between TOS and TAS expressed in arbitrary units:

(2) $$\begin{array}{c} \text{OSI }\left(\text{AU}\right)=\frac{\text{TOS }}{\text{TAS}}, \end{array}$$

where OSI is in arbitrary units (AU), TOS is in *μ*mol H_2_O_2_ Eq/L, and TAS is in *μ*mol Trolox Eq/L. Sometimes, the result is multiplied by 100 to represent the percentage ratio.

TAC or TAOP can also be used as antioxidant component because being in the same units (*μ*mol Trolox Eq/L), and TOS may be replaced by TOC.

As we pointed above, OSI is the index most frequently reported in the literature. In this review, we found 302 studies in humans since 2003 that use this index, the majority carried out in Turkey, mainly in adult events such as cardiovascular diseases, alterations in the pregnancy, and psychiatric disorders. As the thiol ratio information, after analyzing the studies, we observe that most research recommends the use of OSI without other important contributions to this review; thus, we present only a descriptive analysis and the references in the supplementary file. However, we want to note that the measurement of oxidant and antioxidant activities for the calculation of the index has been carried out on biological samples other than blood, such as semen [79–81], urine [82, 83], cerebrospinal fluid [84], amniotic fluid [85], vaginal washing fluid [86], and gingival crevicular fluid [87–89], in addition to the saliva [90–95] and aqueous humor [96, 97] that have also been used in the measurement of thiols (Table 6).

Like the other calculations, OSI also has been accompanied of several OS markers such as MDA, DNA damage, hydroperoxides, protein carbonyls, IMA, thiols, antioxidant enzymes, prolidase, paraoxonase, and antioxidant vitamins, many times in order to show the relationship between the different markers and prove their usefulness as an independent indicator of OS. All this information makes this index a very promising way to measure the OS; however, there are still doubts regarding its interpretation. Actually, for its interpretation, the increase in the value of the index indicates a greater intensity of OS, but it has not been clearly established which is the cut-off value for the OS, necessary value to better clinical application, because in clinical practice, it is important to be able to differentiate oxidative eustress and oxidative distress, as we have pointed early in this review.

### 3.5. Oxidative INDEX

This is a proposal of Vassalle et al. (2008) that also related the serum oxidative/antioxidative capacities; only that for this index, the oxidative part is the measurement of total hydroperoxides by dROMs and to the serum antioxidant activity has used a procedure called OXY Adsorbent Test [98]; both methods are automated and commercial.

The dROMs or derivatives of Reactive Oxygen Metabolites, also called diacron ROM, are based on the measurement of sample alkoxyl (RO^·^) and peroxyl (ROO^·^) radicals (sample peroxides) formed by iron, according to Fenton's reaction. These radicals are able to oxidize an alkyl-substituted aromatic amine (A-NH_2_), such as N,N-diethyl-para-phenylenediamine (DEPPD), transforming it into a derivative ([A-NH_2_^·^]^+^). With some modifications in the procedure, this method also is called as free oxygen radical test (FORT), which reflects levels of organic hydroperoxides. Results are expressed in arbitrary units, called Carratelli units (Carr U), where one Carr U corresponds to 0.08 mg/dL H_2_O_2_. Values greater than 300 Carr U suggest OS [99–101].

The OXY Adsorbent Test evaluates the antioxidant ability of each sample to oppose the oxidant action of hypochlorous acid (HClO) added in excess. The results are expressed as *μ*mol HClO/mL, and low values indicate a reduced antioxidant capacity [102].

Two aspects that we should highlight about this index is that it has only been used in serum as a biological sample and its calculation method, which makes its daily use difficult.

In this sense, due to dROMs and OXY are in different measurement units, it is necessary to have a standardization according to the following formula [101, 102]:

(3) $$\begin{array}{c} {sv}_{\mathrm{var}}=\frac{v_{\mathrm{var}}-{\bar{x}}_{\mathrm{var}}}{s_{\mathrm{var}}}, \end{array}$$

where *sv*_var_ is the standard value of each measurement which is equivalent to *z*, *v*_var_ is the original value of each subject, ${\bar{x}}_{\mathrm{var}}\;$ is the mean of the parameter, and *s*_var_ is the standard deviation of the parameter.

After this calculation, a simple subtraction between standardized dROM and standardized OXY must be calculated:

(4) $$\begin{array}{c} \text{Oxidative INDEX}={sv}_{\text{ROM}}-{sv}_{\text{OXY}}. \end{array}$$

This index has proven its usefulness in comparisons between young vs. old people [103], men vs. women [104], and in several clinical settings, such as cardiovascular diseases [98, 106, 107], oral diseases [105, 111], neurological disorders [108], hepatic diseases [109, 112], and cancer [110], showing that it has the capacity to discriminate between the groups (Table 7). It is important to note that since this index evaluates the oxidative balance, no other OS marker was included in any study.

In the interpretation, being a standardized value, there can be negative and positive values in a range of - 3 to +3, with 0 being the ideal balance between oxidants and antioxidants; therefore, positive values indicate predominant oxidized damage and negative values an imbalance towards antioxidants. Moreover, it has been shown that if the 75th percentile of the data is calculated, a cut-off value can be obtained to establish the intensity of the OS, at least by deciding whether it is low or high [102, 106], although further studies need be carried out to confirm their usefulness as a diagnostic tool [108].

Another index with total hydroperoxides measured by dROMs method has been described. For this index, the measurement of the biological antioxidant potential (BAP) is used as the antioxidant component.

In the BAP method, the ability of plasma antioxidant components to give reducing equivalents to reactive species is determinate using Fe^3+^ that are reduced to Fe^2+^. The values are expressed in terms of iron-reducing activity by vitamin C as standard, considering as cut-off values: >2200 *μ*mol/L as optimum status, <1600 *μ*mol/L as deficient status, and <1400 *μ*mol/L as high deficiency status [99, 113].

An arbitrary index is calculated as a ratio. This index has been used in two directions

- Oxidant to antioxidant (dROMs/BAP) ratio, if it considers that the balance is disrupted by excessive production of dROMs or by low BAP
- Antioxidant to oxidant (BAP/dROMs) ratio, when it wants to measure the relative antioxidant capacity

In addition, two modifications to the BAP/dROMs ratio have emerged. First a proposal of Yamamoto et al. (2015) called mOA that is an adjustment of the ratio by the constant 7.541 [114]:

(5) $$\begin{array}{c} \text{mOA}=\frac{\text{BAP}/\text{dROMs}}{7.451}. \end{array}$$

In the other modifications, each BAP/dROMs value is divided by mean BAP/dROMs ratio; in its interpretation, a cut-off value <1.0 indicates an antioxidant potential decreased [115]. This is the only study that establishes a cut-off value; others use the ratios as quantitative values. Both indexes aim to show more clearly the antioxidant potential.

These indexes have been used in research mainly in Japan; seven studies calculated the ratio in the oxidant direction to the antioxidant [99, 116–121], and six in the reverse direction [113, 122–124], including the investigations that calculated the modified ratios [106, 107] (Table 8). Only in one report the follicular fluid was used as a biological sample [116]; in the others, serum or plasma was used, and neither markers of the OS were included in the studies.

Also, it draws attention that some researchers call the dROMs/BAP ratio “oxidative stress index” (OSI) [99, 116, 119–121] as the first index that we included in this review, which is logical because this index is an oxidant/antioxidant ratio; the difference is the form of evaluation of both components. In both OSI, the oxidative part is the measurement of total peroxides or hydroperoxides, which are the oxidation products of several molecules, such as lipids, proteins, and amino acids; therefore, this is a measure of oxidative damage, what does not happen with the antioxidant capacity. The TAC and BAP have different principles, as well as their measurement units; both determinate the total nonenzymatic antioxidant capacity of biological samples, but although BAP is the most recommended procedures [125], the results of the different studies shown that this parameter almost does not change, making the index show no difference [114, 115, 122], especially when it is used to see the antioxidant potential (BAP/dROMs).

In summary, seems that the Oxidative INDEX can reflex the intensity of the OS better than the dROMs/BAP ratios, but these indexes do not include antioxidant enzymes, important elements in oxidative/antioxidative balance.

### 3.6. Oxidative Stress Profile

As we explained above, all the ratios and indexes proposal only measure extracellular OS components or oxidative/antioxidative capacities of the biological samples, so missing other elements that also participate in the oxidative balance.

Thus, in another attempt to measure oxidative/antioxidant balance, Cutler et al. in 2005 [126, 127] proposed the so-called “oxidative stress profile,” where it considers a lot of markers of oxidative damage, such as MDA, 4-HNE, hydroperoxides, isoprostanes, oxidized nucleic acids, protein carbonyls, antioxidants (endogenous and exogenous), and plus inflammation markers, such as interleukins, measured in both blood and urine samples. In total, these were suggested more than 50 elements in a proposal to assess the oxidative/antioxidative balance [127].

In the interpretation of the results, cut-off values are established for optimum health and intervals outside the reference ones, making a balance between the oxidized components through an oxidation index and an antioxidant index, obtaining an average level of antioxidant protection and other oxidative damages summarized in four categories: (1) high protection-high damage, (2) high protection-low damage, (3) low protection-low damage, and (4) low protection-high damage [126, 127]. The limitations of this proposal are the high cost due to the large number of parameters to be measured; many of which require a measurement with sophisticated equipment and techniques and also the need to establish the cut-off values to calculate the indexes, mainly of the exogenous antioxidant markers, which are dependent on the diet and lifestyle, so they cannot be generalized in the populations, and finally, the complexity in interpreting the results.

### 3.7. OXY Score

Follow-up with the same objective of creating an index to measure the OS, Veglia et al. in 2006 proposed the so-called OXY score, an index derived from the computation of different parameters of the oxidative balance. This score includes individual measurements in plasma of free and total MDA (F-MDA, T-MDA) and the GSSG and GSH levels and moreover the urinary isoprostane-PF2*α*-III level (iPF2*α*) as the combined oxidative damage. The antioxidant counterpart includes GSH, *α*- and *γ*-tocopherol, and antioxidant capacity measured by the OXY Adsorbent Test, which the authors called individual antioxidant capacity [128].

For calculation, first the F-MDA, T-MDA, iPF2*α*, and GSSG levels are log-transformed, then each marker must be standardized according to the formula previously describe, using the raw or log-transformed value as correspond. Later, the average of the standardized oxidants (damage score) and the average of the standardized antioxidants (protection score) must be calculated. Finally, the subtraction of a damage score minus protection score is the OXY score.

Same as for Oxidative INDEX, if the result is near to zero, the levels of all markers are near the average normal values or there is a balance between oxidants and antioxidants, and positive values indicate an imbalance towards the oxidants [128, 129]. It has been suggested to compute the OXY score without the urinary iPF2*α* as a modified OXY score, with similar results [130].

Although the ability of the OXY score to discriminate between different clinical conditions, age, and sex has been reported [127, 128], it is necessary to recognize that antioxidant enzymes are not included, being a score limiting [130]. Some drawbacks of the OXY score are the difficulty in calculating it, and the methods of measurement of all the markers are included, because they are complex and expensive; thus, it is not easily applicable to clinical and epidemiological settings.

Another modification to the OXY score is reported, which is a global score for the OS associated with albuminuria to their use in chronic kidney disease patients (Albumin OXY score). This index included protein carbonyl and MDA to calculate the damage score, and superoxide (O_2_^−·^) scavenging activity, catalase (CAT), and TAC as the protection index. For the calculating, the authors followed the procedures indicated for the Oxidative INDEX and the OXY score and proposed a new calculation that includes logarithmic transformation, standardization, and final subtraction, integrating both proposals. Being standardized values, the interpretation is equal to the two indexes previously mentioned [131]. The main clinical disadvantages of this index are the complication in the calculation, the cost, and the complexity of some measurements, although the total number of markers to be measured was reduced.

### 3.8. Oxidative Stress Score

All the indexes mentioned until now, except that proposed by Cutler and the Albumin OXY score, include an oxidant part and the non-enzymatic antioxidant capacity and do not take into account the antioxidant enzymes as part of the calculation. Thus, it seems necessary to include at least the main enzymes in the cell antioxidant process: superoxide dismutase (SOD), glutathione peroxidase (GPx), and/or catalase (CAT).

In this sense, Amstad et al. in 1991 [132] showed in transformed cell lines of mice that the balance between the activities of SOD and CAT+GPx is more important to determine the oxidative effect than the absolute activity of a single enzyme. Following this proposal, it has developed a theoretical model of biochemical interaction between these three enzymes showing an additive effect between SOD and GPx, and synergistic effects between GPx and CAT to ensure a global cell protection [133, 134]. As we know, the oxygen in aerobic organisms is part of various biochemical reactions, such as the mitochondrial respiratory chain and phagocytosis. One characteristic of these biochemical processes is the transformation of molecular oxygen into O_2_^−·^, which is dismuted by SOD, forming H_2_O_2_ in a first step. Although it is currently recognized that H_2_O_2_ is an important molecule in redox regulation and signaling processes, it is also known that it is a molecule harmful for its oxidizing capacity; thus, living organisms have a series of enzymes H_2_O_2_-removing to maintain balance, being glutathione peroxidases as the main second step [8, 135]. An imbalance between the first and second steps has as the potential result of increasing H_2_O_2_ levels, which is possible because the reaction constant of SOD is higher than GPx, so H_2_O_2_ is formed quickly and is not eliminated in its entirety; in addition, this disproportion in the activity between SOD and GPx in favor of the first can produce an increase in lipoperoxidation [136]; therefore, the inclusion of these enzymes as part of an integral OS measurement is important.

Another point to consider in the assessment of OS is to establish which cut-off values of the oxidative/antioxidative imbalance can be considered as harmful, to refer only to the oxidative distress. In this sense, in 2004, we proposed the integral and dynamic measurement of the OS through the evaluation of the antioxidant system efficiency, establishing the categories: (a) efficient antioxidant system (EAS) when the harmful action of the oxidants is effectively counteracted, (b) antioxidant enzymatic deficiency (ANED) if there is an inefficient or insufficient action of antioxidant enzymes, (c) exogenous antioxidants deficiency (EXAD) when there is an insufficient or inefficient action of non-enzymatic antioxidants causing oxidized the molecules, and (d) antioxidant system global deficiency (ASGD) if the enzymatic and nonenzymatic antioxidant components show an imbalance in favor of oxidized molecules [137, 138]. Later, we reorganized the interpretation considering the intensity of the OS, proposing four categories: without OS (WOS), low OS (LOS), moderate OS (MOS), and severe OS (SOS). About this, Lushchak in 2014 [139] also expressed the need to classify the OS based on the intensity, suggesting three levels: low, intermediate, and high, where the high can be interpreted as severe OS, very similar to our proposal.

In order to reach our classification, an index was calculated which we call the oxidative stress score (SS) [140]. The SS includes, originally, the measurement of plasma MDA and DNA damage, the activities of the SOD and GPx erythrocyte enzymes, the total antioxidant plasma status (TAS), and the calculation of the SOD/GPx ratio as a marker of oxidation, and the antioxidant gap (GAP). In this sense, GAP represents the antioxidant capacity of other nonmeasured plasma components different to albumin and uric acid, such as antioxidant vitamins, bilirubin, and hormones, can be calculated from TAS, and albumin and uric acid levels multiplied by their value of Trolox equivalent antioxidant capacity (TEAC) using the formula [10]:

(6) $$\begin{array}{c} \text{GAP}=\text{TAS}-\left[\left(\text{albumin}\times \text{TEAC}\right)+\left(\text{uric acid}\times \text{TEAC}\right)\right], \end{array}$$

where TAS, albumin, and uric acid are in *μ*mol/L, TEAC for albumin is 0.69, and TEAC for uric acid is 1.00.

Low-molecular-weight antioxidants such as GSH and thiols are not included in the score because both are measured in TAS, and CAT neither is considered due to its role as H_2_O_2_ removal is relative in humans, because it is a peroxisomal enzyme [8].

It was defined the cut-off values of each parameter based on the 90th percentile of healthy young subjects (20-45 y and both sexes), and a score 1 was given to each value above of cut-off (MDA, DNA damage, and SOD/GPx ratio) or under the cut-off (enzymatic and non-enzymatic markers), and 0 if the values are in normal range. After, a sum of all the components was obtained as a SS ranging from 0 to 7, representing the severity of the marker modifications. Then, the SS was categorized as follows [140]:

- WOS if SS = 0‐1
- LOS if SS = 2‐3
- MOS if SS = 4‐5
- SOS if SS = 6‐7

Also, we have excluded DNA damage because their procedure is not easily accessible; in this case, SS is ranging from 0 to 6 [141]. This index can be dichotomous, if SS ≤ 3, we consider as without OS or eustress, and if SS > 3, the subjects have high OS or distress [142].

The SS has been used in several studies since 2005. It has probed in young, mid-aged, and older subjects, both sexes and different study designs (Table 9). This index has shown to be useful to differentiate rural/urban residents [140] and pre/postmenopausal women [141, 146]. Also, it has been applied in cognitive impairment [138], chronic-degenerative diseases such as diabetes mellitus and arterial hypertension [142, 145], osteoporosis [143], and metabolic syndrome [144]. In addition, it has been used in clinical experimental studies demonstrating therapeutic [148] and of nutritional supplements effects [141, 151], as well as the efficiency of controlled physical exercise [147, 150].

Other authors have suggested similar proposals to the SS. Initially, Goodman et al. in 2007 [152] developed an OSS with the same idea of us in the form to compute it, but they included prooxidant and antioxidant factors, mainly from diet and lifestyle, to reflect a prooxidant/antioxidant exposure balance. This score is different to our proposal, because they do not perform measurements, rather they use questionnaires to obtain the information and based on them the calculation is made. As the main intention of this index is the exposure balance, the name has been changed to oxidative balance score (OBS). The OBS is a very different index to those included in this review, so we will not discuss it, but it is important to note that it has been used mainly in cases of cancer [141, 153–155] and chronic diseases [156–158], besides it has been related to OS markers [159, 160], inflammation markers [161, 162], then too it carried out studies from its relationship with some gene expression and genetic polymorphism [163–166].

On the other hand, based on the Goodman proposal [152], a study was carried out to determinate the association between adiposity measures and an OSS. The OSS was obtained from the blood measurements of GSH, GPx, vitamin C, MDA, and TAC, and some factors of lifestyle such as smoking, use of anti-inflammatory medication, nutritional supplement, and herbal product, adding a total of 9 components [167]. All the variables were categorized as 0 or 1, as we previously pointed. Although the idea is like to our SS, this index is more complex and included particular parameters such as the consumption anti-inflammatory medication and herbal product that are not necessarily useful in all the researchers.

Another study used six blood oxidative stress markers: MDA, ox-LDL, total antioxidant reactivity (TAR), SOD, GPx, and CAT, and those were scored as 0, 1, and 2 according the mean and standard deviation (SD) of healthy subjects. A score of 0 was assigned if the measurement of MDA, ox-LDL, CAT, or SOD (oxidants) was ranging mean + 1SD or if TAR and GPx (antioxidants) values were mean − 1SD. When the values were between +1SD and +2SD of the oxidants or −1SD and −2SD of the antioxidants, a value of 1 point was assigned. A value of 2 points was scored if the oxidant values were more than +2SD or the antioxidant values were less than −2SD. In this case, OSS ranging from 0 to 12 and in a dichotomous way, 6 is the cut-off [168]. This OSS seems more accessible to clinical research than the previous, but it needs to be tested in other clinical settings, since it has only been used in phenylketonuria.

In addition, in 2014, two variants of OSS have been published. The first is called OSI, because it analyzes the oxidant/antioxidant balance as the original OSI, but really is an index due to the oxidizing part which includes total lipoperoxides measured as their different forms (conjugated dienes, ketodienes, compounds with conjugated double bonds, conjugated trienes, and MDA), and in the antioxidant part, called the antioxidant defense (AOD), there is an evaluation of *α*-tocopherol, retinol, and reduced and oxidized glutathione levels plus SOD activity. First is necessary to obtain the result of the division between each value and the mean value of the control group of each parameter. After that, a multiplication of the oxidized components is placed in the numerator and the multiplication of the antioxidant counterpart in the denominator. As the control value is 1, in the interpretation, values above 1 indicate OS [169]. As the calculation is complex, their use seems impractical.

The last proposal includes 12 different blood OS parameters, four oxidized components (protein carbonyls, protein sulfhydryl, MDA, and 8-OHdG), and eight antioxidant components (SOD, GPx, glutathione reductase [GR], CAT, GSH, uric acid, bilirubin, and vitamin C). In the report, the calculation is not very explicit; only the authors explain that specific *Z*-scores of each parameter were computed, and after an OSS was calculated without explain how this is performed [170]; therefore, its application is doubtful.

One common point in all those proposals is that MDA is used as oxidized marker; this is possible because this parameter is an index of lipoperoxidation involved in both recent and chronic damages, so although much has been questioned, especially its form of measurement, is the marker that best reflects the oxidative state in any health situation or disease. The other point to highlight is the use of the main antioxidant enzymes, SOD and GPx, which importance was discussed at the beginning of this section, together the measurement of the antioxidant capacity, a parameter, in which all the indexes shown here are in agreement, independently of the method by it is evaluated.

Finally, all the proposals that were revised concord in an important aspect; the measurement of the involved oxidative stress markers as an indicator is more sensitive to the imbalance between oxidants and antioxidants than individual parameters.

Indeed, the SS is a proposal with the advantage that it is possible to increase the number of markers (and therefore the total score), both oxidized molecules and antioxidants, being able to include oxidized proteins, DNA damage, and other enzymes or antioxidant molecules; as long as the cut-off values are taken to decide when the marker is increased or decreased, as the case may be.

In this review, we analyzed the main indexes as indicators to measure oxidative stress grades linked to different clinical and health settings, highlighting the advantages and disadvantages of each of them. It should be noted that the modulating mechanisms of OS in humans are complex and interact with each other; hence, the SS can be useful to determine if an individual has a high level of OS and thus proceeds with a possible antioxidant therapy, thus improving your state of health and quality of life.

It is worth mentioning as a limitation of this review that the indexes for measuring oxidative stress analyzed here are not the only ones. We find in the literature review of others, such as the prooxidant-antioxidant balance (PAB) [171] and the oxidation-reduction potential (ORP) [172], which were not included because their fundaments are outside the scope of this work.

Despite the abundant information available so far, more clinical research is needed, especially on prognosis, to assess the usefulness of the different indices, and comparison studies between them, as well as an analysis of the literature of the indicators that evaluate both components, oxidant and antioxidant in a single measurement, as in the PAB, and the redox potential of a sample, such as the ORP.

## 4. Conclusion

We made a review of the state of the art of knowledge about the measurement of OS in humans, focused on different ways to integrally evaluate it.

Regardless of the components for the calculation of the different indexes analyzed, all have been able to demonstrate that they can differentiate the OS in pathological or biological stress states; however, not all measure both intracellular and extracellular components involved in the OS; thus, we think they are not equivalent. We cannot say that one index is the best for measuring OS, but we can suggest that there are some proposals that, despite being different, seem to be equivalent in their interpretation since they allow differentiating the types of OS and establishing an intensity or severity, such as Oxy-score and the different proposals of oxidative stress score.

Perhaps an interesting way to make this evaluation is to combine the measurement of various markers, both oxidized and antioxidant molecules (enzymatic and non-enzymatic), besides a simple way to put them together through an index whose calculation is not complicated, such as the oxidative stress index, which has demonstrated to be reliable, practical, and with clinical utility.

## Figures and Tables

**Table 1:** Biomarkers used in the oxidative stress measurement in human fluids.

Oxidation markers
*Lipids*
(i) Conjugated dienes (CD)
(ii) Aldehydic products (a) Malondialdehyde (MDA)^∗^ (b) 4-hydroxy-2-nonenal (4-HNE)
(iii) Alkane exhalation
(iv) Total hydroperoxides (LOOH)
(v) Oxidized LDL (oxo-LDL)
(vi) F2-isoprostanes (F2-Iso)
(vii) Advanced lipid peroxidation end products (ALEs)
*DNA*
(i) Nucleotide oxidation (a) 8-hydroxy-guanosine (8OHG) (b) 8-hydroxy-desoxyguanosine (8OHdG) (c) Thymidine glycol (d) 7,8-dihydroxy-8-oxo-2′-deoxyguanosine (8oxodG) (e) 5-chlorouracil
(ii) Deoxyribose oxidation
*Proteins*
(i) Protein carbonyls
(ii) Advanced oxidation protein products (AOPP)
(iii) Amino acid oxidation (a) 3-nitro-tyrosine (b) 3-chloro-tyrosine
(iv) Ischemia-modified albumin (IMA)
(v) Advanced glycation end products (AGEs)
Oxidants
(i) Hydrogen peroxide (H_2_O_2_)
(ii) Superoxide anion radical (O_2_^−·^)
(iii) Nitric oxide (NO^·^)
Antioxidant markers
*Nonenzymatic*
(i) Vitamins (A, C, E)
(ii) Metals (Se, Zn)
(iii) Glutathione and cysteine
(iv) Uric acid
*Enzymes*
(i) Superoxide dismutase (SOD)
(ii) Glutathione peroxidase (GPx)
(iii) Glutathione reductase (GR)
(iv) Catalase (CAT)
*Nonenzymatic antioxidant capacity*
(i) Oxygen radical antioxidant capacity (ORAC)
(ii) Total radical-trapping antioxidant parameter (TRAP)
(iii) Total antioxidant capacity (TAC)
(iv) Total antioxidant status (TAS)
(v) Ferric reducing antioxidant potential (FRAP)
(vi) Antioxidant gap
(vii) Biological antioxidant potential (BAP)

^∗^We consider MDA and TBARS as the same because TBARS is the procedure most used to measure MDA.

**Table 2:** Characteristics and findings of studies that used different individual oxidant and antioxidant biomarkers and calculated a ratio.

Author, year [reference]	Study design/country	Population (age, sex)/sample size	Biological sample/ratio used	Clinical event	Results	Conclusion
Pabón A. et al., 2003 [17]	Cross-sectional/Colombia	Adults (15-37 y, both sexes)/84 subjects with malaria 23 control subjects	Plasma, erythrocyte/MDA:TAS MDA : GPx	Noncomplicated malaria	MDA/TAS index was 3.5-fold more in patients. MDA/GPx index was increased by 6-fold.	High OS in patients with malaria. Recommended MDA/TAS as OS marker.
Harma M. et al., 2003 [18]	Cross-sectional/Turkey	Adults (mean 30 y, female)/38 women with CHM 31 healthy subjects	Plasma/FRAP : TP	Complete hydatidiform mole (CHM)	The mean FRAP/TP index was higher in patients with CHM than in healthy pregnant women.	The authors did not conclude about the FRAP/TP ratio.
Kocyigit A. et al., 2004 [19]	Cross-sectional/Turkey	Children (2-13 y, both sexes)/42 asthmatic children 30 healthy children	Plasma/MDA : TAC	Asthma	MDA/TAC ratio was significantly higher in patients.	The authors did not conclude about the MDA/TAC ratio directly.
Agha-Hosseini F. et al., 2012 [20]	Cross-sectional/Iran	Adults (25-82 y, both sexes)/32 subjects with OLP 26 subjects with OSCC 30 healthy subjects	Saliva/FRAP : MDA	Oral lichen planus (OLP) Oral squamous cell carcinoma (OSCC)	FRAP/MDA ratio was lower in patients with OSCC than both OLP and control. FRAP/MDA ratio was lower in patients with OLP compared to control.	Patients with OLP and OSCC are susceptible to an imbalance of antioxidant-OS.
Venturini D. et al. 2015 [21]	Intervention study/Brazil	Adults (51 ± 8 y, both sexes)/21 subjects in fish oil group 26 subjects in fish oil+extra virgin olive oil group 13 subjects in extra virgin olive oil group 42 control subjects	Plasma/TRAP : AOPP AOPP : TRAP	Metabolic syndrome (MetS)	There was an increase in TRAP/AOPP index and a decrease in AOPP/TRAP index in users of fish oil-extra virgin olive oil (FOO) compared with fish oil.	The fish oil and extra virgin olive oil have beneficial synergistic effects on OS, using the ratio, in patients with MetS.
de Almeida J. P. et al., 2016 [22]	Cross-sectional/Brazil	Adults (46-60 y, both sexes)/32 subjects with HCV+HypoD 26 subjects with HCV+Normal D 89 control subjects	Plasma/AOPP : TRAP	Chronic hepatitis C (HCV) and hypovitaminosis D (HypoD)	HCV patients with hypovitaminosis D had higher AOPP/TRAP.	The authors did not conclude directly about the AOPP/TRAP.
Costa NT. et al., 2016 [23]	Cross-sectional/Brazil	Adults (18-70 y, both sexes)/91 RA patients without IR 82 RA patients with IR 97 healthy	Plasma/AOPP : TRAP	Rheumatoid arthritis (RA) and insulin resistance (IR)	The patients with IR had higher AOPP/TRAP compared to the patients without IR and not using antitumor necrosis factor-*α*.	The authors did not conclude directly about the AOPP/TRAP.
Becatti M. et al., 2018 [24]	Longitudinal/Australia	Adults (35 ± 3 y, female)/45 infertile women 45 control women	Plasma, follicular fluid (FF)/ORAC : MDA	Infertile women undergoing assisted reproductive technology procedures	The plasma ORAC/MDA ratio was about 3.4-fold lower than the control, and the FF ratio was about sixfold lower than the control. Both the plasma and FF ratios results were correlated.	The use of these ratios could help define the critical role of OS markers and their optimum levels in the female reproductive system.

AOPP: advanced oxidation protein products; FRAP: ferric reducing antioxidant potential; GPx: glutathione peroxidase; MDA: malondialdehyde; OS: oxidative stress; TAC: total antioxidant capacity; TAS: total antioxidant status; TP: total peroxides; TRAP: total radical-trapping antioxidant parameter.

**Table 3:** Characteristics and findings of studies that used reduced to oxidized glutathione ratio (GSH/GSSG).

Author, year [reference]	Study design/country	Population (age, sex)/sample size	Biological sample/other markers	Main outcome	Results	Conclusion
Paolisso G. et al., 1996 [33]	Experimental study with free fatty acids (FFA) infusions/Italy	Adults (33 ± 1 y, both sexes)/10 subjects with intralipid continuum infusion 9 subjects with 6 h intralipid infusion 11 subjects with insulin infusion	Plasma/MDA, LOOH	Effect of FFA on OS	The GSH/GSSG ratio diminished after 6 h of FFA infusion and continued to 24 h, although the better effect was at 6 h. After a wash-out period, the ratio returns to basal value.	The OS intensifies with the increase of FFA, inferring that the ratio measures the OS.
Abramson JL. et al., 2005 [34]	Cross-sectional/USA	Adults (44 ± 9 y, both sexes)/126 subjects without CHD	Plasma/FORT	Association between OS and high sensitivity CRP (hsCRP)	The GSH/GSSG ratio showed little association with hsCRP.	The ratio was not strongly associated with CRP.
Gherghel D. et al., 2005 [35]	Cross-sectional/UK	Older adults (70 ± 10 y, both sexes)/21 POAG patients 34 control subjects	Blood/none	Glutathione status in primary open-angle glaucoma (POAG)	The GSH/GSSG ratio was similar in both sexes. No differences between study group and control were observed in redox index, after correction for age and gender.	The authors did not conclude about the ratio because the results were not significant.
Yeh CC. et al., 2006 [36]	Cross-sectional/Taiwan	Adults (27-83 y, female)/112 women with BC 40 control subjects	Blood, tumor tissue/none	Glutathione status in breast cancer (BC)	GSH/TGSSG ratio was decreased in the blood of the patients with breast cancer, and breast cancer tissue was increased, especially in stage II.	There was an imbalance in the glutathione redox status in the breast tumor patients.
Ashfaq S. et al., 2006 [37]	Cross-sectional/USA	Adults (30-65 y, both sexes)/114 healthy subjects	Blood/cysteine and its redox state	Thiols status and early atherosclerosis	After adjusting for traditional risk factors and hs-CRP, the GSH/GSSG ratio is an independent predictor of intima-media thickness.	Glutathione redox state is an independent predictor for the presence of early atherosclerosis.
Nikolaidis MG. et al., 2007 [38]	Experimental of isokinetic exercise/Greece	Adults (23 ± 2 y, female)/12 women in isokinetic exercise	Blood/MDA, TAC, PC	Effect of repeated muscle-damaging exercise on the time-course changes in indices of muscle damage	Exercise decreased the GSH/GSSG ratio at several time points after both exercise bouts.	The authors did not conclude directly with the ratio but shown an effect across it.
Harzallah O. et al., 2008 [39]	Cross-sectional/Tunisia	Adults (22–61 y, both sexes)/40 subjects with BD 40 healthy subjects	Blood/MDA, CAT, SOD, GPx	Association between OS and Behçet's disease (BD)	The GSH/GSSG ratio was reduced in BD patients.	Exist an OS in BD as shown by the diminution of the ratio.
Youssef H. et al., 2009 [40]	Cross-sectional postexercise effect/Lebanon	Adolescents (14-19 y, female)/29 overweight girls 17 normal weight girls	Blood/F2-Iso, LOOH, ox-LDL, SOD, GPx, vit. C, vit. E, *β*-carot.	Effect of incremental ergocycle exercise on OS	The overweight adolescents presented the GSH/GSSG ratio lower. No changes for GSH/GSSH were observed in both groups after exercise.	Exercise exacerbates OS in the overweight adolescents; GSH/GSSG ratio is a good marker.
Tsai SM. et al., 2009 [41]	Cross-sectional/Taiwan	Adults (31-81 y, both sexes)/54 HBV-associated HCC patients 57 control subjects	Blood/ O_2_^·^−, MDA, SOD, GPx, vit. A, C, E	Changes in OS in chronic hepatitis B virus (HBV)-associated hepatocellular carcinoma (HCC) patients	The ratio of GSH/GSSG of HBV-associated HCC patients was lower than that of controls.	Changes in the GSH demonstrated that HBV-associated HCC patients had undergone OS during their clinical disease progression.
Huang YS. et al., 2009 [30]	Cross-sectional/China	Adults (41-76 y, both sexes)/56 patients plaque-forming 42 patients with intima-media thickening 34 subjects with normal intima-media	Plasma/MDA, ox-LDL	Relationship between glutathione redox status and the atherosclerosis	GSH/GSSG redox status was positively correlated with the intima thickness.	Peroxidative glutathione redox status may be a sensitive and reliable index for monitoring OS in atherosclerosis.
Kleinsorge EC. et al., 2011 [42]	Cross-sectional/Argentina	Adults (23 ± 2 y, both sexes)/26 photocopy machine operators 52 control subjects	Erythrocyte/MDA, CAT, DICA	To determinate the oxidative damage in photocopier operators	GSH/GSSG is similar between operators and controls.	The authors did not discuss or conclude about the ratio.
Patel RS. et al., 2011 [43]	Cross-sectional/USA	Adults and elderly (20-70 y, both sexes)/169 healthy subjects	Plasma/cysteine ratio, dROMs	Relationship between novel markers of OS and arterial elastic properties	The redox potential of GSH/GSSG showed modest association with carotid-femoral pulse wave velocity.	After multivariate adjustment for confounders, GSH/GSSG is not associated with impaired arterial elasticity.
Zepeda RJ. et al., 2012 [44]	Prospective randomized single blind clinical trial/Chile	Adults (30-75 y, both sexes)/23 patients with EH in carvedilol therapy 21 patients with EH in nebivolol therapy 30 normotensive patients	Erythrocyte/NOx, FRAP, MDA, 8-Iso	Effect of carvedilol and nebivolol on the OS and endothelial function in patients with essential hypertension (EH)	In patients with carvedilol, GSH/GSSG ratio was higher compared with patients who received nebivolol.	As part of the antihypertensive mechanism, carvedilol seems to reinforce the antioxidant system.
Llorente-Cantarero FJ. et al., 2012 [45]	Cross-sectional/Spain	Children (7-12 y, both sexes)/138 healthy subjects	Erythrocyte/MDA, 4-HNE, PC, NOx, SOD, GPx	To identify gender-based biomarkers for OS in children	Girls had a lower GSH/GSSG ratio than boys.	The authors did not conclude about the ratio directly.
Ntalapascha M. et al., 2013 [46]	Observational prospective/Greece	Adults (30-65 y, male)/18 patients with severe OSAS 13 control subjects	Erythrocyte/MDA, PC, 8-Iso, CAT, SOD, TAC	Assess the OS in patients with obstructive sleep apnea syndrome (OSAS)	The overnight (morning–night) change (%) of GSH/GSSG ratio was different between OSAS and controls.	OSAS might be associated with increased OS possibly via GSH/GSSG pathway.
Shah D. et al., 2013 [47]	Cross-sectional/India	Adults (29 ± 8 y, both sexes)/40 patients with SLE 40 healthy subjects	Erythrocyte/MDA, SOD, CAT, GPx, ROS production, total thiols	Association between glutathione pathway and systemic lupus erythematosus (SLE) severity	Altered redox state (GSH/GSSG) was found in SLE patients. The severity of disease was allied with altered GSH/GSSG.	GSH depletion may be coupled with the severity of the disease.
Victor VM. et al., 2014 [48]	Observational prospective of testosterone (T) therapy/Spain	Adults (29 ± 10 y, transsexual)/57 female-to-male transsexuals (FtMs)	Polymorpho-nuclear cells/mitochondrial O_2_ consumption, membrane potential, ROS production	The effect of T treatment on the redox state of leukocytes of FtMs subjects	GSH/GSSG ratio was lower in patients post-T treatment	Treatment of FtMs with T can induce a state of OS.
Enns GM. et al., 2014 [49]	Cross-sectional/USA	All ages (0.5-50 y, both sexes)/62 subjects with mitochondrial disease (MitD) 59 healthy subjects	Blood/none	To determine the redox status in mitochondrial diseases and if the redox imbalance is an indicator of clinical status	The GSH/GSSG ratio was lower in MitD patients, and redox potential was higher compared with controls.	GSH and GSSG levels may be biomarkers of mitochondrial dysfunction, and the evaluation of redox potential may be useful in monitoring of clinical status in MitD.
Schmitt B. et al., 2015 [50]	Randomized crossover trial/France	Adults (38-67 y, both sexes)/20 subjects with metabolic syndrome	Blood/reduced thiols, vit. E	To compare the effect on glutathione status of a sublingual form of GSH, compared with oral GSH and N-acetylcysteine (NAC)	A higher GSH/GSSG ratio was observed in sublingual GSH group compared with oral GSH and NAC groups.	The authors conclude about the effectivity of the treatments based in the GSH results.
Karimi R. et al., 2016 [51]	Cross-sectional/USA	Adults (48 ± 28 y, both sexes)/268 subjects	Blood/none	Seafood Hg exposure is related to a shift in redox status (low GSH/GSSG or high redox potential (*E*_h_))	Blood Hg concentration was associated with changes in GSH/GSSG ratio and redox potential.	Hg exposure from seafood is linked to a shift in redox status toward OS.
Atkin M. et al., 2016 [52]	A double-blind, placebo-controlled crossover pilot/UK	Adults (18-70 y, both sexes)/26 subjects with T2DM	Blood/LOOH, TAS	Aged garlic extract (AGE) may improve OS in high-risk cardiovascular subjects with type 2 diabetes (T2DM)	No difference was found in GSH/GSSG ratio.	There is no clinical benefit of adding AGE, in the short term, to usual medical therapy in patients with T2DM.
Galicia-Moreno M. et al., 2016 [53]	Cross-sectional/Mexico	Adults (43 ± 1 y, both sexes)/57 subjects with ALC 130 control subjects	Blood/MDA, PC	To evaluate the role of OS in alcoholic liver cirrhosis (ALC)	There were no differences in the GSH/GSSG ratio between groups. Active alcohol consumption had a tendency to produce glutathione oxidation.	ALC subjects have an increase in OS in the early stages of disease severity and abstinence from alcohol consumption favors GSH in patients with advanced disease severity.
Moreno-Solís G. et al., 2017 [54]	Observational/Spain	Infants (1-11 months, both sexes)/45 infants with RSV-AB 27 healthy infants	Erythrocyte/MDA, 4-HNE, GPx	Association between OS and acute bronchiolitis caused by respiratory syncytial virus (RSV-AB) and its severity	The GSH/GSSG ratio was lower in RSV-AB infants and decrease if the infants need oxygen therapy.	The authors propose the use of GSSG and the GSH/GSSG ratio as biomarkers linked to the pathogenesis of RSV-AB.
Vacchi-Suzzi C. et al., 2018 [55]	Cross-sectional cohort/USA	Adults (≥18 y, both sexes)/282 healthy	Blood/none	Association between blood lead level (BLL) and glutathione (GSH) redox biomarkers	Increasing exposure to Pb was associated with lower levels of GSH/GSSG ratio and more positive GSH redox potential.	Blood Pb is associated with lower levels of GSH and the GSH/GSSG ratio.

*β*-carot.: *β*-carotene; CAT: catalase; CHD: coronary heart disease; dROMs: derivatives of reactive oxygen metabolites; DICA: damage index by Comet assay; F2-Iso: 15 F2*α*-isoprostanes; FORT: free oxygen radical test; FRAP: ferric reducing antioxidant potential; GPx: glutathione peroxidase; GR: glutathione reductase; GST: glutathione-S-transferase; 4-HNE: 4-hydroxyalkenal; hsCRP: high sensitivity C-reactive protein; 8-Iso: 8-isoprostanes; LOOH: lipid hydroperoxides; MDA: malondialdehyde; NOx: nitrite plus nitrate; O_2_^−·^: superoxide anion radical; OS: oxidative stress; ox-LDL: oxidized low-density lipoprotein; PC: protein carbonyls; ROS: reactive oxygen species; SOD: superoxide dismutase; TAC: total antioxidant capacity; TAS: total antioxidant status; TGSSG: total glutathione; vit. A: vitamin A; vit. C: vitamin C; vit. E: *α*-tocopherol.

**Table 4:** Characteristics and findings of studies that used oxidized to reduced glutathione ratio (GSSG/GSH).

Author, year [reference]	Study design/country	Population (age, sex)/sample size	Biological sample/other markers	Main outcome	Results	Conclusion
Németh I. 1994 [31]	Prospective longitudinal/Hungary	Infants (gestational age 26-34 weeks, both sexes)/25 newborn premature infants with IRDS 20 premature infants as control	Blood/none	GSSG/GSH in the blood could be an index of O_2_ toxicity in pathological status of premature newborns.	Premature infants with IRDS have GSSG/GSH ratio higher than control newborns. A negative correlation between the redox ratio and the arterio-alveolar oxygen ratio was found.	It is recommended the use of the blood GSSG/GSH ratio as a noninvasive measure of *in vivo* OS.
Papp A. et al., 1999 [32]	Cross-sectional/Hungary	Infants who had been born prematurely (6 weeks-6 y, both sexes)/12 infants with active ROP 38 patients with ROP 56 control subjects	Erythrocyte/none	Glutathione status of erythrocytes in patients with retinopathy of prematurity (ROP)	Infants with active disease have the highest GSSG/GSH ratio.	The GSSG/GSH ratio may be a screen for active ROP in premature infants.
Annuk M. et al., 2001 [58]	Cross-sectional/Sweden	Adults (66 ± 10 y, both sexes)/37 subjects with CRF 37 control subjects	Serum/CD, MDA, LOOH, TAA, LPF	Relationship between OS and endothelial function in chronic renal failure (CRF)	The GSSG/GSH ratio was lower in patients with CRF. Endothelium-dependent vasodilation was negatively correlatedGSSG/GSH.	The authors did not conclude directly about the ratio, but they assume that this ratio is a marker of OS.
Sáez GT. et al., 2004 [59]	Observational prospective of antihypertensive treatment/Spain	Adults (mean 46 y, both sexes)/36 subjects with *β*-blockers 33 subjects with telmisartan 20 subjects without treatment	Blood, peripheral mononuclear cells/MDA	Impact of antihypertensive treatments on OSOS	After 3 months of antihypertensive treatment, the GSSG/GSH ratio was reduced; the beneficial effect of treatment increases over time.	The authors did not conclude directly about the ratio. Antihypertensive treatment improved the increased OS.
Skalicky J. et al., 2008 [60]	Cross-sectional/Czech Republic	Adults (51 ± 9 y, both sexes)/20 subjects obese with MetS 20 subjects obese without MetS 48 controls	Blood/TAS, vit. E, MDA, allantoin, *α*_1_-AP	OS in obesity with and without metabolic syndrome (MetS)	The obese patients with MetS have the highest GSSG/GSH ratio.	The authors did not conclude directly about the ratio, but they indicate that imbalance oxidative/antioxidative status is a risk in obese adults.
Lind L. et al., 2008 [61]	Prospective study/Sweden	Older adults (70 y, both sexes)/1016 subjects	Serum/CD, ox-LDL, TAC, homocysteine	Relationship between OS and brachial artery intima-media thickness (IMT) and grey scale median of the intima-media complex (IM-GSM)	The GSSG/GSH ratio was related to brachial artery IM-GSM after adjustment of traditional risk factors and inflammatory markers.	The low levels of the ratio indicate a reduced antioxidant activity.
Mercken EM. et al., 2009 [62]	Experimental/Netherlands	Adults (56 ± 7 y, both sexes)/15 COPD patients 10 controls	Erythrocyte/MDA, uric acid	OS is differentially triggered by contracting peripheral muscles in COPD patients compared with controls	GSSG/GSH ratio tended to be increased in COPD patients and tended to be increased immediately after exercise.	The authors did not conclude directly about the ratio, although they discuss that the ratio slightly increased in COPD patients after exercise.
Real JT. et al., 2010 [63]	Cross-sectional/Spain	Adults (40 ± 13 y, both sexes)/30 patients with HF 30 controls	Circulating mononuclear cells (CMC)/MDA, XO, SOD, CAT, GPx	Analyze the OS levels in CMC from familiar hypercholesterolemia (HF) patients and controls	GSSG/GSH ratio was significantly higher in FH patients compared with controls.	The authors did not conclude directly about the ratio, but they affirm that there is an important alteration of OS regulation in FH.
Rusanova I. et al., 2010 [64]	Cross-sectional/Panama	Children (6 months-15 y, both sexes)/95 patients with SCD 40 healthy	Erythrocyte/MDA, 4-HNE, NOx, GPx, GR, SOD	To correlate *β*-globin gene haplotypes with the OS in pediatric patients with sickle cell disease (SCD)	GSSG/GSH ratio was higher in patients with SCD.	Based on the ratio, the findings support the existence of oxidative damage in sickle cells.
Petrillo S. et al., 2013 [65]	Cross-sectional/Italy	All (4-64 y, does not specify sex)/14 patients with X-ALD 30 healthy	Lymphocyte Erythrocyte/total thiols, PC, SOD, GPx	Define the role of the glutathione in X-linked adrenoleuko-dystrophy (X-ALD)	The GSSG/GSH ratio was increased in patients with adrenomyelo-neuropathy.	The balance among glutathione forms is a hallmark and a potential biomarker of the X-ALD.
De Tursi Ríspoli L. et al., 2013 [66]	Observational prospective of bariatric surgery/Spain	Adults (43 ± 1 y, both sexes)/28 patients with morbid obesity	Erythrocyte/MDA, 8-oxo-dG	Assessment of OS variations and its relationship with the weight loss after a duodenal crossing surgical	The GSSG/GSH ratio diminished across one year from 3 months after the surgery.	The authors did not conclude directly about the ratio, but they infer that weight loss improves antioxidant status.
Bagan J. et al., 2014 [56]	Observational/Spain	Adults (61 ± 10 y, sex is not specified)/24 patients treated with intravenous bisphosphonates (ivBPs) and BRONJ 20 patients treated with ivBPs and without BRONJ 17 controls	Serum Saliva/MDA, 8-oxo-dG	Changes of OS in patients with bisphosphonate-related osteonecrosis of the jaw (BRONJ)	The GSSG/GSH ratio was a prognostic factor for the development of BRONJ after adjusted by confounders.	The GSSG/GSH was a significant factor predicting the development of BRONJ.
Blasco H. et al., 2017 [67]	Preliminary study/France	Adults (65 ± 14 y, both sexes)/10 ALS patients 10 controls	Blood/MDA, 8-OHdG, TAS	Association of OS and amyotrophic lateral sclerosis (ALS)	Higher GSSG/GSH ratio in ALS patients and correlations were found between the ratio and clinical markers.	The systemic alteration of the redox status in ALS patients was confirmed.
Arana C. et al., 2017 [57]	Observational/Spain	Adults (18-65 y, both sexes)/24 patients with T2DM and good metabolic control 27 patients T2DM with poor metabolic control 19 nondiabetic patients	Saliva/GPx, GR	Association between OS and periodontal disease in type 2 diabetes mellitus (T2DM) patients	Both diabetic groups showed higher GSSG/GSH quotients, being higher ratio in diabetic patients with poor metabolic control.	Poor metabolic control in T2DM patients is associated with higher levels of salivary OS.
Bellanti F. et al., 2018 [68]	Population-based cross-sectional study/Italy	Older adults (77 ± 6 y, both sexes)/48 sarcopenic 67 nonsarcopenic	Blood/MDA, 4-HNE	Association between OS and sarcopenic obesity in terms of glutathione balance	Sarcopenic had GSSG/GSH ratio higher than nonsarcopenic patients. There is a strong association between the Framingham CVD risk and GSSG/GSH in the sarcopenic-obese patients.	Redox balance analysis would be a useful part of a multidimensional evaluation in aging.

*α*_1_-AP: *α*_1_-antiproteinase; CD: conjugate dienes; GPx: glutathione peroxidase; GR: glutathione reductase; 4-HNE: 4-hydroxyalkenal; 8-OHdG: 8-hydroxy-2′-deoxyguanosine; LOOH: lipid hydroperoxides; LPF: oxidation resistance of lipoprotein fraction; MDA: malondialdehyde; NOx: nitrite plus nitrate; 8-oxo-dG: 8-oxo-deoxiguanosina; OS: oxidative stress; ox-LDL: oxidized low-density lipoprotein; PC: protein carbonyls; SOD: superoxide dismutase; TAA: total antioxidant activity; TAC: total antioxidant capacity; TAS: total antioxidant status; vit. E: *α*-tocopherol; XO: xanthine oxidase.

**Table 5:** Studies that have used the thiol ratios to measure the oxidative stress by age group and clinical event studied.

Age group	Clinical event	Number or studies
Adults (18-65 y)	Alterations in the pregnancy	8
	Cardiovascular diseases	4
	Respiratory diseases	4
	Skin disorders	4
	Psychiatric disorders	3
	Metabolic disorders	3
	Surgery	2
	Neurologic disorders	2
	Other disorders	10

Older adults (>65 y)	Ocular disorders	3
	Different events	4

Children (<18 y)	Neurologic disorders	4
	Genetic disorders	3
	Different events	8

Adults-older adults (>18 y)	Different events	3

Children-adults (<65 y)	Neurologic disorder	1

Total		66

**Table 6:** Studies that use the oxidative stress index (OSI) as a marker of oxidative stress separated by country, age group, and clinical event.

Country	Frequency (%)	Age group	Frequency (%)	Clinical event	Frequency (%)
Turkey	257 (85.1%)	Adults (18–65 y)	190 (63.0%)	Cardiovascular diseases	38 (12.6%)
Poland	13 (4.3%)	Adults-older adults (>18 y)	40 (13.2%)	Alterations in the pregnancy	32 (10.6%)
China	11 (3.7%)	Children (2–12 y)	35 (11.6%)	Psychiatric disorders	27 (9.0%)
India	4 (1.3%)	Neonates/infants (<2 y)	19 (6.3%)	Infectious diseases	17 (5.6%)
Estonia	3 (1.0%)	Older adults (>65 y)	8 (2.6%)	Cancer	15 (5.0%)
Thailand	3 (1.0%)	Adolescents (12–17 y)	5 (1.6%)	Skin disorders	15 (5.0%)
Ethiopia	2 (0.7%)	Children-adolescent (<18 y)	2 (0.7%)	Oral diseases	15 (5.0%)
Egypt	2 (0.7%)	Children to adults (<65 y)	2 (0.7%)	Respiratory diseases	14 (4.6%)
Romania	1 (0.3%)	All age groups	1 (0.3%)	Metabolic disorders	12 (4.0%)
Mexico	1 (0.3%)	Total	302	Other disorders	117 (38.6%)
Austria	1 (0.3%)			Total	302
Korea	1 (0.3%)				
Iran	1 (0.3%)				
Nigeria	1 (0.3%)				
Serbia	1 (0.3%)				
Total	302				

**Table 7:** Characteristics and findings of studies that used Oxidative INDEX (ROM/OXY ratio).

Author, year [ref]	Study design/country	Population (age, sex)/sample size	Main outcome	Results	Conclusion
Vassalle C et al., 2008 [98]	Cross-sectional/Italy	Adults (65 ± 10 y, both sexes)/100 CAD patients 70 controls	Relationship between Oxidative INDEX and cardiovascular risk factors in coronary artery disease (CAD).	The Oxidative INDEX was higher in CAD patients, correlated with aging and increased with the number of cardiovascular risk factors.	Oxidative INDEX could represent a valuable tool in clinical setting.
Vassalle C et al., 2009 [103]	Cross-sectional/Italy	Adults (16-79 y, both sexes)/179 subjects	Obesity and smoking may accelerate the increase of the OS related to advancing age.	Obesity and smoking are risk factors for elevated Oxidative INDEX. The index steadily rises at a mean rate of 5.3% (0.017 AU) per year in the overall population.	The authors did not conclude directly about the index, but they infer that OS increases due the risk factors analyzed.
Vassalle C et al., 2011 [104]	Cross-sectional/Italy	Adults (48 ± 12 y, both sexes)/116 males 216 females	Gender-related difference of OS	Age, high blood pressure, and smoking habit as factors associated with the index in men. Cigarette smoking and age are risk factors for an elevated OS in women.	The authors did not conclude directly about the index, but they infer that there is a difference in OS due to sex.
Tamaki N. et al., 2011 [105]	Observational longitudinal/Japan	Adults (44 ± 19 y, both sexes)/22 with CP 22 controls	To monitor OS in subjects with chronic periodontitis (CP) following nonsurgical periodontal treatment	Patients with CP have higher OS than healthy subjects. Periodontal treatment was associated with a reduction in the index.	Nonsurgical periodontal treatment was effective in decreasing the index.
Vassalle C et al., 2012 [106]	Observational Longitudinal Retrospective/Italy	Adults (67 ± 11 y, both sexes/97 CAD patients	Prognostic of OS on the rate of major adverse cardiovascular events (MACE) in CAD patients	High level of the index is an independent predictor of MACE.	OS (calculated by the index) may be a useful tool to predict MACE in CAD patients.
Vassalle C et al., 2012 [107]	Case-control/Italy	Adults (66 ± 9 y, both sexes)/55 post-women 108 men 72 CAD patients	To assess oxidative stress status in coronary artery disease (CAD) patients according to gender	There is a difference in the index between men and women without CAD. Women with CAD had the index higher than men.	The index can be a predictor of CAD in women.
Capaccio P et al., 2012 [108]	Cross-sectional/Italy	Adults (47 ± 14 y, both sexes)/39 ISSNHL patients 70 healthy subjects	To evaluate role of OS in idiopathic sudden sensorineural hearing loss (ISSNHL)	The index was higher in patients than in controls.	In ISSNH, there is an imbalanced oxidative status.
Terao M et al., 2014 [109]	Cross-sectional/Japan	Adults (46-61 y, both sexes)/29 with potential POPH 32 with potential non-POPH	Association between the OS and portopulmonary hypertension (POPH) in cirrhotic patients	Potential POPH patients have higher oxidative index values.	The oxidative/antioxidative balance was exacerbated in patients with potential POPH.
Uchida D et al., 2016 [110]	Translational/Japan	Adults (27-90 y, both sexes)/84 CC patients 80 PC patients	OS balance in pancreatic cancer (PC) and cholangiocarcinoma (CC) patients	The index was higher in CC patients with poor outcomes and not different in PC patients.	OS balance was dysregulated in CC patients with poor outcome.
Mizuno H et al., 2017 [111]	Randomized clinical trial/Japan	Adults (61 ± 10 y, both sexes)/T2DM and CP patients. 20 periodontal treatment 17 control group	Effect of nonsurgical periodontal treatment on OS balance patients with type 2 diabetes mellitus (T2DM) compared to no periodontal treatment	Systemic OS balance improved in the periodontal treatment group compared to the control group at 3 months.	In T2DM patients, nonsurgical periodontal treatment improved systemic OS.
Shimomura Y et al., 2017 [112]	Observational Longitudinal/Japan	Adults (>40 y, both sexes)/14 patients with NAFL 44 patients with NASH 11 patients with NASH-related HCC 15 healthy	Correlation between OS-related markers and the clinical characteristic in nonalcoholic fatty liver disease (NAFL) and to characterize the OS balance in NAFL, nonalcoholic steatohepatitis (NASH), and NASH-related hepatocellular carcinoma (NASH-HCC)	The index positively correlated with BMI and HbA1c. The index was not significantly different among the patient groups.	The authors did not conclude directly about the index, only of these components.

**Table 8:** Characteristics and findings of studies that used ROM/BAP or BAP/ROM ratios.

Author, year [ref]	Study design/country	Population (age, sex)/sample size	Main outcome	Results	Conclusion
Kakita H et al., 2009 [116]	Case-control study/Japan	Preterm infants (31 weeks gestational age, both sexes)/22 infants with PVL 22 healthy	Association between OS and neonatal cystic periventricular leukomalacia (PVL)	ROM/BAP was higher in early cystic PVL than in late cystic PVL or controls. Postnatal duration until cyst identification displayed a negative correlation with ROM/BAP	Neonates experiencing more OS at birth show earlier onset of cystic PVL
Hussein MH et al., 2011 [99]	Observational Longitudinal/Japan	Children (1.2-14.4 y, both sexes)/43 patients	Relationship between OS and post-liver transplantation duration	ROM/BAP correlated positively with hepatic enzymes and negatively with post-liver transplantation duration.	The ROM/BAP can serve as an index of patients' laboratory results and oxidative status.
Chung DH et al., 2012 [117]	Intervention study/Korea	Adults (17-68 y, both sexes)/20 AR patients 20 NAR patients	To evaluate the susceptibility of patients with allergic rhinitis (AR) to OS compared with nonallergic rhinitis (NAR) patients	The ROM/BAP ratio was higher after surgery (nasal septoplasty) in the AR group than in the NAR group.	AR patients might be vulnerable to OS, due to their ROM/BAP imbalance.
Kaneko K et al., 2012 [118]	Intervention study/Japan	Children (0.14-5.38 y, both sexes)/19 patients with KD 7 patients with febrile illnesses (FI)	Association between OS and Kawasaki disease (KD) and if intravenous immunoglobulin therapy (IVIG) is a scavenger of ROS	Children with KD had low value of the ROM/BAP ratio after IVIG.	Seems that OS has a role in the pathogenesis of acute KD.
Kakita H et al., 2012 [119]	Retrospective case-control study/Japan	Newborns (37-41 wk gestational age, both sexes)/6 hypothermia 16 normothermia 15 control group	To evaluate OS in asphyxiated infants and to determinate if hypothermia treatment has an effect on OS	The ROM/BAP ratio values gradually increased after birth in hypothermia and normothermia cases. After 7 days, the ratio was higher in normothermia cases.	Hypothermia attenuated the OS in asphyxiated newborns.
Hussein MH et al., 2013 [120]	Observational Longitudinal/Japan	Children (2-15 y, both sexes)/16 IMD patients 10 BA patients	Assess the differences in OS between pediatric patients undergoing living-related liver transplantation due to inherited metabolic disease (IMD) or biliary atresia (BA)	The ROM/BAP ratio was higher in the IMD group.	Patients who receive living-related liver transplantation due to IMD are prone to higher ROM/BAP ratio.
Fukuda S et al., 2016 [121]	Experimental study/Japan	Adults (37 ± 9 y and 20 ± 0.5 y, both sexes)/12 females in acute stress condition 24 subjects in subacute fatigue condition 121 at-rest condition	Use dROMs and BAP to discriminate patients with chronic fatigue syndrome (CFS) from healthy volunteers experiencing acute and subacute fatigue, and at rest	The ROM/BAP ratio was higher after subacute fatigue. Resting condition produced higher ratio in patients with CFS than in healthy volunteers.	The markers might be useful for discriminate acute, subacute, and resting fatigue in healthy people and CFS patients.
Faienza MF et al., 2012 [113]	Cross-sectional/Italy	Children (11 ± 3 y, both sexes)/25 obese with MetS 30 obese 30 controls	Alterations in the oxidant/antioxidant status in obese children with and without metabolic syndrome (MetS)	Children without MetS had lower BAP/dROMs ratio than children with MetS. The ratio BAP/dROMs was higher in controls than no-MetS and MetS children.	The authors did not conclude directly about the index, but they infer that fat accumulation is involved in the pathogenesis of systemic OS.
Fukui T et al., 2015 [122]	Intervention study/Japan	Adults (58 ± 11 y, both sexes)/Patients with hyperuricemia 29 patients treated with febuxostat 14 patients treated with allopurinol	Effect of febuxostat compared with allopurinol on OS measured by BAP/dROMs ratio	No significant changes were observed in the BAP/dROMs ratio.	A regulatory mechanism may be counteracted changes in the OS balance caused by febuxostat administration.
Yamamoto K et al., 2015 [114]	Experimental pilot study/Japan	Adults (17-57 y, both sexes)/42 patients with active CD in treatment with anti-TNF-*α* antibodies	Effect of antitumor necrosis factor (TNF)-*α* treatment on OS in Crohn's disease (CD) patients. It used a modified BAP/dROMs ratio (m-BAP/dROMs ratio).	Negative correlation between m-BAP/dROMs ratio and CD activity index before and after treatment. The ratio did not differ between the patients before treatment.	Anti-TNF-*α* therapy decreases OS in patients with CD but does not modify the antioxidant production.
Hatanaka H et al., 2015 [123]	Cross-sectional/Japan	Older adults (81 ± 7 y, both sexes)/72 AD patients 27 with VaD 24 with MD 53 nondemented patients	OS differs in the pathophysiology and cognitive decline of Alzheimer's disease (AD), vascular dementia (VaD), and mixed Alzheimer's/vascular dementia (MD).	BAP/dROM ratios were lower in the AD and MD groups than control. The minimental state scores correlated with the ratio in the AD patients.	An imbalance in oxidant/antioxidant defenses may be involved in the pathophysiology of the AD and MD.
Nakagawa K et al., 2016 [115]	Cross-sectional/Japan	Adults (37 ± 3 y, women)/26 patients with unilateral EM 29 without EM	To evaluate OS in the environment of the follicular fluid (FF) of patients with endometrioma (EM)	The patients with a m-BAP/dROM ratio < 1.0 in the EM group was similar to the control group.	The oxidative/antioxidant potential in the FF of EM patients is similar to the controls.
Pesce M et al., 2018 [124]	Experimental study/Italy	Older adults (60-80 y, both sexes)/29 subjects in EG 23 in control group	Effect of memory training (MT) on plasma oxidant and antioxidant capacity	BAP/d-ROMs ratio improved in experimental group (EG). BAP/d-ROMs ratio was positively correlated with the measurement of memory.	MT was associated with the increase in resistance against OS at the plasma level.

ROS: reactive oxygen species.

**Table 9:** Characteristics and findings of studies that use the oxidative stress score (SS)^∗^ in Mexicans.

Author, year [ref]	Study design	Population (age, sex)/sample size	Main outcome	Results	Conclusion
Sánchez-Rodríguez MA. et al. 2005 [141]	Clinical trial	Older adults (67 ± 7 y, both sexes)/63 subjects with exercise and antioxidant diet 63 subjects with vitamins E 400 UI–C 500 mg/d 63 subjects with vitamins E 400 UI–C 1000 mg/d 80 controls	Effect of moderate physical exercise and antioxidant diet in comparison to oral vitamins C and E vs. severe OS	All groups with treatment after 1y had SS lower compared with pretest. Only 6 elderlies in the vitamin groups have severe OS after 1 y.	Moderate physical exercise and antioxidant diet have similar effect against severe OS than antioxidant vitamins in healthy elderlies.
Sánchez-Rodríguez MA. et al., 2006 [140]	Cross-sectional	Older adults (68 ± 7 y, both sexes)/121 subjects from Mexico City 85 subjects from a rural residence	To compare different OS marker vs. an integral score that considers oxidized biomolecules and the antioxidant (enzymatic and nonenzymatic) systems	Only SOD activity was similar in both groups. In urban residence, 42% of elderly had severe OS compared with 10% of the subjects of rural residence.	The proposed SS may be useful for assessing the severity of OS.
Sánchez-Rodríguez MA. et al., 2006 [138]	Cross-sectional	Older adults (68 ± 7 y, both sexes)/104 subjects from Mexico City 85 subjects from a rural area	Association between OS and cognitive impairment (CI) in elderly residents from rural and urban areas	Greater proportion of subjects with OS and CI in urban than in rural areas, being a risk factor. Higher OS and CI were observed in subjects >80 y of the urban area.	Elderly from urban area have more OS and greater risk of develop CI than inhabitants of rural communities.
Beristain-Pérez AS. et al., 2006 [142]	Cross-sectional	Older adults (67 ± 7 y, both sexes)/33 subjects with DM 40 subjects with AH 26 subjects with OA 63 healthy subjects	OS as risk factor for chronic degenerative diseases (CDD): type 2 diabetes mellitus (DM), arterial hypertension (AH), or osteoarthritis (OA) in older adults	OS was higher in DM and AH subjects. OS is a risk factor for CDD.	The OS is a risk factor for CDD, mainly for DM and AH subjects.
Sánchez-Rodríguez MA. et al., 2007 [143]	Case-control study	Older adults (68 ± 7 y, both sexes)/ 44 subjects with osteoporosis 50 healthy subjects	OS as an independent risk factor for osteoporosis in older adults	OS was higher in subjects with osteoporosis. OS is a significant risk factor for osteoporosis.	OS is a risk factor for osteoporosis.
Sánchez-Rodríguez MA. et al., 2010 [144]	Cross-sectional	Older adults (67 ± 1 y, both sexes)/63 with MetS 50 healthy	Association between the number of metabolic syndrome (MetS) components and OS.	The percentage of severe OS in subjects with MetS was higher. The risk for OS increases with the number of components.	MetS is linked to severe OxS, and the risk for OS increases with the number of MetS components.
Mendoza-Núñez VM. et al., 2011 [145]	Cross-sectional	Adults and older adults (both sexes)/56 healthy adults (47 ± 7 y) 60 DM adults (52 ± 6 y) 40 healthy older adults (67 ± 7 y) 72 DM older adults (68 ± 7 y)	Assess the additive effect of diabetes mellitus (DM) and aging on OS	DM is a risk factor for OS and a stronger factor in older subjects.	Aging and DM exerts an additive effect over the OS.
Sánchez-Rodríguez MA. et al., 2012 [146]	Cross-sectional	Adults (women) 94 premenopausal (45 ± 4 y) 93 postmenopausal (53 ± 3 y)	Influence of menopause as a risk factor for OS	SS was higher in postmenopause. There is an increase in the percentage of severe OS in postmenopausal women.	The decrease of estrogens in the postmenopause can cause OS, together with the symptoms of this period.
Rosado-Pérez J. et al., 2013 [147]	Quasiexperimental study	Older adults (60-74 y, both sexes)/43 subjects walking 31 subjects in tai chi 23 control subjects	Effect of the practice of tai chi and walking on OS	SS decrease in the tai chi and walking groups, but more evident in the tai chi group.	The practice of tai chi produces an antioxidant effect better than walking.
Sánchez-Rodríguez MA. et al., 2016 [148]	Randomized, double-blind, placebo-controlled trial	Adults (53 ± 1 y, women) 25 heathy with HT 25 healthy with placebo 25 MetS with HT 25 MetS with placebo	Effect of oral hormone therapy (HT) (1 mg/day of estradiol valerate plus 5 mg/10 day of medroxyprogesterone) on OS in postmenopausal women with metabolic syndrome (MetS).	SS decrease after 6 mo in the two groups with HT, more evident in the women with MetS. The proportion of women with OS decreases in both groups of women with HT.	HT improves OS associated with MetS in postmenopause.
Sánchez-Rodríguez MA. et al., 2017 [149]	Cross-sectional	Adults (40-59 y, women) 101 premenopausal 101 postmenopausal	Relationship between OS with psychological disturbances, low self-esteem, and low quality of life in the postmenopause	Women with low self-esteem and low quality of life had higher SS.	OS is increased in women with low self-esteem and low quality of life.
Mendoza-Núñez VM. et al., 2018 [150]	Quasiexperimental study	Older adults with MetS (60-74 y, both sexes) 60 subjects in TC 50 control group	Effect of tai chi (TC) exercise on OS in older adults with metabolic syndrome (MetS)	Decrease in the SS after TC training. The percentage of subjects with OS in the TC group decreases after 6 mo.	The TC exercise has an antioxidative effect in the older adults with MetS.
Rosado-Pérez J., et al., 2019 [151]	Exploratory study of a single group	Older adults (71 ± 6 y, both sexes)/12 subjects	Effect of the dried fruit powder of *Sechium edule* (chayote) on OS in older adults with metabolic syndrome (MetS).	SS decrease after 6 weeks of treatment.	The dry *Sechium edule* has an antioxidant effect in older adults with MetS.

^∗^All studies defined OS using the stress score (SS).

## Supplementary Materials

Supplementary Materials This is a file with additional references of thiol ratios and oxidative stress index (OSI) to complete the total articles reviewed and included in the descriptive analysis presented.

## References

[1] Gerschman R., Gilbert D. L., Nye S. W., Dwyer P., Fenn W. O. (1954). Oxygen poisoning and X-irradiation. A mechanism in common. *Science*.

[2] Harman D. (1956). Aging: a theory based on free radical and radiation chemistry. *Journal of Gerontology*.

[3] McCord J. M., Fridovich I. (1969). The utility of superoxide dismutase in studying free radical reactions. I. Radicals generated by the interaction of sulfite, dimethyl sulfoxide, and oxygen. *Journal of Biological Chemistry*.

[4] Sies H., Berndt C., Jones D. P. (2017). Oxidative stress. *Annual Review of Biochemistry*.

[5] Jones D. P. (2015). Redox theory of aging. *Redox Biology*.

[6] Finkel T., Holbrook N. J. (2000). Oxidants, oxidative stress and the biology of ageing. *Nature*.

[7] Dröge W. (2002). Free radicals in the physiological control of cell function. *Physiological Reviews*.

[8] Sies H. (2017). Hydrogen peroxide as a central redox signaling molecule in physiological oxidative stress: oxidative eustress. *Redox Biology*.

[9] Marrocco I., Altieri F., Peluso I. (2017). Measurement and clinical significance of biomarkers of oxidative stress in humans. *Oxidative Medicine and Cellular Longevity*.

[10] Armstrong D. (1998). *Free Radical and Antioxidant Protocols*.

[11] de Zwart L. L., Meerman J. H. N., Commandeur J. N. M., Vermeulen N. P. E. (1999). Biomarkers of free radical damage: Applications in experimental animals and in humans. *Free Radical Biology and Medicine*.

[12] Voss P., Siems W. (2009). Clinical oxidation parameters of aging. *Free Radical Research*.

[13] Forman H. J., Augusto O., Brigelius-Flohe R. (2015). Even free radicals should follow some rules: a guide to free radical research terminology and methodology. *Free Radical Biology & Medicine*.

[14] Lauterburg B. H., Smith C. V., Hughes H., Mitchell J. R. (1984). Biliary excretion of glutathione and glutathione disulfide in the rat. Regulation and response to oxidative stress. *The Journal of Clinical Investigation*.

[15] Kolettis P. N., Sharma R. K., Pasqualotto F. F., Nelson D., Thomas A. J., Agarwal A. (1999). Effect of seminal oxidative stress on fertility after vasectomy reversal. *Fertility and Sterility*.

[16] Pasqualotto F. F., Sharma R. K., Potts J. M., Nelson D. R., Thomas A. J., Agarwal A. (2000). Seminal oxidative stress in patients with chronic prostatitis. *Urology*.

[17] Pabón A., Carmona J., Burgos L. C., Blair S. (2003). Oxidative stress in patients with non-complicated malaria. *Clinical Biochemistry*.

[18] Harma M., Harma M., Erel O. (2003). Increased oxidative stress in patients with hydatidiform mole. *Swiss Medical Weekly*.

[19] Kocyigit A., Armutcu F., Gurel A., Ermis B. (2004). Alterations in plasma essential trace elements selenium, manganese, zinc, copper, and iron concentrations and the possible role of these elements on oxidative status in patients with childhood asthma. *Biological Trace Element Research*.

[20] Agha-Hosseini F., Mirzaii-Dizgah I., Farmanbar N., Abdollahi M. (2012). Oxidative stress status and DNA damage in saliva of human subjects with oral lichen planus and oral squamous cell carcinoma. *Journal of Oral Pathology & Medicine*.

[21] Venturini D., Simão A. N. C., Urbano M. R., Dichi I. (2015). Effects of extra virgin olive oil and fish oil on lipid profile and oxidative stress in patients with metabolic syndrome. *Nutrition*.

[22] Sales de Almeida J. P., Liberatti L. S., Nascimento Barros F. E. (2016). Profile of oxidative stress markers is dependent on vitamin D levels in patients with chronic hepatitis C. *Nutrition*.

[23] Costa N. T., Veiga Iriyoda T. M., Kallaur A. P. (2016). Influence of Insulin Resistance and TNF-*α* on the Inflammatory Process, Oxidative Stress, and Disease Activity in Patients with Rheumatoid Arthritis. *Oxidative Medicine and Cellular Longevity*.

[24] Becatti M., Fucci R., Mannucci A. (2018). A biochemical approach to detect oxidative stress in infertile women undergoing assisted reproductive technology procedures. *International Journal of Molecular Sciences*.

[25] Uysal P., Avcil S., Abas B. İ., Yenisey Ç. (2016). Evaluation of oxidant-antioxidant balance in children with atopic dermatitis: a case-control study. *American Journal of Clinical Dermatology*.

[26] Tošić-Pajić J., Šeklić D., Radenković J. (2017). Augmented oxidative stress in infertile women with persistent chlamydial infection. *Reproductive Biology*.

[27] Curello S., Ceconi C., Cargnoni A., Cornacchiari A., Ferrari R., Albertini A. (1987). Improved procedure for determining glutathione in plasma as an index of myocardial oxidative stress. *Clinical Chemistry*.

[28] Giustarini D., Tsikas D., Colombo G. (2016). Pitfalls in the analysis of the physiological antioxidant glutathione (GSH) and its disulfide (GSSG) in biological samples: an elephant in the room. *Journal of Chromatography B*.

[29] Jones D. P., Liang Y. (2009). Measuring the poise of thiol/disulfide couples in vivo. *Free Radical Biology & Medicine*.

[30] Huang Y. S., Wang L. X., Sun L. (2009). Elevated peroxidative glutathione redox status in atherosclerotic patients with increased thickness of carotid intima media. *Chinese Medical Journal*.

[31] Németh I., Boda D. (1994). Blood glutathione redox ratio as a parameter of oxidative stress in premature infants with IRDS. *Free Radical Biology & Medicine*.

[32] Papp A., Németh I., Karg E., Papp E. (1999). Glutathione status in retinopathy of prematurity. *Free Radical Biology & Medicine*.

[33] Paolisso G., Gambardella A., Tagliamonte M. R. (1996). Does free fatty acid infusion impair insulin action also through an increase in oxidative stress?. *Journal of Clinical Endocrinology and Metabolism*.

[34] Abramson J. L., Hooper W. C., Jones D. P. (2005). Association between novel oxidative stress markers and C-reactive protein among adults without clinical coronary heart disease. *Atherosclerosis*.

[35] Gherghel D., Griffiths H. R., Hilton E. J., Cunliffe I. A., Hosking S. L. (2005). Systemic reduction in glutathione levels occurs in patients with primary open-angle glaucoma. *Investigative Ophthalmology & Visual Science*.

[36] Yeh C. C., Hou M. F., Wu S. H. (2006). A study of glutathione status in the blood and tissues of patients with breast cancer. *Cell Biochemistry and Function*.

[37] Ashfaq S., Abramson J. L., Jones D. P. (2006). The relationship between plasma levels of oxidized and reduced thiols and early atherosclerosis in healthy adults. *Journal of the American College of Cardiology*.

[38] Nikolaidis M., Paschalis V., Giakas G. (2007). Decreased blood oxidative stress after repeated muscle-damaging exercise. *Medicine & Science in Sports & Exercise*.

[39] Harzallah O., Kerkeni A., Baati T., Mahjoub S. (2008). Oxidative stress: Correlation with Behçet's disease duration, activity and severity. *European Journal of Internal Medicine*.

[40] Youssef H., Groussard C., Pincemail J. (2009). Exercise-induced oxidative stress in overweight adolescent girls: roles of basal insulin resistance and inflammation and oxygen overconsumption. *International Journal of Obesity*.

[41] Tsai S. M., Lin S. K., Lee K. T. (2009). Evaluation of redox statuses in patients with hepatitis B virus-associated hepatocellular carcinoma. *Annals of Clinical Biochemistry*.

[42] Kleinsorge E. C., Erben M., Galan M. G., Barison C., Gonsebatt M. E., Simoniello M. F. (2011). Assessment of oxidative status and genotoxicity in photocopier operators: a pilot study. *Biomarkers*.

[43] Patel R. S., al Mheid I., Morris A. A. (2011). Oxidative stress is associated with impaired arterial elasticity. *Atherosclerosis*.

[44] Zepeda R. J., Castillo R., Rodrigo R. (2012). Effect of carvedilol and nebivolol on oxidative stress-related parameters and endothelial function in patients with essential hypertension. *Basic & Clinical Pharmacology & Toxicology*.

[45] Llorente-Cantarero F. J., Gil-Campos M., Benitez-Sillero J. . D., Muñoz-Villanueva M. C., Tasset I., Pérez-Navero J. L. (2013). Profile of oxidant and antioxidant activity in prepubertal children related to age, gender, exercise, and fitness. *Applied Physiology, Nutrition, and Metabolism*.

[46] Ntalapascha M., Makris D., Kyparos A. (2013). Oxidative stress in patients with obstructive sleep apnea syndrome. *Sleep & Breathing*.

[47] Shah D., Sah S., Wanchu A., Wu M. X., Bhatnagar A. (2013). Altered redox state and apoptosis in the pathogenesis of systemic lupus erythematosus. *Immunobiology*.

[48] Victor V. M., Rocha M., Bañuls C., Rovira-Llopis S., Gómez M., Hernández-Mijares A. (2014). Mitochondrial impairment and oxidative stress in leukocytes after testosterone administration to female‐to‐male transsexuals. *The Journal of Sexual Medicine*.

[49] Enns G. M., Moore T., le A. (2014). Degree of glutathione deficiency and redox imbalance depend on subtype of mitochondrial disease and clinical status. *PLoS One*.

[50] Schmitt B., Vicenzi M., Garrel C., Denis F. M. (2015). Effects of N-acetylcysteine, oral glutathione (GSH) and a novel sublingual form of GSH on oxidative stress markers: A comparative crossover study.. *Redox Biology*.

[51] Karimi R., Vacchi-Suzzi C., Meliker J. R. (2016). Mercury exposure and a shift toward oxidative stress in avid seafood consumers. *Environmental Research*.

[52] Atkin M., Laight D., Cummings M. H. (2016). The effects of garlic extract upon endothelial function, vascular inflammation, oxidative stress and insulin resistance in adults with type 2 diabetes at high cardiovascular risk. A pilot double blind randomized placebo controlled trial. *Journal of Diabetes and its Complications*.

[53] Galicia-Moreno M., Rosique-Oramas D., Medina-Avila Z. (2016). Behavior of oxidative stress markers in alcoholic liver cirrhosis patients. *Oxidative Medicine and Cellular Longevity*.

[54] Moreno-Solís G., dela Torre-Aguilar M. J., Torres-Borrego J. (2017). Oxidative stress and inflamatory plasma biomarkers in respiratory syncytial virus bronchiolitis. *The Clinical Respiratory Journal*.

[55] Vacchi-Suzzi C., Viens L., Harrington J. M., Levine K., Karimi R., Meliker J. R. (2018). Low levels of lead and glutathione markers of redox status in human blood. *Environmental Geochemistry and Health*.

[56] Bagan J., Sáez G. T., Tormos M. C. (2014). Oxidative stress in bisphosphonate-related osteonecrosis of the jaws. *Journal of Oral Pathology & Medicine*.

[57] Arana C., Moreno-Fernández A. M., Gómez-Moreno G. (2017). Incremento de los parametros de estres oxidativo salival en pacientes con diabetes tipo 2: relacion con la enfermedad periodontal. *Endocrinología, Diabetes y Nutrición (English ed.)*.

[58] Annuk M., Zilmer M., Lind L., Linde T., Fellström B. (2001). Oxidative stress and endothelial function in chronic renal failure. *Journal of the American Society of Nephrology*.

[59] Sáez G. T., Tormos C., Giner V. (2004). Factors related to the impact of antihypertensive treatment in antioxidant activities and oxidative stress by-products in human hypertension. *American Journal of Hypertension*.

[60] Skalicky J., Muzakova V., Kandar R., Meloun M., Rousar T., Palicka V. (2008). Evaluation of oxidative stress and inflammation in obese adults with metabolic syndrome. *Clinical Chemistry and Laboratory Medicine*.

[61] Lind L., Andersson J., Rönn M. (2008). Brachial artery intima-media thickness and echogenicity in relation to lipids and markers of oxidative stress in elderly subjects: the Prospective Investigation of the Vasculature in Uppsala Seniors (PIVUS) Study. *Lipids*.

[62] Mercken E. M., Gosker H. R., Rutten E. P. (2009). Systemic and pulmonary oxidative stress after single-leg exercise in COPD. *Chest*.

[63] Real J. T., Martínez-Hervás S., Tormos M. C. (2010). Increased oxidative stress levels and normal antioxidant enzyme activity in circulating mononuclear cells from patients of familial hypercholesterolemia. *Metabolism*.

[64] Rusanova I., Escames G., Cossio G. (2010). Oxidative stress status, clinical outcome, and *β*‐globin gene cluster haplotypes in pediatric patients with sickle cell disease. *European Journal of Haematology*.

[65] Petrillo S., Piemonte F., Pastore A. (2013). Glutathione imbalance in patients with X-linked adrenoleukodystrophy. *Molecular Genetics and Metabolism*.

[66] De Tursi Ríspoli L., Vázquez Tarragón A., Vázquez Prado A. (2013). Relationship of oxidative stress and weight loss achieved in morbid obese patients by means of bariatric surgery using the duodenal switch technique. *Nutrición Hospitalaria*.

[67] Blasco H., Garcon G., Patin F. (2017). Panel of oxidative stress and inflammatory biomarkers in ALS: a pilot study. *The Canadian Journal of Neurological Sciences*.

[68] Bellanti F., Romano A. D., Lo Buglio A. (2018). Oxidative stress is increased in sarcopenia and associated with cardiovascular disease risk in sarcopenic obesity. *Maturitas*.

[69] Khazim K., Giustarini D., Rossi R. (2013). Glutathione redox potential is low and glutathionylated and cysteinylated hemoglobin levels are elevated in maintenance hemodialysis patients. *Translational Research*.

[70] Erel O., Neselioglu S. (2014). A novel and automated assay for thiol/disulphide homeostasis. *Clinical Biochemistry*.

[71] Baba S. P., Bhatnagar A. (2018). Role of thiols in oxidative stress. *Current Opinion in Toxicology*.

[72] Alsalman A. R. S., Almashhedy L. A., Hadwan M. H. (2018). Effect of oral zinc supplementation on the thiol oxido-reductive index and thiol-related enzymes in seminal plasma and spermatozoa of Iraqi asthenospermic patients. *Biological Trace Element Research*.

[73] Elbay A., Ozer O. F., Altinisik M. (2017). A novel tool reflecting the role of oxidative stress in the cataracts: thiol/disulfide homeostasis. *Scandinavian Journal of Clinical and Laboratory Investigation*.

[74] Erel O. (2004). A novel automated direct measurement method for total antioxidant capacity using a new generation, more stable ABTS radical cation. *Clinical Biochemistry*.

[75] Erel O. (2005). A new automated colorimetric method for measuring total oxidant status. *Clinical Biochemistry*.

[76] Erel O. (2004). A novel automated method to measure total antioxidant response against potent free radical reactions. *Clinical Biochemistry*.

[77] Tatzber F., Griebenow S., Wonisch W., Winkler R. (2003). Dual method for the determination of peroxidase activity and total peroxides- iodide leads to a significant increase of peroxidase activity in human sera. *Analytical Biochemistry*.

[78] Zelzer S., Tatzber F., Herrmann M. (2018). Work intensity, low-grade inflammation, and oxidative status: a comparison between office and slaughterhouse workers. *Oxidative Medicine and Cellular Longevity*.

[79] Verit F. F., Verit A., Kocyigit A., Ciftci H., Celik H., Koksal M. (2006). No increase in sperm DNA damage and seminal oxidative stress in patients with idiopathic infertility. *Archives of Gynecology and Obstetrics*.

[80] Yazar H., Halis F., Nasir Y., Guzel D., Akdogan M., Gokce A. (2017). Effect of the oxidant-antioxidant system in seminal plasma on varicocele and idiopathic infertility in male humans. *Clinical Laboratory*.

[81] Ciftci H., Verit A., Savas M., Yeni E., Erel O. (2009). Effects of *N*-acetylcysteine on semen parameters and oxidative/antioxidant status. *Urology*.

[82] Ciftci H., Verit A., Yeni E., Savas M. (2008). Decreased oxidative stress index of urine in patients with urinary tract infection. *Urologia Internationalis*.

[83] Göknar N., Oktem F., Arı E., Demir A. D., Torun E. (2014). Is oxidative stress related to childhood urolithiasis?. *Pediatric Nephrology*.

[84] Aycicek A., Iscan A., Erel O., Akcali M., Ocak A. R. (2007). Oxidant and antioxidant parameters in the treatment of meningitis. *Pediatric Neurology*.

[85] Vural M., Camuzcuoglu H., Toy H., Aksoy N. (2010). Amniotic fluid prolidase activity and oxidative status in neural tube defects. *Fetal Diagnosis and Therapy*.

[86] Soydinc H. E., Sak M. E., Evliyaoglu O. (2013). Prolidase, matrix metalloproteinases 1 and 13 activity, oxidative-antioxidative status as a marker of preterm premature rupture of membranes and chorioamnionitis in maternal vaginal washing fluids. *International Journal of Medical Sciences*.

[87] Esen C., Alkan B. A., Kırnap M., Akgül O., Işıkoğlu S., Erel O. (2012). The effects of chronic periodontitis and rheumatoid arthritis on serum and gingival crevicular fluid total antioxidant/oxidant status and oxidative stress index. *Journal of Periodontology*.

[88] Bostanci V., Toker H., Senel S., Ozdemir H., Aydin H. (2014). Effect of chronic periodontitis on serum and gingival crevicular fluid oxidant and antioxidant status in patients with familial Mediterranean fever before and after periodontal treatment. *Journal of Periodontology*.

[89] Dursun E., Akalin F. A., Genc T., Cinar N., Erel O., Yildiz B. O. (2016). Oxidative stress and periodontal disease in obesity. *Medicine*.

[90] Cağlayan F., Miloglu O., Altun O., Erel O., Yilmaz A. B. (2008). Oxidative stress and myeloperoxidase levels in saliva of patients with recurrent aphthous stomatitis. *Oral Diseases*.

[91] Baltacıoğlu E., Yuva P., Aydın G. (2014). Lipid peroxidation levels and total oxidant/antioxidant status in serum and saliva from patients with chronic and aggressive periodontitis. Oxidative stress index: a new biomarker for periodontal disease?. *Journal of Periodontology*.

[92] Knaś M., Maciejczyk M., Sawicka K. (2016). Impact of morbid obesity and bariatric surgery on antioxidant/oxidant balance of the unstimulated and stimulated human saliva. *Journal of Oral Pathology & Medicine*.

[93] Torumtay G., Kırzıoğlu F. Y., Öztürk Tonguç M., Kale B., Calapoğlu M., Orhan H. (2016). Effects of periodontal treatment on inflammation and oxidative stress markers in patients with metabolic syndrome. *Journal of Periodontal Research*.

[94] Buczko P., Knaś M., Grycz M., Szarmach I., Zalewska A. (2017). Orthodontic treatment modifies the oxidant-antioxidant balance in saliva of clinically healthy subjects. *Advances in Medical Sciences*.

[95] Tripathi V., Singh S. T., Sharma V., Verma A., Singh C. D., Gill J. S. (2018). Assessment of lipid peroxidation levels and total antioxidant status in chronic and aggressive periodontitis patients: an in vivo study. *The Journal of Contemporary Dental Practice*.

[96] Beyazyıldız E., Çankaya A. B., Beyazyıldız Ö. (2014). Disturbed oxidant/antioxidant balance in aqueous humour of patients with exfoliation syndrome. *Japanese Journal of Ophthalmology*.

[97] Altinisik M., Koytak A., Elbay A., Toklu E., Sezer T., Kocyigit A. (2017). Oxidant-antioxidant balance in the aqueous humor of patients with retinal vein occlusion. *Seminars in Ophthalmology*.

[98] Vassalle C., Pratali L., Boni C., Mercuri A., Ndreu R. (2008). An oxidative stress score as a combined measure of the pro-oxidant and anti- oxidant counterparts in patients with coronary artery disease. *Clinical Biochemistry*.

[99] Hussein M. H., Hashimoto T., Daoud G. A.-H. (2011). Oxidative stress after living related liver transplantation subsides with time in pediatric patients. *Pediatric Surgery International*.

[100] Buico A., Cassino C., Ravera M., Betta P. G., Osella D. (2009). Oxidative stress and total antioxidant capacity in human plasma. *Redox Report*.

[101] Trotti R., Carratelli M., Barbieri M. (2001). Oxidative stress and a thrombophilic condition in alcoholics without severe liver disease. *Haematologica*.

[102] Vassalle C., Armstrong D. (2008). An easy and reliable automated method to estimate oxidative stress in the clinical setting. *Advanced Protocols in Oxidative Stress I. Methods in Molecular Biology*.

[103] Vassalle C., Maffei S., Ndreu R., Mercuri A. (2009). Age-related oxidative stress modulation by smoking habit and obesity. *Clinical Biochemistry*.

[104] Vassalle C., Novembrino C., Maffei S. (2011). Determinants of oxidative stress related to gender: relevance of age and smoking habit. *Clinical Chemistry and Laboratory Medicine*.

[105] Tamaki N., Tomofuji T., Ekuni D., Yamanaka R., Morita M. (2011). Periodontal treatment decreases plasma oxidized LDL level and oxidative stress. *Clinical Oral Investigations*.

[106] Vassalle C., Bianchi S., Battaglia D., Landi P., Bianchi F., Carpeggiani C. (2012). Elevated levels of oxidative stress as a prognostic predictor of major adverse cardiovascular events in patients with coronary artery disease. *Journal of Atherosclerosis and Thrombosis*.

[107] Vassalle C., Sciarrino R., Bianchi S., Battaglia D., Mercuri A., Maffei S. (2012). Sex-related differences in association of oxidative stress status with coronary artery disease. *Fertility and Sterility*.

[108] Capaccio P., Pignataro L., Gaini L. M. (2012). Unbalanced oxidative status in idiopathic sudden sensorineural hearing loss. *European Archives of Otorhinolaryngology*.

[109] Terao M., Takaki A., Maruyama T. (2015). Serum oxidative/anti-oxidative stress balance is dysregulated in potentially pulmonary hypertensive patients with liver cirrhosis: a case control study. *Internal Medicine*.

[110] Uchida D., Takaki A., Ishikawa H. (2016). Oxidative stress balance is dysregulated and represents an additional target for treating cholangiocarcinoma. *Free Radical Research*.

[111] Mizuno H., Ekuni D., Maruyama T. (2017). The effects of non-surgical periodontal treatment on glycemic control, oxidative stress balance and quality of life in patients with type 2 diabetes: a randomized clinical trial. *PLoS One*.

[112] Shimomura Y., Takaki A., Wada N. (2017). The serum oxidative/anti-oxidative stress balance becomes dysregulated in patients with non-alcoholic steatohepatitis associated with hepatocellular carcinoma. *Internal Medicine*.

[113] Faienza M. F., Francavilla R., Goffredo R. (2012). Oxidative stress in obesity and metabolic syndrome in children and adolescents. *Hormone Research in Pædiatrics*.

[114] Yamamoto K., Chiba T., Matsumoto T. (2015). Effect of tumor necrosis factor-*α* antagonists on oxidative stress in patients with Crohn’s disease. *World Journal of Gastroenterology*.

[115] Nakagawa K., Hisano M., Sugiyama R., Yamaguchi K. (2016). Measurement of oxidative stress in the follicular fluid of infertility patients with an endometrioma. *Archives of Gynecology and Obstetrics*.

[116] Kakita H., Hussein M. H., Yamada Y. (2009). High postnatal oxidative stress in neonatal cystic periventricular leukomalacia. *Brain & Development*.

[117] Chung D. H., Lee K. H., Kim S. W., Shin S. Y., Cho J. S. (2012). Comparison of pre- and post-operative stress levels in patients with allergic rhinitis and non-allergic rhinitis. *European Archives of Otorhinolaryngology*.

[118] Kaneko K., Takahashi M., Yoshimura K. (2012). Intravenous immunoglobulin counteracts oxidative stress in Kawasaki disease. *Pediatric Cardiology*.

[119] Kakita H., Hussein M. H., Kato S. (2012). Hypothermia attenuates the severity of oxidative stress development in asphyxiated newborns. *Journal of Critical Care*.

[120] Hussein M. H., Hashimoto T., Suzuki T. (2013). Children undergoing liver transplantation for treatment of inherited metabolic diseases are prone to higher oxidative stress, complement activity and transforming growth factor-*β*1. *Annals of Transplantation*.

[121] Fukuda S., Nojima J., Motoki Y. (2016). A potential biomarker for fatigue: oxidative stress and anti-oxidative activity. *Biological Psychology*.

[122] Fukui T., Maruyama M., Yamauchi K., Yoshitaka S., Yasuda T., Abe Y. (2015). Effects of febuxostat on oxidative stress. *Clinical Therapeutics*.

[123] Hatanaka H., Hanyu H., Fukasawa R. (2015). Differences in peripheral oxidative stress markers in Alzheimer’s disease, vascular dementia and mixed dementia patients. *Geriatrics Gerontology International*.

[124] Pesce M., Tatangelo R., La Fratta I. (2018). Aging-related oxidative stress: positive effect of memory training. *Neuroscience*.

[125] Jansen E., Ruskovska T. (2015). Serum biomarkers of (anti)oxidant status for epidemiological studies. *International Journal of Molecular Sciences*.

[126] Cutler R. G. (2005). Oxidative stress profiling: Part I. Its potential importance in the optimization of human health. *Annals of the New York Academy of Sciences*.

[127] Cutler R. G., Plummer J., Chowdhury K., Heward C. (2005). Oxidative stress profiling: Part II. Theory, technology, and practice. *Annals of the New York Academy of Sciences*.

[128] Veglia F., Cighetti G., De Franceschi M. (2006). Age- and gender-related oxidative status determined in healthy subjects by means of OXY-SCORE, a potential new comprehensive index. *Biomarkers*.

[129] Veglia F., Werba J. P., Tremoli E. (2009). Assessment of oxidative stress in coronary artery bypass surgery: comparison between the global index OXY-SCORE and individual biomarkers. *Biomarkers*.

[130] Cavalca V., Veglia F., Squellerio I. (2009). Glutathione, vitamin E and oxidative stress in coronary artery disease: relevance of age and gender. *European Journal of Clinical Investigation*.

[131] Ruiz-Hurtado G., Condezo-Hoyos L., Pulido-Olmo H. (2014). Development of albuminuria and enhancement of oxidative stress during chronic renin-angiotensin system suppression. *Journal of Hypertension*.

[132] Amstad P., Peskin A., Shah G. (1991). The balance between copper-zinc superoxide dismutase and catalase affects the sensitivity of mouse epidermal cells to oxidative stress. *Biochemistry*.

[133] Remacle J., Lambert D., Raes M., Pigeolet E., Michiels C., Toussaint O. (1992). Importance of various antioxidant enzymes for cell stability. Confrontation between theoretical and experimental data. *Biochemical Journal*.

[134] Michiels C., Raes M., Toussaint O., Remacle J. (1994). Importance of SE-glutathione peroxidase, catalase, and CU/ZN-SOD for cell survival against oxidative stress. *Free Radical Biology & Medicine*.

[135] Halliwell B. (2006). Reactive species and antioxidants. Redox biology is a fundamental theme of aerobic life. *Plant Physiology*.

[136] Cristiano F., de Haan J. B., Iannello R. C., Kola I. (1995). Changes in the levels of enzymes which modulate the antioxidant balance occur during aging and correlate with cellular damage. *Mechanisms of Ageing and Development*.

[137] Sánchez-Rodríguez M. A., Santiago-Osorio E., Vargas L. A., Mendoza-Núñez V. M. (2004). Propuesta de un constructo para evaluar integralmente el estrés oxidativo. *Bioquimia*.

[138] Sánchez-Rodríguez M. A., Santiago E., Arronte-Rosales A., Vargas-Guadarrama L. A., Mendoza-Núñez V. M. (2006). Relationship between oxidative stress and cognitive impairment in the elderly of rural vs. urban communities. *Life Sciences*.

[139] Lushchak V. I. (2014). Free radicals, reactive oxygen species, oxidative stress and its classification. *Chemico-Biological Interactions*.

[140] Sanchez-Rodriguez M., Ruiz-Ramos M., Mendoza-Nuñez V. M. (2006). Proposal of a construct to measure severity of oxidative stress. *Free Radical Biology & Medicine*.

[141] Sánchez-Rodríguez M., Ruiz-Ramos M., Mendoza-Núñez V. M. (2005). Exercise plus antioxidantdiet vs. vitamins C and E supplementation against severe oxidative stress in Mexican elderly. *Free Radical Biology & Medicine*.

[142] Beristain-Pérez A. S., Sánchez-Rodríguez M. A., Ruiz-Ramos M., Mendoza-Núñez V. M. (2006). Estrés oxidativo como factor de riesgo para el desarrollo de diabetes mellitus, osteoartritis o hipertensión arterial en adultos mayores. *Bioquimia*.

[143] Sánchez-Rodríguez M. A., Ruiz-Ramos M., Correa-Muñoz E., Mendoza-Núñez V. M. (2007). Oxidative stress as a risk factor for osteoporosis in elderly Mexicans as characterized by antioxidant enzymes. *BMC Musculoskeletal Disorders*.

[144] Sánchez-Rodríguez M. A., Martínez-Cruz M., Correa-Muñoz E., Mendoza-Núñez V. M. (2010). Relationship between metabolic syndrome components and oxidative stress in elderly community-dwelling Mexicans. *Annals of Nutrition & Metabolism*.

[145] Mendoza-Núñez V. M., Rosado-Pérez J., Santiago-Osorio E., Ortiz R., Sánchez-Rodríguez M. A., Galván-Duarte R. E. (2011). Aging linked to type 2 diabetes increases oxidative stress and chronic inflammation. *Rejuvenation Research*.

[146] Sánchez-Rodríguez M. A., Zacarías-Flores M., Arronte-Rosales A., Correa-Muñoz E., Mendoza-Núñez V. M. (2012). Menopause as risk factor for oxidative stress. *Menopause*.

[147] Rosado-Pérez J., Ortiz R., Santiago-Osorio E., Mendoza-Núñez V. M. (2013). Effect of tai chi versus walking on oxidative stress in Mexican older adults. *Oxidative Medicine and Cellular Longevity*.

[148] Sánchez-Rodríguez M. A., Zacarías-Flores M., Castrejón-Delgado L., Ruiz-Rodríguez A. K., Mendoza-Núñez V. M. (2016). Effects of hormone therapy on oxidative stress in postmenopausal women with metabolic syndrome. *International Journal of Molecular Sciences*.

[149] Sánchez-Rodríguez M. A., Castrejón-Delgado L., Zacarías-Flores M., Arronte-Rosales A., Mendoza-Núñez V. M. (2017). Quality of life among post-menopausal women due to oxidative stress boosted by dysthymia and anxiety. *BMC Women’s Health*.

[150] Mendoza-Núñez V. M., Arista-Ugalde T. L., Rosado-Pérez J., Ruiz-Ramos M., Santiago-Osorio E. (2018). Hypoglycemic and antioxidant effect of tai chi exercise training in older adults with metabolic syndrome. *Clinical Interventions in Aging*.

[151] Rosado-Pérez J., Aguiñiga-Sánchez I., Santiago-Osorio E., Mendoza-Núñez V. M. (2019). Effect of *Sechium edule* var. *nigrum spinosum* (Chayote) on oxidative stress and pro-inflammatory markers in older adults with metabolic syndrome: an exploratory study. *Antioxidants*.

[152] Goodman M., Bostick R. M., Dash C., Flanders W. D., Mandel J. S. (2007). Hypothesis: oxidative stress score as a combined measure of pro-oxidant and antioxidant exposures. *Annals of Epidemiology*.

[153] Geybels M. S., Verhage B. A. J., van Schooten F. J., van den Brandt P. A. (2012). Measures of combined antioxidant and pro-oxidant exposures and risk of overall and advanced stage prostate cancer. *Annals of Epidemiology*.

[154] Lakkur S., Goodman M., Bostick R. M. (2014). Oxidative balance score and risk for incident prostate cancer in a prospective U.S. cohort study. *Annals of Epidemiology*.

[155] Kong S. Y., Goodman M., Judd S., Bostick R. M., Flanders W. D., McClellan W. (2015). Oxidative balance score as predictor of all-cause, cancer, and noncancer mortality in a biracial US cohort. *Annals of Epidemiology*.

[156] Ilori T. O., Sun Ro Y., Kong S. Y. (2015). Oxidative balance score and chronic kidney disease. *American Journal of Nephrology*.

[157] Annor F. B., Goodman M., Okosun I. S. (2015). Oxidative stress, oxidative balance score, and hypertension among a racially diverse population. *Journal of the American Society of Hypertension*.

[158] Ilori T. O., Wang X., Huang M. (2017). Oxidative balance score and the risk of end-stage renal disease and cardiovascular disease. *American Journal of Nephrology*.

[159] Lakkur S., Bostick R. M., Roblin D. (2014). Oxidative balance score and oxidative stress biomarkers in a study of Whites, African Americans, and African immigrants. *Biomarkers*.

[160] Cho A. R., Kwon Y. J., Lim H. J. (2018). Oxidative balance score and serum *γ*-glutamyltransferase level among Korean adults: a nationwide population-based study. *European Journal of Nutrition*.

[161] Kong S. Y. J., Bostick R. M., Flanders W. D. (2014). Oxidative balance score, colorectal adenoma, and markers of oxidative stress and inflammation. *Cancer Epidemiology, Biomarkers & Prevention*.

[162] Lakkur S., Judd S., Bostick R. M. (2015). Oxidative stress, inflammation, and markers of cardiovascular health. *Atherosclerosis*.

[163] Labadie J., Goodman M., Thyagarajan B. (2013). Associations of oxidative balance-related exposures with incident, sporadic colorectal adenoma according to antioxidant enzyme genotypes. *Annals of Epidemiology*.

[164] Slattery M. L., Pellatt D. F., Mullany L. E., Wolff R. K. (2015). Differential gene expression in colon tissue associated with diet, lifestyle, and related oxidative stress. *PLoS One*.

[165] Lee H. S., Park T. (2017). Pathway-driven approaches of interaction between oxidative balance and genetic polymorphism on metabolic syndrome. *Oxidative Medicine and Cellular Longevity*.

[166] Wang T., Goodman M., Sun Y. V., Thyagarajan B., Gross M., Bostick R. M. (2017). DNA base excision repair genetic risk scores, oxidative balance, and incident, sporadic colorectal adenoma. *Molecular Carcinogenesis*.

[167] Charles L. E., Burchfiel C. M., Violanti J. M. (2008). Adiposity measures and oxidative stress among police officers. *Obesity*.

[168] Sanayama Y., Nagasaka H., Takayanagi M. (2011). Experimental evidence that phenylalanine is strongly associated to oxidative stress in adolescents and adults with phenylketonuria. *Molecular Genetics and Metabolism*.

[169] Kolesnikova L. I., Semyonova N. V., Grebenkina L. A., Darenskaya M. A., Suturina L. V., Gnusina S. V. (2014). Integral indicator of oxidative stress in human blood. *Bulletin of Experimental Biology and Medicine*.

[170] Acharya J. D., Pande A. J., Joshi S. M., Yajnik C. S., Ghaskadbi S. S. (2014). Treatment of hyperglycaemia in newly diagnosed diabetic patients is associated with a reduction in oxidative stress and improvement in *β*‐cell function. *Diabetes/Metabolism Research and Reviews*.

[171] Rael L. T., Bar-Or R., Aumann R. M., Slone D. S., Mains C. W., Bar-Or D. (2007). Oxidation-reduction potential and paraoxonase-arylesterase activity in trauma patients. *Biochemical and Biophysical Research Communications*.

[172] Alamdari D. H., Paletas K., Pegiou T., Sarigianni M., Befani C., Koliakos G. (2007). A novel assay for the evaluation of the prooxidant-antioxidant balance, before and after antioxidant vitamin administration in type II diabetes patients. *Clinical Biochemistry*.

