# Supplementary material for: Oxidative Stress Indexes for Diagnosis of Health or Disease in Humans
Source: Oxid Med Cell Longev. 2019 Nov 25;2019:4128152. doi: 10.1155/2019/4128152 (PMC6899293; doi:10.1155/2019/4128152)
Supplement: Supplementary Materials — This is a file with additional references of thiol ratios and oxidative stress index (OSI) to complete the total articles reviewed and included in the descriptive analysis presented. [file 4128152.f1.docx]

**Additional References**

**Thiol ratios**

1. H. U. Yuvaci, N. Akdemir, M. S. Bostanci, et al., “Evaluation of the level of thiol-disulphide homeostasis in patients with mild and severe preeclampsia,” *Pregnancy Hypertension*, vol. 6, no. 4, pp. 394–399, 2016.
2. K. Erkenekli, C. Y. Sanhal, A. Yucel, C. K. Bicer, O. Erel and D. Uygur, “Thiol/disulfide homeostasis in patients with idiopathic recurrent pregnancy loss assessed by a novel assay: Report of a preliminary study,” *The Journal of Obstetrics and Gynaecology Research*, vol. 42, no. 2, pp. 136–141, 2016.
3. E. Karaman, O. Çetin, B. Boza, et al., “Maternal serum thiol/disulfide homeostasis in pregnancies complicated by neural tube defects: report of a preliminary study”, *The Journal of Maternal-Fetal and Neonatal Medicine*, vol. 30, no. 15, pp. 1803–1808, 2017.
4. S. Ozler, E. Oztas, O. Erel, et al., “Impact of gestational diabetes mellitus and maternal obesity on cord blood dynamic thiol/disulfide homeostasis.” *Fetal and Pediatric Pathology*, vol. 36, no. 1, pp. 8–15, 2017.
5. H. Akkaya, G. Uysal, B. Büke, G. Gök, Ö. Erel and Ç. Karakükçü, “Evaluation of thiol/disulphide homeostasis as a novel predictor testing tool of early pregnancy viability,” *Taiwanese Journal of Obstetrics & Gynecology*, vol. 57, no. 3, pp. 427–431, 2018.
6. C. Y. Sanhal, K. Daglar, O. Kara, et al., “An alternative method for measuring oxidative stress in intrahepatic cholestasis of pregnancy: thiol/disulphide homeostasis,” *The Journal of Maternal-Fetal and Neonatal Medicine,* vol. 31, no. 11, pp. 1477–1482, 2018.
7. C. Katar-Yildirim, A. Tokmak, C. Yildirim, O. Erel and A. T. Caglar, “Investigation of serum thiol/disulphide homeostasis in patients with abortus imminens,” *The Journal of Maternal-Fetal and Neonatal Medicine,* vol. 31, no. 18, pp. 2457–2462, 2018.
8. C. Mertoğlu, M. Gunay, G. Siranli, M. Kulhan, G. Gok and Ö. Erel, “The effect of the 50 g glucose challenge test on the thiol/disulfide homeostasis in pregnancy,” *Fetal and Pediatric Pathology*, vol. 37, no. 3, pp. 147–156, 2018.
9. H. Kundi, I. Ates, E. Kiziltunc, et al., “A novel oxidative stress marker in acute myocardial infarction; thiol/disulphide homeostasis,” *American Journal of Emergency Medicine*, vol. 33, no. 11, pp. 1567–1571, 2015.
10. I. Ates, N. Ozkayar, B. Inan, et al., “Dynamic thiol/disulphide homeostasis in patients with newly diagnosed primary hypertension,” *Journal of the American Society of Hypertension*, vol. 10, no. 2, pp. 159–166, 2016.
11. E. Kızıltunç, M. Gök, H. Kundi, et al., “Plasma thiols and thiol-disulfide homeostasis in patients with isolated coronary artery ectasia,” *Atherosclerosis*, vol. 253, pp. 209–213, 2016.
12. I. H. Altıparmak, M. E. Erkuş, H. Sezen, et al., “The relation of serum thiol levels and thiol/disulphide homeostasis with the severity of coronary artery disease,” *Kardiologia Polska*, vol. 74, no. 11, pp. 1346–1353, 2016.
13. S. Sivri, H. A. Kasapkara, M. Polat, et al., “Dynamic thiol/disulphide homeostasis and its prognostic value in patients with non-ST elevation-acute coronary syndromes,” *Kardiologia Polska*, vol. 76, no. 2, pp. 426–432, 2018.
14. O. Akkuş, O. Kaypaklı, H. Koca, et al., “Thiol/disulphide homeostasis in thoracic aortic aneurysm and acute aortic syndrome,” *Biomarker in Medicine*, vol. 12, no. 4, pp. 349–358, 2018.
15. I. Ates, M. Kaplan, B. Inan, et al., “How does thiol/disulfide homeostasis change in prediabetic patients?,” *Diabetes Research and Clinical Practice*, vol. 110, no. 2, pp. 166–171, 2015.
16. B. Gulpamuk, K. Tekin, K. Sonmez, et al., “The significance of thiol/disulfide homeostasis and ischemia-modified albumin levels to assess the oxidative stress in patients with different stages of diabetes mellitus,” *Scandinavian Journal of Clinical and Laboratory Investigation,* vol. 78, no. 1-2, pp. 136–142, 2018.
17. M. A. Eren, İ. Koyuncu, H. İncebıyık, H. Karakaş, Ö. Erel and T. Sabuncu, “The evaluation of thiol/disulphide homeostasis in diabetic nephropathy,” *Diabetes Research and Clinical Practice*, vol. 18, pp. 249–253, 2019.
18. M. Polat, O. Ozcan, L. Sahan, et al., “Changes in thiol-disulfide homeostasis of the body to surgical trauma in laparoscopic cholecystectomy patients,” *Journal of Laparoendoscopic & Advanced Surgical Techniques A*, vol. 26, no. 12, pp. 992–996, 2016.
19. S. Ozyazici, F. Karateke, U. Turan, et al., “A novel oxidative stress mediator in acute appendicitis: Thiol/disulphide homeostasis,” *Mediators of Inflammation*, vol. 2016, pp. 6761050, 2016.
20. M. Ergin, M. Caliskanturk, A. Senat, O. Akturk and O. Erel, “Disulfide stress in carbon monoxide poisoning,” *Clinical Biochemistry*, vol. 49, no. 16-17, pp. 1243–1247, 2016.
21. B. B. Inal, H. O. Emre, O. Baran, et al., “Dynamic thiol-disulphide homeostasis in low-grade gliomas: Preliminary results in serum,” *Clinical Neurology and Neurosurgery*, vol. 161, pp. 17–21, 2017.
22. E. S. Parlak, M. Alisik, A. Karalezli, et al., “Are the thiol/disulfide redox status and HDL cholesterol levels associated with pulmonary embolism?: Thiol/disulfide redox status in pulmonary embolism,” *Clinical Biochemistry*, vol. 50, no. 18, pp. 1020–1024, 2017.
23. M. Gündüzöz, S. Birgin İritaş, L. Tutkun, et al., “A new potential biomarker in early diagnosis of firefighter lung function impairment: dynamic thiol/disulphide homeostasis,” *Central European Journal of Public Health*, vol. 26, no. 3, pp. 190–194, 2018.
24. M. E. Dinc, C. Ozdemir, N. N. Ayan, et al., “Thiol/disulfide homeostasis as a novel indicator of oxidative stress in obstructive sleep apnea patients,” *The Laryngoscope*, vol. 127, no. 7, pp. E244–E250, 2017.
25. O. Sengoren Dikis, M. Acat, H. Casim, et al., “The relationship of thiol/disulfide homeostasis in the etiology of patients with obstructive sleep apnea: a case-control study,” *The Aging Male*, pp. 1–8, 2019.
26. Z. K. Tufan, I. Hasanoglu, S. Kolgelier, et al., “A retrospective controlled study of thiol disulfide homeostasis as a novel marker in Crimean Congo hemorrhagic fever,” *Redox Report*, vol. 22, no. 6, pp. 241–245, 2017.
27. S. Kolgelier, M. Ergin, L. S. Demir, et al., “Impaired thiol-disulfide balance in acute brucellosis,” *Japanese Journal of Infectious Diseases*, vol. 70, no. 3, pp. 258–262, 2017.
28. Y. Ustundag-Budak, M. Sambel, M. Alisik, et al., “Thiol/disulphide homeostasis levels in erectile dysfunction patients,” *Andrologia*, vol. 49, no. 7, pp. e12695, 2017.
29. S. Coban, Y. Ustundag, A. R. Turkoglu, et al., “Thiol-disulphide balance in infertility secondary to varicocele,” *Andrologia*, pp. e13300, 2019. [Epub ahead of print]
30. T. Guney, İ. F. Kanat, A. Alkan, et al., “Assessment of serum thiol/disulfide homeostasis in multiple myeloma patients by a new method,” *Redox Report*, vol. 22, no. 6, pp. 246–251, 2017.
31. M. Hizal, M. A.N. Sendur, B. Bilgin, et al., “Evaluation of dynamic serum thiol/disulﬁde homeostasis in locally advanced and metastatic gastric cancer,” *Journal of Oncological Sciences*, vol. 4, pp. 1–4, 2018.
32. S. Neselioglu, P. B. Keske, A. A. Senat, et al., “The relationship between severity of ulcerative colitis and thiol-disulphide homeostasis,” *Bratislavske Lekarske Listy*, vol. 119, no. 8, pp. 498–502, 2018.
33. O. Özcan, H. Erdal, G. İlhan, et al., “Plasma ischemia-modified albumin levels and dynamic thiol/disulfide balance in sickle cell disease: A case-control study,” *Turkish Journal of Hematology*, vol. 35, no. 4, pp. 265–270, 2018.
34. B. Balta, M. Erdogan, M. Alisik, et al., “Does thiol-disulphide balance show oxidative stress in different MEFV mutations?,” *Rheumatology International*, vol. 38, no. 1, pp, 97–104, 2018.
35. M. Gol, B. Özkaya, C. Yildirim and R. Bal, “Regular exercise, overweight/obesity and sedentary lifestyle cause adaptive changes in thiol-disulfide homeostasis,” *Anais da Academia Brasileira de Ciências*, vol. 91, no. 2, pp. e20180547, 2019.
36. G. Vural, Ş. Gümüşyayla, O. Deniz, S. Neşelioğlu and Ö. Erel, “Relationship between thiol-disulphide homeostasis and visual evoked potentials in patients with multiple sclerosis,” *Neurological Sciences*, vol. 40, no. 2, pp. 385–391, 2019.
37. B. Balta, R. Gundogdu, M. Erdogan, et al., “Decreased disulphide/thiol ratio in patients with autosomal recessive non-syndromic hearing loss,” *International Journal of Pediatric Otorhinolaryngology*, vol. 112, pp. 188–192, 2018.
38. E. Şahin, İ. Deveci, M. E. Dinç, B. Y. Özker, C. Biçer and Ö. Erel, “Oxidative status in patients with benign paroxysmal positional vertigo,” *The Journal of International Advanced Otology*, vol. 14, no. 2, pp. 299–303, 2018.
39. E. Şimşek, C. K. Bicer, M. R. Mazlumoğlu, S. S. Kara, O. Erel and A. Çarlıoğlu, “Is otitis media with effusion associated with oxidative stress? Evaluation of thiol/disulfide homeostasis,” *American Journal of Otolaryngology*, vol. 40, no. 2, pp. 164–167, 2019.
40. G. Ayar, S. Sahin, M. U. Yazici, S. Neselioglu, O. Erel and U. S. Bayrakcı, “Effects of hemodialysis on thiol-disulphide homeostasis in critically Ill pediatric patients with acute kidney injury,” *BioMed Research International*, vol. 2018, pp.1898671, 2018.
41. M. S. Cansever, T. Zubarioglu, C. Oruc, et al., “Oxidative stress among L-2-hydroxyglutaric aciduria disease patients: evaluation of dynamic thiol/disulfide homeostasis,” *Metabolic Brain Disease*, vol. 34, no. 1, pp. 283–288, 2019.
42. S. Emre, D. D. Demirseren, M. Alisik, A. Aktas, S. Neselioglu and O. Erel, “Dynamic thiol/disulfide homeostasis and effects of smoking on homeostasis parameters in patients with psoriasis,” *Cutaneous and Ocular Toxicology*, vol. 36, no. 4, pp. 393–396, 2017.
43. A. Akbas, F. Kilinc, S. Sener, A. Aktaş, P. Baran and M. Ergin, “Investigation of thiol-disulphide balance in patients with acute urticaria and chronic spontaneous urticaria,” *Cutaneous and Ocular Toxicology*, vol. 36, no. 3, pp. 205–210, 2017.
44. S. Demir Pektas, G. Pektas, K. Tosun, G. Dogan, S. Neselioglu and O. Erel, “Evaluation of erythroid disturbance and thiol-disulphide homeostasis in patients with psoriasis,” *BioMed Research International*, vol. 2018, pp. 9548252, 2018.
45. S. Sener, A. Akbas, F. Kilinc, P. Baran, O. Erel and A. Aktas, “Thiol/disulfide homeostasis as a marker of oxidative stress in rosacea: a controlled spectrophotometric study,” *Cutaneous and Ocular Toxicology*, vol. 38, no. 1, pp. 55–58, 2019.
46. C. Topcuoglu, A. Bakirhan, F. M. Yilmaz, S. Neselioglu, O. Erel and S. Y. Sahiner, “Thiol/disulfide homeostasis in untreated schizophrenia patients,” *Psychiatry Research*, vol. 251, pp. 212–216, 2017.
47. G. Vural, S. Gumusyayla, H. Bektas, O. Deniz, M. Alisik and O. Erel, “Impairment of dynamic thiol-disulphide homeostasis in patients with idiopathic Parkinson's disease and its relationship with clinical stage of disease,” *Clinical Neurology and Neurosurgery*, vol. 153, pp. 50–55, 2017.
48. G. Erzin, V. O. Kotan, C. Topçuoğlu, et al., “Thiol/disulphide homeostasis in bipolar disorder,” *Psychiatry Research*, vol. 261, pp. 237–242, 2018.
49. M. Cingi Yirün, K. Ünal, O. Yirün, O. H. T. Kiliç and Ö. Erel, “Thiol/disulphide homeostasis in manic episode and remission phases of bipolar disorder,” *Nordic Journal of Psychiatry*, vol. 72, no. 8, pp. 572–577, 2018.
50. E. Arhan, A. N. C. Kurt, S. Neselioglu, et al., “Effects of antiepileptic drugs on dynamic thiol/disulphide homeostasis in children with idiopathic epilepsy,” *Seizure*, vol. 65, pp. 89–93, 2019.
51. B. Gulpamuk, M. Koç, M. S. Karatepe, et al., “Novel assay assessment of oxidative stress biomarkers in patients with keratoconus: Thiol-disulfide homeostasis,” *Current Eye Research*, vol. 42, no. 9, pp. 1215–1219, 2017.
52. A. Elbay, O. F. Ozer, J. C. U. Akkan, et al., “Comparison of serum thiol-disulphide homeostasis and total antioxidant-oxidant levels between exudative age-related macular degeneration patients and healthy subjects,” *International Ophthalmology*, vol. 37, no. 5, pp. 1095–1101, 2017.
53. B. Gulpamuk, U. Elgin, E. Sen, P. Yilmazbas, S. Neselioglu and O. Erel, “Evaluation of dynamic thiol-disulfide homeostasis in glaucoma patients and the correlation with retinal nerve fiber layer analysis,” *European Journal of Ophthalmology*, 1120672119839582, 2019. [Epub ahead of print]
54. Y. Karakurt, C. Mertoglu, G. Gok, et al., “Thiol-disulfide homeostasis and serum ischemia modified albumin levels in patients with primary open-angle glaucoma.” *Current Eye Research*, pp. 1-5, 2019 [Epub ahead of print].
55. F. Gul, T. Muderris, G. Yalciner, et al., “A novel method for evaluation of oxidative stress in children with OSA,” *International Journal of Pediatric Otorhinolaryngology*, vol. 89, pp. 76–80, 2016.
56. S. Avcil, P. Uysal, M. Avcil, M. Alışık and C. Biçer, “Dynamic thiol/disulfide homeostasis in children with attention deficit hyperactivity disorder and its relation with disease subtypes,” *Comprehensive Psychiatry*, vol. 73, pp. 53–60, 2017.
57. A. N. C. Kurt, H. Demir, A. Aydin and Ö. Erel, “Dynamic thiol/disulphide homeostasis in children with febrile seizure,” *Seizure*, vol. 59, pp. 34–37, 2018.
58. P. Uysal, S. Avcil, S. Neşelioğlu, C. Biçer and F. Çatal, “Association of oxidative stress and dynamic thiol-disulphide homeostasis with atopic dermatitis severity and chronicity in children: a prospective study,” *Clinical and Experimental Dermatology*, vol. 43, no. 2, pp. 124–130, 2018.
59. S. Yaşar Durmuş, N. M. Şahin, M. Ergin, S. Neşelioğlu, Z. Aycan and Ö. Erel, “How does thiol/disulfide homeostasis change in children with type 1 diabetes mellitus?,” *Diabetes Research and Clinical Practice*, vol. 149, pp. 64–68, 2019.
60. G. Ayar, S. Sahin, Y. Men Atmaca, M. Uysal Yazici, S. Neselioglu and O. Erel, “Thiol-disulphide homeostasis is an oxidative stress indicator in critically ill children with sepsis,” *Archivos Argentinos de Pediatria*, vol. 117, no. 3, pp. 143–148, 2019.
61. H. Halil, N. Tuygun, E. Aksoy, O. Erel and C. D. Karacan, “Serum thiol - disulphide levels in epileptic pediatric patients,” *Combinatorial Chemistry & High Throughput Screen*, vol. 22, no. 1, pp. 65–68, 2019.
62. A. Guzelcicek, G. Cakirca, O. Erel and A. Solmaz, “Assessment of thiol/disulfide balance as an oxidative stress marker in children with β-thalassemia major,” *Pakistan Journal of Medical Science*, vol. 35, no. 1, pp. 161–165, 2019.
63. B. Elmas, T. Yildiz, H. Yazar, et al., “New oxidative stress markers useful in the diagnosis of acute appendicitis in children: thiol/disulfide homeostasis and the asymmetric dimethylarginine level,” *Pediatric Emergency Care*, Nov 14, 2017. [Epub ahead of print]
64. E. Altinel Acoglu, O. Erel, F. Yazilitas, et al., “Changes in thiol/disulfide homeostasis in juvenile idiopathic arthritis,” *Pediatrics International*, vol. 60, no. 6, pp. 593–596, 2018.

**Oxidative stress index (OSI)**

- - - 1. M. Kosecik, O. Erel, E. Sevinc and S. Selek, “Increased oxidative stress in children exposed to passive smoking,” *International Journal of Cardiology*, vol. 100, no. 1, pp. 61–64, 2005.
      2. A. Ece, Y. Atamer, F. Gürkan, M. Davutoğlu, Y. Koçyiğit and M. Tutanç, “Paraoxonase, total antioxidant response, and peroxide levels in children with steroid-sensitive nephrotic syndrome,” *Pediatric Nephrology*, vol. 20, no. 9, pp. 1279–1284, 2005.
      3. A. Aycicek, O. Erel and A. Kocyigit, “Increased oxidative stress in infants exposed to passive smoking,” *European Journal of Pediatrics*, vol. 164, no. 12, pp. 775–778, 2005.
      4. A. Aycicek, O. Erel and A. Kocyigit, “Decreased total antioxidant capacity and increased oxidative stress in passive smoker infants and their mothers,” *Pediatrics International*, vol, 47, no. 6, pp. 635–639, 2005.
      5. A. Ece, Y. Atamer, F. Gürkan, et al., “Paraoxonase, anti-oxidant response and oxidative stress in children with chronic renal failure,” *Pediatric Nephrology*, vol. 21, no. 2, pp. 239–245, 2006.
      6. A. Aycicek and A. Iscan, “The effects of carbamazepine, valproic acid and phenobarbital on the oxidative and antioxidative balance in epileptic children,” *European Neurology*, vol. 57, no. 2, pp. 65–69, 2007.
      7. Z. Baysal, T. Togrul, N. Aksoy, et al., “Evaluation of total oxidative and antioxidative status in pediatric patients undergoing laparoscopic surgery,” *Journal of Pediatric Surgery*, vol. 44, no. 7, pp. 1367–1370, 2009.
      8. A. Cakmak, M. Soker, A. Koc and O. Erel, “Paraoxonase and arylesterase activity with oxidative status in children with thalassemia major,” *Journal of Pediatric Hematology/Oncology*, vol. 31, no. 8, pp. 583–587, 2009.
      9. S. Ercan, A. Cakmak, M. Kösecik and O. Erel, “The oxidative state of children with cyanotic and acyanotic congenital heart disease,” *Anadolu Kardiyoloji Dergisi*, vol. 9, no. 6, pp. 486–490, 2009.
      10. S. Akbayram, M. Doğan, C. Akgün, et al., “The association of oxidant status and antioxidant capacity in children with acute and chronic ITP,” *Journal of Pediatric Hematology/Oncology*, vol. 32, no. 4, pp. 277–281, 2010.
      11. F. Yıldırım, K. Sermetow, A. Aycicek, A. Kocyigit and O. Erel, “Increased oxidative stress in preschool children exposed to passive smoking.” *Jornal de Pediatria*, vol. 87, no. 6, pp. 523–528, 2011.
      12. E. A. Hamed, T. B. El-Abaseri, A. O. Mohamed, A. R. Ahmed and T. H. El-Metwally, “Hypoxia and oxidative stress markers in pediatric patients undergoing hemodialysis: cross section study,” *BMC Nephrology*, vol. 13, pp. 136, 2012.
      13. M. Celik, K. Sermatov, M. Abuhandan, D. Zeyrek, A. Kocyigit and A. Iscan, “Oxidative status and DNA damage in chidren with marasmic malnutrition,” *Journal of Clinical Laboratory Analysis*, vol. 26, no. 3, pp. 161–166, 2012.
      14. R. Dundaroz, U. Erenberk, O. Turel, A. D. Demir, E. Ozkaya and O. Erel, “Oxidative and antioxidative status of children with acute bronchiolitis” *Jornal de Pediatria*, vol. 89, no. 4, pp. 407–411, 2013.
      15. M. Calik, M. Abuhandan, A. Aycicek, A. Taskin, S. Selek and A. Iscan, “Increased oxidant status in children with breath-holding spells,” *Child´s Nervous System*, vol. 29, no. 6, pp. 1015–1019, 2013.
      16. M. Abuhandan, F. Bozkuş, N. Demir, et al., “The preoperative and postoperative oxidative status of children with chronic adenotonsillar hypertrophy,” *La Clinica Terapeutica*, vol. 164, no. 3, pp. e163–167, 2013.
      17. H. Kandemir, M. Abuhandan, N. Aksoy, E. Savik and C. Kaya, “Oxidative imbalance in child and adolescent patients with obsessive compulsive disorder,” *Journal of Psychiatric Research*, vol. 47, no. 11, pp. 1831–1834, 2013.
      18. E. Eren, M. Abuhandan, A. Solmaz and A. Taşkın, “Serum paraoxonase/arylesterase activity and oxidative stress status in children with metabolic syndrome,” *Journal of Clinical Research in Pediatric Endocrinology*, vol. 6, no. 3, pp. 163–168, 2014.
      19. E. Torun, A. H. Gedik, E. Cakir, T. Umutoglu, O. Gok and U. Kilic, “Serum paraoxonase 1 activity and oxidative stress in pediatric patients with pulmonary tuberculosis,” *Medical Principles and Practice*, vol. 23, no. 5, pp. 426–431, 2014.
      20. S. Petrovic, N. Bogavac-Stanojevic, J. Kotur-Stevuljevic, et al., “Oxidative status parameters in children with urinary tract infection.” *Biochemia Medica*, vol. 24, no. 2, pp. 266–272, 2014.
      21. E. Guney, M. Fatih Ceylan, A. Tektas, et al., “Oxidative stress in children and adolescents with anxiety disorders,” *Journal of Affective Disorders*, vol. 156, pp. 62–66, 2014.
      22. E. Torun, S. Gökçe, İ. T. Ozgen, S. Aydın and Y. Cesur, “Serum paraoxonase activity and oxidative stress and their relationship with obesity-related metabolic syndrome and non-alcoholic fatty liver disease in obese children and adolescents,” *Journal of Pediatric Endocrinology & Metabolism*, vol. 27, no. 7-8, pp. 667–675, 2014.
      23. Z. C. Ozdemir, A. Koc, A. Aycicek and A. Kocyigit, “N-Acetylcysteine supplementation reduces oxidative stress and DNA damage in children with β-thalassemia,” *Hemoglobin*, vol. 38, no. 5, pp. 359–364, 2014.
      24. C. Kaya, A. Ataş, N. Aksoy, E. C. Kaya and M. Abuhandan, “Evaluation of pre-treatment and post-treatment S100B, oxidant and antioxidant capacity in children with diabetic cetoacidosis,” *Journal of Clinical Research in Pediatric Endocrinology*, vol. 7, no. 2, pp. 109–113, 2015.
      25. C. S. Sim, J. H. Lee, S. H. Kim, et al., “Oxidative stress in schoolchildren with allergic rhinitis: propensity score matching case-control study,” *Annals of Allergy, Asthma & Immunology*, vol. 115, no. 5, pp. 391–395, 2015.
      26. H. Sezen, H. Kandemir, E. Savik, et al., “Increased oxidative stress in children with attention deficit hyperactivity disorder,” *Redox Report*, vol. 21, no. 6, pp. 248–253, 2016.
      27. H. Kurku, A. Gencer, O. Pirgon, M. Buyukinan and N. Aslan, “Increased oxidative stress parameters in children with moderate iodine deficiency,” *Journal of Pediatric Endocrinology & Metabolism*, vol. 29, no. 10, pp. 1159–1164, 2016.
      28. G. Rowicka, H. Dyląg, J. Ambroszkiewicz, A. Riahi, H. Weker and M. Chełchowska, “Total oxidant and antioxidant status in prepubertal children with obesity,” *Oxidative Medicine and Cellular Longevity*, vol. 2017, pp. 5621989, 2017.
      29. F. U. Kahraman, E. Torun, N. K. Osmanoğlu, S. Oruçlu and Ö. F. Özer, “Serum oxidative stress parameters and paraoxonase-1 in children and adolescents exposed to passive smoking,” *Pediatrics International*, vol. 59, no. 1, pp. 68–73, 2017.
      30. F. H. Altin, H. A. Yildirim, I. C. Tanidir, et al., “Alterations in antioxidant and oxidant status of children after on-pump surgery for cyanotic and acyanotic congenital heart diseases,” *Cardiology in the Young*, vol. 27, no. 2, pp. 325–332, 2017.
      31. R. López-Vargas, A. Méndez-Serrano, A. Albores-Medina, et al., “Oxidative stress index is increased in children exposed to industrial discharges and is inversely correlated with metabolite excretion of voc,” *Environmental and Molecular Mutagenesis*, vol. 59, no. 7, pp. 639–652, 2018.
      32. G. Rowicka, G. Czaja-Bulsa, M. Chełchowska, et al., “Oxidative and antioxidative status of children with celiac disease treated with a gluten free-diet,” *Oxidative Medicine and Cellular Longevity*, vol. 2018, pp. 1324820, 2018.
      33. M. Igde, P. Baran, B. G. Oksuz, S. Topcuoglu and G. Karatekin, “Association between the oxidative status, Vitamin D levels and respiratory function in asthmatic children,” *Nigerian Journal of Clinical Practice*, vol.21, no. 1, pp. 63–68, 2018.
      34. A. Aycicek and O. Erel, “Total oxidant/antioxidant status in jaundiced newborns before and after phototherapy,” *Jornal de Pediatria*, vol. 83, no.4, pp. 319–322, 2007.
      35. G. Demirel, N. Uras, I. H. Celik, et al., “Comparison of total oxidant/antioxidant status in unconjugated hyperbilirubinemia of newborn before and after conventional and LED phototherapy: A prospective randomized controlled trial,” *Clinical and Investigative Medicine*, vol. 55, no. 5, pp. E335–341, 2010.
      36. C. Aydemir, D. Dilli, N. Uras, et al., “Total oxidant status and oxidative stress are increased in infants with necrotizing enterocolitis,” *Journal of Pediatric Surgery*, vol. 46, no. 11, pp. 2096–2100, 2011.
      37. M. Doğan, E. Peker, E. Kirimi, et al., “Evaluation of oxidant and antioxidant status in infants with hyperbilirubinemia and kernicterus,” *Human and Experimental Toxicology*, vol. 30, no. 11, pp. 1751–1760, 2011.
      38. Y. U. Sarikabadayi, O. Aydemir, C. Aydemir, et al., “Umbilical cord oxidative stress in infants of diabetic mothers and its relation to maternal hyperglycemia,” *Journal of Pediatric Endocrinology & Metabolism*, vol. 24, no. 9-10, pp. 671–674, 2011.
      39. E. S. Guvendag Guven, D. Karcaaltincaba, O. Kandemir, S. Kiykac and A. Mentese, “Cord blood oxidative stress markers correlate with umbilical artery pulsatility in fetal growth restriction,” *Journal Maternal-Fetal & Neonatal Medicine*, 2013; 26(6): 576-80.
      40. G. Sandal, B. Mutlu, N. Uras, O. Erdeve, S. S. Oguz and U. Dilmen, “Evaluation of treatment with hydrocortisone on oxidant/antioxidant system in preterm infants with BPD,” *European Review for Medical and Pharmacological Sciences*, vol. 17, no. 19, pp. 259–2597, 2013.
      41. N. Demir, İ. Ece, E. Peker, et al., “Impact of patent ductus arteriosus and subsequent therapy with ibuprofen on the release of S-100B and oxidative stress index in preterm infants.” *Medical Science Monitor*, vol. 20, pp. 2799–2805, 2014.
      42. M. Aksin, A. Incebiyik, M. Vural, et al., “Does a risky outcome of antenatal screening test indicate oxidative stress?,” *Journal Maternal-Fetal & Neonatal Medicine*, vol. 27, no. 10, pp. 1033–1037, 2014.
      43. A. Annagür, R. Örs, H. Altunhan, et al., “Total antioxidant and total oxidant states, and serum paraoxonase-1 in neonatal sepsis,” *Pediatrics International*, vol. 57, no. 4, pp. 608–613, 2015.
      44. S. Topcuoglu, G. Karatekin, T. Yavuz, et al., “The relationship between the oxidative stress and the cardiac hypertrophy in infants of diabetic mothers,” *Diabetes Research and Clinical Practice,* vol. 109, no. 1, pp. 104–109, 2015.
      45. S. Karabayırlı, E. A. Keskin, A. Kaya, et al., “Assessment of fetal antioxidant and oxidant status during different anesthesia techniques for elective cesarean sections,” *Journal of Research in Medical Sciences*, vol. 20, no. 8, pp. 739–744, 2015.
      46. A. Dursun, N. Okumuş, S. Erol, T. Bayrak and A. Zenciroğlu, “Effect of ventilation support on oxidative stress and ischemia-modified albumin in neonates,” *American Journal of Perinatology*, vol. 33, no. 2, pp. 136–142, 2016.
      47. A. Allam, S. R. Ravikiran, B. S. Baliga, K. Bhat and N. Joseph, “Effect of conventional and LED phototherapy on the antioxidant-oxidant status in preterm neonates with jaundice,” *Indian Pediatrics*, vol. 54, no. 8, pp. 644–646, 2017.
      48. E. Özalkaya, G. Karatekin, S. Topçuoğlu, et al., “Neonatology oxidative status in preterm infants with premature preterm rupture of membranes and fetal inflammatuar response syndrome,” *Pediatrics & Neonatology*, vol. 58, no. 5, pp. 437–441, 2017.
      49. M. Konak, N. Tarakci, H. Altunhan, A. Annagür, A. Toker and R. Örs, “Total antioxidant, total oxidant and serum paraoxonase levels according to lipid administration method in parenterally fed premature infants,” *Journal Maternal-Fetal & Neonatal Medicine*, vol. 30, no. 14, pp. 1734–1738, 2017.
      50. S. Unal, N. Demirel, S. Erol, et al., “Effects of two different lipid emulsions on morbidities and oxidant stress statuses in preterm infants: an observational,” *Journal Maternal-Fetal & Neonatal Medicine*, vol. 31, no. 7, pp. 850–856, 2018.
      51. N. Yilmaz, O. Erel, M. Hazer, C. Bağci, E. Namiduru and E. Gül, “Biochemical assessments of retinol, alpha-tocopherol, pyridoxal--5-phosphate oxidative stress index and total antioxidant status in adolescent professional basketball players and sedentary controls,” *International Journal of Adolescent Medicine and Health*, vol. 19, no. 2, pp. 177–186, 2007.
      52. Ö. Pirgon, H. Bilgin, F. Çekmez, H. Kurku and B. N. Dündar, “Association between insulin resistance and oxidative stress parameters in obese adolescents with non-alcoholic fatty liver disease,” *Journal of Clinical Research in Pediatric Endocrinology*, vol. 5, no. 1, pp. 33–39, 2013.
      53. M. Rabus, R. Demirbag, A. Yildiz, et al., “Association of prolidase activity, oxidative parameters, and presence of atrial fibrillation in patients with mitral stenosis,” *Archives of Medical Research*, vol. 39, no. 5, pp. 519–524, 2008.
      54. M. Rabus, R. Demirbağ, Y. Sezen, et al., “Plasma and tissue oxidative stress index in patients with rheumatic and degenerative heart valve disease,” *Turk Kardiyoloji Dernegi Arsivi*, vol. 36, no. 8, pp. 536–540, 2008.
      55. M. C. Chen, J. P. Chang, W. H. Liu, et al., “Increased serum oxidative stress in patients with severe mitral regurgitation: a new finding and potential mechanism for atrial enlargement,” *Clinical Biochemistry*, vol. 42, no. 10-11, pp. 943–948, 2009.
      56. Y. Sezen, M. Bas, M. Polat, et al., “The relationship between oxidative stress and coronary artery ectasia,” *Cardiology Journal*, vol. 17, no. 5, pp. 488–494, 2010.
      57. H. Buyukhatipoglu, Y. Sezen, A. Yildiz, et al., “N-acetylcysteine fails to prevent renal dysfunction and oxidative stress after noniodine contrast media administration during percutaneous coronary interventions,” *Polskie Archiwum Medycyny Wewnętrznej*, vol. 120, no. 10, pp. 383–389, 2010.
      58. S. Başyığıt, H. Akbaş, İ. Süleymanlar, D. Kemaloğlu, S. Koç and G. Süleymanlar, “The assessment of carotid intima-media thickness, lipid profiles and oxidative stress markers in *Helicobacter pylori*-positive subjects,” *The Turkish Journal of Gastroenterology*, vol. 23, no. 6, pp. 646–651, 2012.
      59. S. Aksoy, N. Cam, U. Gurkan, et al., “Oxidative stress and severity of coronary artery disease in young smokers with acute myocardial infarction,” *Cardiology Journal*, vol. 19, no. 4, pp. 381–386, 2012.
      60. H. Yucel, M. Ozaydin, A. Dogan, D. Erdogan, A. Icli and R. Sutcu, “Evaluation of plasma oxidative status in patients with slow coronary flow,” *Kardiologia Polska*, vol. 71, no. 6, pp. 588–594, 2013.
      61. M. T. Gökdemir, H. Kaya, O. Söğüt, Z. Kaya, L. Albayrak and A. Taşkın, “The role of oxidative stress and inflammation in the early evaluation of acute non-ST-elevation myocardial infarction: an observational study,” *Anadolu Kardiyoloji Dergisi*, vol. 13, no. 2, pp. 131–136, 2013.
      62. Z. Karataş, T. Baysal, F. Sap, H. Altın and H. Çiçekler, “The role of tenascin-C and oxidative stress in rheumatic and congenital heart valve diseases: an observational study,” *Anadolu Kardiyoloji Dergisi*, vol. 13, no. 4, pp. 350–356, 2013.
      63. O. Karahan, S. Manduz, G. Bektasoglu, A. Zorlu, K. A. Turkdogan and S. Bozok, “A high oxidative stress index predicts endothelial dysfunction in young male smokers,” *Bratislavske Lekarske Listy*, vol. 114, no. 12, pp. 721–725, 2013.
      64. N. Yilmaz, N. Simsek, O. Aydin, et al., “Decreased paraoxonase 1, arylesterase enzyme activity, and enhanced oxidative stress in patients with mitral and aortic valve insufficiency,” *Clinical Laboratory*, vol. 59, no. 5-6, pp. 597–604, 2013.
      65. U. Mentese, O. V. Dogan, I. Turan, et al., “Oxidant-antioxidant balance during on-pump coronary artery bypass grafting,” *Scientific World Journal*, vol. 2014, pp. 263058, 2014.
      66. M. Karabacak, A. Dogan, S. Tayyar and H. A. Bas, “Oxidative stress status increase in patients with nonischemic heart failure,” *Medical Principles and Practice*, vol. 23, no. 6, pp. 532–537, 2014.
      67. M. Gür, C. Türkoğlu, A. Taşkın, et al., “Paraoxonase-1 activity and oxidative stress in patients with anterior ST elevation myocardial infarction undergoing primary percutaneous coronary intervention with and without no-reflow,” *Atherosclerosis*, vol. 234, no. 2, pp. 415–420, 2014.
      68. E. Eren, H. Y. Ellidag, Y. Cekin, R. U. Ayoglu, A. O. Sekercioglu and N. Yılmaz, “Heart valve disease: the role of calcidiol deficiency, elevated parathyroid hormone levels and oxidative stress in mitral and aortic valve insufficiency,” *Redox Report*, vol. 19, no. 1, pp. 34–39, 2014.
      69. H. Y. Ellidag, E. Eren, N. Yılmaz and Y. Cekin, “Oxidative stress and ischemia-modified albumin in chronic ischemic heart failure,” *Redox Report*, vol. 19, no. 3, pp. 118–123, 2014.
      70. F. Akkafa, I. Halil Altiparmak, M. E. Erkus, et al., “Reduced SIRT1 expression correlates with enhanced oxidative stress in compensated and decompensated heart failure,” *Redox Biology*, vol. 6, pp. 169–173, 2015.
      71. H. Yücel, K. A. Türkdoğan, A. Zorlu, H. Aydın, R. Kurt and M. B. Yılmaz, “Association between oxidative stress index and post-CPR early mortality in cardiac arrest patients: A prospective observational study,” *Anatolian Journal of Cardiology*, vol. 15, no. 9, pp. 737–743, 2015.
      72. T. Turan, Ü. Menteşe, M. T. Ağaç, et al., “The relation between intensity and complexity of coronary artery lesion and oxidative stress in patients with acute coronary syndrome,” *Anatolian Journal of Cardiology*, vol. 15, no. 10, pp. 795–800, 2015.
      73. M. Karabacak, A. Dogan, S. Tayyar and H. A. Bas, “The effects of carvedilol and nebivolol on oxidative stress status in patients with non-ischaemic heart failure,” *Kardiologia Polska*, vol. 73, no. 3, pp. 201–206, 2015.
      74. A. Börekçi, M. Gür, C. Türkoğlu, et al., “Oxidative stress and paraoxonase 1 activity predict contrast-induced nephropathy in patients with ST-segment elevation myocardial infarction undergoing primary percutaneous coronary intervention,” *Angiology*, vol. 66, no. 4, pp. 339–345, 2015.
      75. F. Içme, Ö. Erel, A. Avci, S. Satar, M. Güle and S. Acehan, “The relation between oxidative stress parameters, ischemic stroke, and hemorrhagic stroke,” *Turkish Journal of Medical Sciences*, vol. 45, no. 4, pp. 947–953, 2015.
      76. C. Türkoğlu, M. Gür, T. Şeker, Ş. Selek and A. Koçyiğit, “The predictive value of M30 and oxidative stress for left ventricular remodeling in patients with anterior ST-segment elevation myocardial infarction treated with primary percutaneous coronary intervention,” *Coronary Artery Disease*, vol. 27, no. 8, pp. 690–695, 2016.
      77. W. Kuliczkowski, R. Golanski, M. Bijak, et al., “Relationship between high on aspirin platelet reactivity and oxidative stress in coronary artery by-pass grafted patients,” *Blood Coagulation and Fibrinolysis*, vol. 27, no. 2, pp. 151–155, 2016.
      78. A. Börekçi, M. Gür, C. Türkoğlu, et al., “Oxidative stress and spontaneous reperfusion of infarct-related artery in patients with ST-segment elevation myocardial infarction,” *Clinical and Applied Thrombosis/Hemostasis*, vol. 22, np. 2, pp. 171–177, 2016.
      79. U. Menteşe, I. Turan, S. Usta, et al., “Systemic oxidant/antioxidant balance in human abdominal aortic aneurysm,” *Perfusion*, vol. 31, no. 4, pp. 288–294, 2016.
      80. B. Inan, I. Ates, N. Ozkayar, et al., “Are increased. oxidative stress and asymmetric dimethylarginine levels associated with masked hypertension?,” *Clinical and Experimental Hypertension*, vol. 38, no. 3, pp. 294–298, 2016.
      81. O. Kaypaklı, M. Gür, H. Harbalıoğlu, T. Şeker and Ş. Selek, “High morning blood pressure surge is associated with oxidative stress and paraoxonase 1 activity in newly diagnosed hypertensive patients,” *Clinical and Experimental Hypertension*, vol. 38, no. 8, pp. 680–685, 2016.
      82. C. Kilit, F. E. Koçak and T. Paşalı Kilit, “Comparison of the effects of high-dose atorvastatin and high-dose rosuvastatin on oxidative stress in patients with acute myocardial infarction: A pilot study,” *Turk Kardiyoloji Dernegi Arsivi*, vol. 45, no. 3, pp. 235–243, 2017.
      83. S. Shahzad, S. Mateen, A. Hasan and S. Moin, “GRACE score of myocardial infarction patients correlates with oxidative stress index, hsCRP and inflammation,” *Immunobiology*, pii: S0171-2985(18)30212-2, 2019. [Ahead of print]
      84. A. Aycicek and A. Ipek, “Maternal active or passive smoking causes oxidative stress in cord blood,” *European Journal of Pediatrics*, vol. 167, no. 1, pp. 81–85, 2008.
      85. H. Toy, H. Camuzcuoglu, D. T. Arioz, S. Kurt, H. Celik and N. Aksoy, “Serum prolidase activity and oxidative stress markers in pregnancies with intrauterine growth restricted infants,” *The Journal of Obstetric and Gynaecology Research*, vol. 35, no. 6, pp. 1047–1053, 2009.
      86. E. Ozturk, O. Balat, M. G. Ugur, et al., “Effect of Ramadan fasting on maternal oxidative stress during the second trimester: a preliminary study,” *The Journal of Obstetric and Gynaecology Research*, vol. 37, no. 7, pp. 729–733, 2011.
      87. R. Aliyazicioglu, S. Guven, A. Mentese, et al., “Serum anti-carbonic anhydrase II antibodies and oxidant-antioxidant balance in pre-eclampsia,” *American Journal of Reproductive Immunology*, vol. 66, no. 4, pp. 297–303, 2011.
      88. A. Aycicek, M. Varma, K. Ahmet, K. Abdurrahim and O. Erel, “Maternal active or passive smoking causes oxidative stress in placental tissue,” *European Journal of Pediatrics*, vol. 170, no. 5, pp. 645–651, 2011.
      89. B. Mutlu, N. Aksoy, H. Cakir, H. Celik and O. Erel, “The effects of the mode of delivery on oxidative-antioxidative balance,” *Journal Maternal-Fetal & Neonatal Medicine*, vol. 24, no. 11, pp. 1367–1370, 2011.
      90. M. Erdem, M. Harma, I. M. Harma, I. Arikan and A. Barut, “Comparative study of oxidative stress in maternal blood with that of cord blood and maternal milk,” *Archives of Gynecology and Obstetrics*, vol. 285, no. 2, pp. 371–375, 2012.
      91. B. Mutlu, A.Y. Bas, N. Aksoy and A. Taskin, “The effect of maternal number of births on oxidative and antioxidative systems in cord blood,” *Journal Maternal-Fetal & Neonatal Medicine*, vol. 25, no. 6, pp. 802–805, 2012.
      92. N. Hilali, A. Kocyigit, M. Demir, et al., “DNA damage and oxidative stress in patients with mild preeclampsia and offspring,” *European Journal of Obstetrics & Gynecology and Reproductive Biology*, vol. 170, no. 2, pp. 377–380, 2013.
      93. N. Hilali, N. Aksoy, M. Vural, H. Camuzcuoglu and A. Taskin, “Oxidative status and serum prolidase activity in tubal ectopic pregnancy,” *The Journal of the Pakistan Medical Association*, vol. 63, no. 2, pp. 169–172, 2013.
      94. S. Yalcin, T. Ulas, M. A. Eren, et al., “Relationship between oxidative stress parameters and cystatin C levels in patients with severe preeclampsia,” *Medicina*, vol. 49, no. 3, pp. 118–123, 2013.
      95. S. Yalcin, H. Aydoğan, H. H. Yuce, et al., “Effects of sevoflurane and desflurane on oxidative stress during general anesthesia for elective cesarean section,” *Wiener Klinische Wochenschrift*, vol. 125, no. 15-16, pp. 467–473, 2013.
      96. M. E. Demir, T. Ulas, M. S. Dal, et al., “Oxidative stress parameters and ceruloplasmin levels in patients with severe preeclampsia,” *La Clinica Terapeutica*, vol. 164, no. 2, pp. e83–87, 2013.
      97. Ö. B. Yiyenoğlu, M. G. Uğur, H. Ç. Özcan, et al., “Assessment of oxidative stress markers in recurrent pregnancy loss: a prospective study,” *Archives of Gynecology and Obstetrics*, vol. 289, no. 6, pp. 1337–1340, 2014.
      98. A. Incebiyik, M. Vural, A. Camuzcuoglu, et al., “Comparison of tissue prolidase enzyme activity and serum oxidative stress level between pregnant women with placental abruption and those with a healthy pregnancy,” *Archives of Gynecology and Obstetrics*, vol. 291, no. 4, pp. 805–809, 2015.
      99. S. Yilmaz, A. S. Ozgu-Erdinc, C. Demirtas, G. Ozturk, S. Erkaya and D. Uygur, “The oxidative stress index increases among patients with hyperemesis gravidarum but not in normal pregnancies,” *Redox Report*, vol. 20, no. 3, pp. 97–102, 2015.
      100. A. Turgut, A. Ozler, N. Y. Goruk, et al., “Serum levels of the adipokines, free fatty acids, and oxidative stress markers in obese and non-obese preeclamptic patients,” *Clinical and Experimental Obstetrics & Gynecology*, vol. 42, no. 4, pp. 473–479, 2015.
      101. M. A. Camkurt, E. Fındıklı, F. İ. Tolun, et al., “Probable preventive effects of placenta from oxidative stress; Evaluation of total antioxidant status, total oxidant status and oxidative stress index in fetal cord blood during the delivery.” *Psychiatry Research*, vol. 240, pp. 222–225, 2016.
      102. E. Oztas, S. Ozler, A. Tokmak, et al., “Oxidative stress markers in severe preeclampsia and preeclampsia-related perinatal morbidity - preliminary report.” *Ginekologia Polska*, vol. 87, no. 6, pp. 436–441, 2016.
      103. M. Namlı Kalem, N. Akgun, Z. Kalem, B. Bakirarar and T. Celik, “Chemokine (C-C motif) ligand-2 (CCL2) and oxidative stress markers in recurrent pregnancy loss and repeated implantation failure,” *Journal of the Assisted Reproduction and Genetics*, vol. 34, no. 11, pp. 1501–1506, 2017.
      104. M. Grzesiak, Z. Gaj, R. Kocyłowski, et al., “Oxidative stress in women treated with atosiban for impending preterm birth,” *Oxidative Medicine and Cellular Longevity*, vol. 2018, pp. 3919106, 2018.
      105. S. Selek, H. Herken, M. Bulut, et al., “Oxidative imbalance in obsessive compulsive disorder patients: a total evaluation of oxidant-antioxidant status,” *Progress in Neuro-Psychopharmacology & Biological Psychiatry*, vol. 32, no. 2, pp. 487–491, 2008.
      106. M. Yumru, H. A. Savas, A. Kalenderoglu, M. Bulut, H. Celik and O. Erel, “Oxidative imbalance in bipolar disorder subtypes: a comparative study,” *Progress in Neuro-Psychopharmacology & Biological Psychiatry*, vol. 33, no. 6, pp. 1070–1074, 2009.
      107. B. E. Cumurcu, H. Ozyurt, I. Etikan, S. Demir and R. Karlidag, “Total antioxidant capacity and total oxidant status in patients with major depression: impact of antidepressant treatment,” *Psychiatry and Clinical Neurosciences*, vol. 63, no. 5, pp. 639–645, 2009.
      108. M. O. Akiibinu, O. A. Ogundahunsi and E. O. Ogunyemi, “Inter-relationship of plasma markers of oxidative stress and thyroid hormones in schizophrenics,” *BMC Research Notes*, vol. 5, pp. 169, 2012.
      109. S. Selek, M. Bulut, A. R. Ocak, A. Kalenderoğlu and H. A. Savaş, “Evaluation of total oxidative status in adult attention deficit hyperactivity disorder and its diagnostic implications,” *Journal of Psychiatric Research*, vol. 46, no. 4, pp. 451–455, 2012.
      110. T. Ulas, H. Buyukhatipoglu, I. Kirhan, et al., “The effect of day and night shifts on oxidative stress and anxiety symptoms of the nurses,” *European Review for Medical and Pharmacological Sciences*, vol. 16, no. 5, pp. 594–599, 2012.
      111. Y. Albayrak, C. Ünsal, M. Beyazyüz, A. Ünal and M. Kuloğlu, “Reduced total antioxidant level and increased oxidative stress in patients with deficit schizophrenia: a preliminary study,” *Progress in Neuro-Psychopharmacology & Biological Psychiatry*, vol. 45, pp. 144–149, 2013.
      112. M. H. Kokacya, B. Bahceci, I. Bahceci, A. R. Dilek and R. Dokuyucu, “Prolidase activity and oxidative stress in patients with major depressive disorder,” *Psychiatria Danubina*, vol. 26, no. 4, pp. 314–318, 2014.
      113. T. Kalelioglu, A. Genc, N. Karamustafalioglu, et al., “Initial and post-treatment total oxidant-antioxidant status and oxidative stress index in male patients with manic episode,” *Psychiatry Research*, vol. 218, no. 1-2, pp. 249–251, 2014.
      114. A. Kirbas, S. Kirbas, M. C. Cure and A. Tufekci, “Paraoxonase and arylesterase activity and total oxidative/anti-oxidative status in patients with idiopathic Parkinson's disease,” *Journal of Clinical Neuroscience*, vol. 21, no. 3, pp. 451–455, 2014.
      115. F. Bulbul, O. Virit, G. Alpak, et al., “Are oxidative stress markers useful to distinguish schizoaffective disorder from schizophrenia and bipolar disorder?,” *Acta Neuropsychiatrica*, vol. 26, no. 2, pp. 120–124, 2014.
      116. U. Sertan Copoglu, O. Virit, M. Hanifi Kokacya, et al., “Increased oxidative stress and oxidative DNA damage in non-remission schizophrenia patients,” *Psychiatry Research*, vol. 229, no. 1-2, pp. 200–205, 2015.
      117. E. Guney, F. H. Cetin, M. Alisik, et al., “Attention deficit hyperactivity disorder and oxidative stress: A short term follow up study,” *Psychiatry Research*, vol. 229, no. 1-2, pp. 310–317, 2015.
      118. A. Emhan, S. Selek, H. Bayazıt, İ. Fatih Karababa, M. Katı and N. Aksoy, “Evaluation of oxidative and antioxidative parameters in generalized anxiety disorder,” *Psychiatry Research*, vol. 230, no. 3, pp. 806–810, 2015.
      119. B. Bahceci, M. H. Kokacya, U. S. Copoglu, et al., “Elevated nucleosome level and oxidative stress in schizophrenia patients,” *Bratislavske Lekarske Listy*, vol. 116, no. 10, pp. 587–590, 2015.
      120. P. G. Ozdemir, İ. Kaplan, C. Uysal, et al., “Serum total oxidant and antioxidant status in earthquake survivors with post-traumatic stress disorder,” *Acta Neuropsychiatrica*, vol. 27, no. 3, pp. 153–158, 2015.
      121. K. Kriisa, L. Haring, E. Vasar, et al., “Antipsychotic treatment reduces indices of oxidative stress in first-episode psychosis patients,” *Oxidative Medicine and Cellular Longevity*, vol. 2016, pp. 9616593, 2016.
      122. D. Alici, F. Bulbul, O. Virit, et al., “Evaluation of oxidative metabolism and oxidative DNA damage in patients with obsessive-compulsive disorder,” *Psychiatry and Clinical Neurosciences*, vol. 70, no. 2, pp. 109–115, 2016.
      123. A. C. Ercan, B. Bahceci, S. Polat, et al., “Oxidative status and prolidase activities in generalized anxiety disorder,” *Asian Journal of Psychiatry*, vol. 25, pp. 118–122, 2017.
      124. İ. F. Karababa, S. N. Savas, S. Selek, et al., “Homocysteine levels and oxidative stress parameters in patients with adult ADHD,” *Journal of Attention Disorders*, vol. 21, no. 6, pp. 487–493, 2017.
      125. O. Ethemoglu, H. Ay, I. Koyuncu and A. Gönel, “Comparison of cytokines and prooxidants/antioxidants markers among adults with refractory versus well-controlled epilepsy: A cross-sectional study,” *Seizure*, vol. 60, pp. 105–109, 2018.
      126. C. Bolukbas, F. F. Bolukbas, M. Horoz, M. Aslan, H. Celik and O. Erel “Increased oxidative stress associated with the severity of the liver disease in various forms of hepatitis B virus infection,” *BMC Infectious Diseases*, vol. 5, pp. 95, 2005.
      127. M. Horoz, C. Bolukbas, F. F. Bolukbas, et., “Oxidative stress in hepatitis C infected end-stage renal disease subjects,” *BMC Infectious Diseases*, vol. 6, pp. 114, 2006.
      128. M. Aslan, M. Horoz, Y. Nazligul, et al., “Insulin resistance in *H. pylori* infection and its association with oxidative stress,” *World Journal of Gastroenterology*, vol. 12, no. 42, pp. 6865–6868, 2006.
      129. M. Aslan, Y. Nazligul, M. Horoz, et al., “Serum prolidase activity and oxidative status in *Helicobacter pylori* infection,” *Clinical Biochemistry*, vol. 40, no. 1-2, pp. 37–40, 2007.
      130. K. Serefhanoglu, A. Taskin, H. Turan, F. E. Timurkaynak, H. Arslan and O. Erel, “Evaluation of oxidative status in patients with brucellosis,” *The Brazilian Journal of Infectious Diseases*, vol. 13, no. 4, pp. 249–251, 2009.
      131. H. Karsen, M. Sunnetcioglu, R. M. Ceylan, et al., “Evaluation of oxidative status in patients with *Fasciola hepatica* infection,” *African Health Sciences*, vol. 11, Suppl. 1, pp. S14–18, 2011.
      132. A. C. Dulger, M. Aslan, Y. Nazligul, et al., “Peripheral lymphocyte DNA damage and oxidative status after eradication therapy in patients infected with *Helicobacter pylori*,” *Polskie Archiwum Medycyny Wewnętrznej*, vol. 121, no. 12, pp. 428–432, 2011.
      133. Y. Nazligul, M. Aslan, M. Horoz, et al., “The effect on serum myeloperoxidase activity and oxidative status of eradication treatment in patients *Helicobacter pylori* infected,” *Clinical Biochemistry*, vol. 44, no. 8-9, pp. 647–649, 2011.
      134. L. Karaagac, S. T. Koruk, I. Koruk and N. Aksoy, “Decreasing oxidative stress in response to treatment in patients with brucellosis: could it be used to monitor treatment?,” *International Journal of Infectious Diseases*, vol. 15, no. 5, pp. e346–349, 2011.
      135. F. Duygu, S. T. Koruk, H. Karsen, N. Aksoy, A. Taskin and M. Hamidanoglu, “Prolidase and oxidative stress in chronic hepatitis C,” *Journal of Clinical Laboratory Analysis*, vol. 26, no. 4, pp. 232–237, 2012.
      136. F. Duygu, H. Karsen, N. Aksoy and A. Taskin, “Relationship of oxidative stress in hepatitis B infection activity with HBV DNA and fibrosis,” *Annals of Laboratory Medicine*, vol. 32, no. 2, pp. 113–118, 2012.
      137. M. Usta, Z. Aras and A. Tas, “Oxidant and antioxidant parameters in patients with *Brucella canis*,” *Clinical Biochemistry*, vol. 45, no. 4-5, pp. 366–367, 2012.
      138. H. Aydin, F. M. Guven, A. Yilmaz, A. Engin, I. Sari and D. Bakir, “Oxidative stress in the adult and pediatric patients with Crimean-Congo haemorrhagic fever,” *Journal of Vector Borne Diseases*, vol. 50, no. 4, pp. 297–301, 2013.
      139. E. Karadag-Oncel, O. Erel, Y. Ozsurekci, et al., “Plasma oxidative stress and total thiol levels in Crimean-Congo hemorrhagic fever,” *Japanese Journal of Infectious Diseases*, vol. 67, no. 1, pp. 22–26, 2014.
      140. R. Esen, M. Aslan, M. E. Kucukoglu, et al., “Serum paraoxonase activity, total thiols levels, and oxidative status in patients with acute brucellosis,” *Wiener Klinische Wochenschrift*, vol. 127, no. 11-12, pp. 427–433, 2015.
      141. C. Nayki, M. Gunay, M. Kulhan, U. Nayki, M. Cankaya and N. G. Kulhan, “Serum levels of soluble interleukin-2 receptor in association with oxidative stress index in patients with different types of HPV,” *Ginekologia Polska*, vol. 88, no. 7, pp. 355–359, 2017.
      142. J. Xianyu, J. Feng, Y. Yang, J. Tang, G. Xie and L. Fan, “Correlation of oxidative stress in patients with HBV-induced liver disease with HBV genotypes and drug resistance mutations,” *Clinical Biochemistry*, vol. 55, pp. 21–27, 2018.
      143. D. Wang, J. F. Feng, P. Zeng, Y. H. Yang, J. Luo and Y. W. Yang, “Total oxidant/antioxidant status in sera of patients with thyroid cancers,” *Endocrine-Related Cancer*, vol. 18, no. 6, pp. 773–782, 2011.
      144. J. F. Feng, L. Lu, P. Zeng, et al., “Serum total oxidant/antioxidant status and trace element levels in breast cancer patients,” *International Journal of Clinical Oncology*, vol. 17, no. 6, pp. 575–583, 2012.
      145. H. Y. Ellidag, E. Eren, O. Aydin, et al., “Ischemia modified albumin levels and oxidative stress in patients with bladder cancer,” Asian Pacific Journal of Cancer Prevention, vol. 14, no. 5, pp. 2759–2763, 2013.
      146. H. Y. Ellidag, N. Bulbuller, E. Eren, et al., “Ischemia-modified albumin: could it be a new oxidative stress biomarker for colorectal carcinoma?,” *Gut Liver*, vol. 7, no. 6, pp. 675–680, 2013.
      147. C. Sezgi, M. Taylan, H. S. Sen, et al., “Oxidative status and acute phase reactants in patients with environmental asbestos exposure and mesothelioma,” *Scientific World Journal*, vol. 2014, pp. 902748, 2014.
      148. J. F. Feng, L. Lu, C. M. Dai, et al., “Analysis of the diagnostic efficiency of serum oxidative stress parameters in patients with breast cancer at various clinical stages,” *Clinical Biochemistry*, vol. 49, no. 9, pp. 692–698, 2016.
      149. Q. Huang, J. Feng, R. Wu, et al., “Total oxidant/antioxidant status in sera of patients with esophageal cancer,” *Medical Science Monitor*, vol. 23, pp. 3789–3794, 2017.
      150. R. Wu, J. Feng, Y. Yang, et al., “Significance of serum total oxidant/antioxidant status in patients with colorectal cancer,” *PLoS One*, vol. 12, no. 1, pp. e0170003, 2017.
      151. G. Batmaz, E. Kιlιç, P. Özcan, E. A. Sarιoğlu, N. Karaca and B. Dane, “The role of oxidative stress in premalignant lesions,” *European Journal od Gynaecological Oncology*, vol. 38, no. 2, pp. 282–285, 2017.
      152. K. Zabłocka-Słowińska, S. Płaczkowska, A. Prescha, et al., “Systemic redox status in lung cancer patients is related to altered glucose metabolism,” *PLoS One*, vol. 13, no. 9, pp. e0204173, 2018.
      153. M. Mehdi, M. K. C. Menon, N. Seyoum, M. Bekele, W. Tigeneh and D. Seifu, “Blood and tissue enzymatic activities of GDH and LDH, index of glutathione, and oxidative stress among breast cancer patients attending referral hospitals of Addis Ababa, Ethiopia: hospital-based comparative cross-sectional study,” *Oxidative Medicine and Cellular Longevity*, vol. 2018, pp. 6039453, 2018.
      154. K. Zabłocka-Słowińska, S. Płaczkowska, A. Prescha, et al., “Serum and whole blood Zn, Cu and Mn profiles and their relation to redox status in lung cancer patients,” *Journal of Trace Elements in Medicine and Biology*, vol. 45, pp. 78–84, 2018.
      155. K. Zabłocka-Słowińska, S. Płaczkowska, K. Skórska, et al., “Oxidative stress in lung cancer patients is associated with altered serum markers of lipid metabolism,” *PLoS One*, vol. 14, no. 4, pp. e0215246, 2019.
      156. K. A. Zabłocka-Słowińska, K. Skórska, S. Placzkowska, et al., “The relationships between glycemic index and glycemic load of diets and nutritional status and antioxidant/oxidant status in the serum of patients with lung cancer,” *Advances in Clinical and Experimental Medicine*, 2019 Mar 9. [Epub ahead of print]
      157. S. Emre, A. Metin, D. D. Demirseren, et al., “The association of oxidative stress and disease activity in seborrheic dermatitis,” *Archives of Dermatological Research*, vol. 304, no. 9, pp. 683–687, 2012.
      158. Y. Yesilova, E. Turan, D. Ucmak, S. Selek, İ. Halil Yavuz and O. Tanrıkulu, “Reduced serum paraoxonase-1 levels in vitiligo: further evidence of oxidative stress,” *Redox Report*, vol. 17, no. 5, pp. 214–218, 2012.
      159. S. Kaur, K. Zilmer, V. Leping and M. Zilmer, “Serum methylglyoxal level and its association with oxidative stress and disease severity in patients with psoriasis,” *Archives of Dermatological Research*, vol. 305, no. 6, pp. 489–494, 2013.
      160. S. Kilic, S. Emre, A. Metin, S. Isikoglu and O. Erel, “Effect of the systemic use of methotrexate on the oxidative stress and paraoxonase enzyme in psoriasis patients,” *Archives of Dermatological Research*, vol. 305, no. 6, pp. 495–500, 2013.
      161. G. Akoglu, S. Emre, A. Metin, et al., “Evaluation of total oxidant and antioxidant status in localized and generalized vitiligo,” *Clinical and Experimental Dermatology*, vol. 38, no. 7, pp. 701–706, 2013.
      162. F. Karababa, Y. Yesilova, E. Turan, S. Selek, H. Altun and S. Selek, “Impact of depressive symptoms on oxidative stress in patients with psoriasis,” *Redox Report*, vol. 18, no. 2, pp. 51–55, 2013.
      163. S. D. Pektas, G. Akoglu, A. Metin, S. Neselioglu and O. Erel, “Evaluation of systemic oxidant/antioxidant status and paraoxonase 1 enzyme activities in psoriatic patients treated by narrow band ultraviolet B phototherapy,” *Redox Report*, vol. 18, no. 5, pp. 200–204, 2013.
      164. S. Emre, A. Metin, D. D. Demirseren, S. Kilic, S. Isikoglu and O. Erel, “The relationship between oxidative stress, smoking and the clinical severity of psoriasis,” *Journal of the European Academy of Dermatology and Venereology*, vol. 27, no. 3, pp. e370–375, 2013.
      165. Y. Yesilova, D. Ucmak, S. Selek, et al., “Oxidative stress index may play a key role in patients with pemphigus vulgaris,” *Journal of the European Academy of Dermatology and Venereology*, vol. 27, no. 4, pp. 465–467, 2013.
      166. S. G. Bilgili, H. Ozkol, A. S. Karadag, et al., “Serum paraoxonase activity and oxidative status in subjects with alopecia areata,” *Cutaneous and Ocular Toxicology*, vol. 32, no. 4, pp. 290–293, 2013.
      167. O. A. Bakry, R. M. Elshazly, M. A. Shoeib and A. Gooda, “Oxidative stress in alopecia areata: a case-control study,” *American Journal of Clinical Dermatology*, vol. 15, no. 1, pp. 57–64, 2014.
      168. M. Rajappa, R. Shanmugam, M. Munisamy, L. Chandrashekar, K. S. Rajendiran and D. M. Thappa, “Effect of antipsoriatic therapy on oxidative stress index and sialic acid levels in patients with psoriasis,” *International Journal of Dermatology*, vol. 55, no. 8, pp. e422– 430, 2016.
      169. S. Emre, G. Akoglu, A. Metin, et al., “The oxidant and antioxidant status in pityriasis rosea,” *Indian Journal of Dermatology*, vol. 61, no. 1, pp. 118, 2016.

# H. Kaya Erdogan, I. Bulur, E. Kocaturk, B. Yildiz, Z. N. Saracoglu and O. Alatas, “The role of oxidative stress in early-onset androgenetic alopecia,” *Journal of Cosmetic Dermatology*, vol. 16, no. 4, pp. 527–530, 2017.

- - - 1. M. I. Kara, S. Yanık, A. Keskinruzgar, et al., “Oxidative imbalance and anxiety in patients with sleep bruxism,” *Oral Surgery, Oral Medicine, Oral Pathology and Oral Radiology*, vol. 114, no. 6, pp. 604–609, 2012.
      2. S. G. Bilgili, H. Ozkol, Z. Takci, H. U. Ozkol, A. S. Karadag and M. Aslan, “Assessment of the serum paraoxonase activity and oxidant/antioxidant status in patients with recurrent aphthous stomatitis,” *International Journal of Dermatology*,2013; 52(10): 1259-64.
      3. U. Sezer, K. Erciyas, K. Ustün, et al., “Effect of chronic periodontitis on oxidative status in patients with rheumatoid arthritis,” *Journal of Periodontology*, vol. 84, no. 6, pp. 785–792, 2013.
      4. E. Avci, Z. Z. Akarslan, Erten H, Coskun-Cevher S. Oxidative stress and cellular immunity in patients with recurrent aphthous ulcers,” *Brazilian Journal of Medical and Biological Research*, vol. 47, no. 5, pp. 355–360, 2014.
      5. U. Sezer, S. Z. Şenyurt, H. Gündoğar, et al., “Effect of chronic periodontitis on oxidative status in patients with psoriasis and psoriatic arthritis,” *Journal of Periodontology*, vol. 87, no. 5, pp. 557–565, 2016.
      6. S. Tugrul, A. Koçyiğit, R. Doğan, et al., “Total antioxidant status and oxidative stress in recurrent aphthous stomatitis,” *International Journal of Dermatology*, vol. 55, no. 3, pp. e130–135, 2016.
      7. H. S. Kavakli, O. Erel, O. Delice, G. Gormez, S. Isikoglu and F. Tanriverdi, “Oxidative stress increases in carbon monoxide poisoning patients,” *Human and Experimental Toxicology*, vpñ. 30, no. 2, pp. 160–164, 2011.
      8. T. Aran, M. A. Unsal, S. Guven, C. Kart, E. C. Cetin and A. Alver, “Carbon dioxide pneumoperitoneum induces systemic oxidative stress: a clinical study,” *European Journal of Obstetrics & Gynecology and Reproductive Biology*, vol. 161, no. 1, pp. 80–83, 2012.
      9. E. Baysal, S. Taysi, N. Aksoy, et al., “Serum paraoxonase, arylesterase activity and oxidative status in patients with obstructive sleep apnea syndrome (OSAS),” *European Review for Medical and Pharmacological Sciences*, vol. 16, no. 6, pp. 770–774, 2012.
      10. N. Hilali, M. Vural, H. Camuzcuoglu, A. Camuzcuoglu and N. Aksoy, “Increased prolidase activity and oxidative stress in PCOS,” *Clinical Endocrinology*, vol. 79, no. 1, pp. 105–110, 2013.
      11. B. Halici, S. Sarinc Ulasli, E. Günay, et al., “Assessment of inflammatory biomarkers and oxidative stress in pulmonary thromboembolism: follow-up results,” *Inflammation*, vol. 37, no. 4, pp. 1186–1190, 2014.
      12. R. Aslan, R. Kutlu, S. Civi and E. Tasyurek, “The correlation of the total antioxidant status (TAS), total oxidant status (TOS) and paraoxonase activity (PON1) with smoking,” *Clinical Biochemistry*, vol. 47, no. 6, pp. 393–397, 2014.
      13. E. Ugurlu, E. Kilic-Toprak, G. Altinisik, et al., “Increased erythrocyte aggregation and oxidative stress in patients with idiopathic interstitial pneumonia,” *Sarcoidosis Vasculitis and Diffuse Lung Diseases*, vol. 33, no. 4, pp. 308–316, 2016.
      14. A. Arısoy, S. Ekin, B. Sertogullarindan, et al., “The relationship among oxidative and anti-oxidative parameters and myeloperoxidase in subjects with obstructive sleep apnea syndrome,” *Respiratory Care*, vol. 61, no. 2, pp. 200–204, 2016.
      15. F. K. Yalcin, M. Er, H. C. Hasanoglu, et al., “Deteriorations of pulmonary function, elevated carbon monoxide levels and increased oxidative stress amongst water-pipe smokers,” *International Journal of Occupational Medicine and Environmental Health, vol.* 30, no. 5, pp. 731–742, 2017.
      16. S. Ekin, A. Arısoy, H. Gunbatar, et al., “The relationships among the levels of oxidative and antioxidative parameters, FEV1 and prolidase activity in COPD,” *Redox Report*, vol. 22, no. 2, pp. 74–77, 2017.
      17. M. Karademirci, R. Kutlu and I. Kilinc, “Relationship between smoking and total antioxidant status, total oxidant status, oxidative stress index, vit C, vit E,” *The Clinical Respiratory Journal*, vol. 12, no. 6, pp. 2006–2012, 2018.
      18. N. Dizen-Namdar, F. Emel Kocak, M. Kidir, G. Sarici, H. Tak and I. Altuntas, “Evaluation of serum paraoxonase, arylesterase, prolidase activities and oxidative stress in patients with alopecia areata,” *Skin Pharmacology and Physiology*, vol. 32, no. 2, pp. 59–64, 2019.
      19. R. Demirbag, R. Yilmaz, M. Gur, et al., “DNA damage in metabolic syndrome and its association with antioxidative and oxidative measurements,” *International Journal of Clinical Practice*, vol. 60, no. 10, pp. 1187–1193, 2006.
      20. M. Aslan, T. Sabuncu, A. Kocyigit, H. Celik and S. Selek, “Relationship between total oxidant status and severity of diabetic nephropathy in type 2 diabetic patients,” *Nutrition, Metabolism & Cardiovascular Diseases*, vol. 17, no. 10, pp. 734–740, 2007.
      21. A. Kaya, I. Uzunhasan, M. Baskurt, et al., “Oxidative status and lipid profile in metabolic syndrome: gender differences,” *Metabolic Syndrome and Related Disorders*, vol. 8, no. 1, pp. 53–58, 2010.
      22. Z. Bahadoran, P. Mirmiran, F. Hosseinpanah, M. Hedayati, S. Hosseinpour-Niazi and F. Azizi. “Broccoli sprouts reduce oxidative stress in type 2 diabetes: a randomized double-blind clinical trial,” *European Journal of Clinical Nutrition*, vol. 65, no. 8, pp. 972–977, 2011.
      23. A. N. Torun, M. Vural, H. Cece, H. Camuzcuoglu, H. Toy and N. Aksoy, “Paraoxonase-1 is not affected in polycystic ovary syndrome without metabolic syndrome and insulin resistance, but oxidative stress is altered,” Gynecological Endocrinology, vol.27, no.12, pp. 988–992, 2011.
      24. E. Uzar, Y. Tamam, O. Evliyaoglu, et al., “Serum prolidase activity and oxidative status in patients with diabetic neuropathy.” *Neurological Sciences*, vol. 33, no. 4, pp. 875–880, 2012.
      25. S. Tabur, H. Korkmaz, M. A. Eren, E. Oğuz, T. Sabuncu and N. Aksoy, “Urotensin-II level and its association with oxidative stress in early diabetic nephropathy,” *Journal of Diabetes and Its Complications*, vol. 29, no. 1, pp. 115–119, 2015.
      26. I. Ates, N. Ozkayar, C. Topcuoglu and F. Dede, “Relationship between oxidative stress parameters and asymptomatic organ damage in hypertensive patients without diabetes mellitus,” *Scandinavian Cardiovascular Journal*, vol. 49, no. 5, pp. 249–256, 2015.

# M. Aslan, U. Duzenli, R. Esen and Y. U. Soyoral, “Serum prolidase enzyme activity in obese subjects and its relationship with oxidative stress markers,” *Clinica Chimica Acta*, vol. 473, pp. 186–190, 2017.

- - - 1. C. A. Aral, Ö. Nalbantoğlu, B. G. Nur, M. Altunsoy and K. Aral, “Metabolic control and periodontal treatment decreases elevated oxidative stress in the early phases of type 1 diabetes onset,” *Archives of Oral Biology*, vol. 82, pp. 115–120, 2017.
      2. R. Demirbağ, R. Yilmaz, S. Güzel, H. Celik, A. Koçyigit and E. Ozcan, “Effects of treadmill exercise test on oxidative/antioxidative parameters and DNA damage,” *Anadolu Kardiyoloji Dergisi*, vol. 6, no. 2, pp. 135–140, 2006.
      3. O. Altindag, O. Erel, N. Aksoy, S. Selek, H. Celik and M. Karaoglanoglu, “Increased oxidative stress and its relation with collagen metabolism in knee osteoarthritis,” *Rheumatology International*, vol. 27, no. 4, pp. 339–344, 2007.
      4. S. Selek, M. Aslan, M. Horoz, M. Gur and O. Erel, “Oxidative status and serum PON1 activity in beta-thalassemia minor,” *Clinical Biochemistry*, vol. 40, no. 5-6, pp. 287–291, 2007.
      5. M. Karakoc, O. Altindag, H. Keles, N. Soran and S. Selek, “Serum oxidative-antioxidative status in patients with ankylosing spondylitis,” *Rheumatology International*, vol. 27, no. 12, pp. 1131–1134, 2007.
      6. A. Isik, S. S. Koca, B. Ustundag and S. Selek, “Decreased total antioxidant response and increased oxidative stress in Behcet's disease,” *The Tohoku Journal of Experimental Medicine*, vol. 212, no. 2, pp. 133–141, 2007.
      7. O. Sirmatel, C. Sert, F. Sirmatel, S. Selek and B. Yokus, “Total antioxidant capacity, total oxidant status and oxidative stress index in the men exposed to 1.5 T static magnetic field,” *General Physiology and Biophysics*, vol. 26, no. 2, pp. 86–90, 2007.
      8. O. Altindag, O. Erel, N. Soran, H. Celik and S. Selek, “Total oxidative/anti-oxidative status and relation to bone mineral density in osteoporosis,” *Rheumatology International*, vol. 28, no. 4, pp. 317–321, 2008.
      9. A. Nagila, T. Permpongpaiboon, S. Tantrarongroj, et al., “Effect of atorvastatin on paraoxonase1 (PON1) and oxidative status,” *Pharmacological Reports*, vol. 61, no. 5, pp. 892–898, 2009.
      10. Z. Baysal, M. Cengiz, A. Ozgonul, M. Cakir, H. Celik and A. Kocyigit, “Oxidative status and DNA damage in operating room personnel,” *Clinical Biochemistry*, vol. 42, no. 3, pp. 189–193, 2009.
      11. M. Horoz, M. Aslan, A. O. Koylu, et al., “The relationship between leptin level and oxidative status parameters in hemodialysis patients,” *Artificial Organs*, vol. 33, no. 1, pp. 81–85, 2009.
      12. R. Alp, S. Selek, S. I. Alp, A. Taşkin and A. Koçyiğit, “Oxidative and antioxidative balance in patients of migraine,” *European Review for Medical and Pharmacological Sciences*, vol. 14, no. 10, pp. 877–882, 2010.
      13. A. Cakmak, M. Soker, A. Koc and N. Aksoy, “Prolidase activity and oxidative status in patients with thalassemia major,” *Journal of Clinical Laboratory Analysis*, vol. 24, no. 1, pp. 6–11, 2010.
      14. S. Addisu, T. H. El-Metwally, G. Davey, Y. Worku and M. A. Titheradge, “The role of transforming growth factor-beta1 and oxidative stress in podoconiosis pathogenesis,” *British Journal of Dermatology*, vol. 162, no. 5, pp. 998–1003, 2010.
      15. H. Buyukhatipoglu, I. Kirhan, M. Vural, et al., “Oxidative stress increased in healthcare workers working 24-hour on-call shifts,” *The American Journal of the Medical Sciences*, vol. 340, no. 6, pp. 462–467, 2010.
      16. E. Cebeci, F. A. Oner, M. Usta, S. Yurdakul and M. Erguney, “Evaluation of oxidative stress, the activities of paraoxonase and arylesterase in patients with subclinic hypothyroidism,” *Acta Bio-medica*, vol. 82, no. 3, pp. 214–222, 2011.
      17. N. Yilmaz, O. Aydin, A. Yegin, A. Tiltak, E. Eren and G. Aykal, “Impaired oxidative balance and association of blood glucose, insulin and HOMA-IR index in migraine,” *Biochemia Medica*, vol. 21, no. 2, pp. 145–151, 2011.
      18. S. Koc, N. Aksoy, H. Bilinc, F. Duygu, I. Ö. Uysal and A. Ekinci, “Paraoxonase and arylesterase activity and total oxidative/anti-oxidative status in patients with chronic adenotonsillitis,” *International Journal of Pediatric Otorhinolaryngology*, vol. 75, no. 11, pp. 1364–1367, 2011.
      19. M. Aslan, Y. Nazligu, C. Bolukbas, et al., “Peripheral lymphocyte DNA damage and oxidative stress in patients with ulcerative colitis,” *Polskie Archiwum Medycyny Wewnętrznej*, vol. 127, no. 7-8, pp. 223–229, 2011.
      20. N. Yilmaz, O. Aydin, A. Yegin, A. Tiltak and E. Eren, “Increased levels of total oxidant status and decreased activity of arylesterase in migraineurs,” *Clinical Biochemistry*, vol.44, no. 10-11, pp. 832–837, 2011.
      21. M. Aslan, N. Cosar, H. Celik, et al., “Evaluation of oxidative status in patients with hyperthyroidism,” *Endocrine*, vol.40, no. 2, pp. 285–289, 2011.
      22. M. Horoz, A. A. Kiykim, B. Cimen and A. Erdem, “The influence of hemodialysis membrane permeability on serum paraoxonase-1 activity and oxidative status parameters,” *Artificial Organs*, vol. 35, no. 10, pp. 923–929, 2011.
      23. M. A. Altay, C. Erturk, N. Aksoy, et al., “Serum prolidase activity and oxidative-antioxidative status in Legg-Calve-Perthes disease,” *Journal of Pediatric Orthopaedics B*, vol. 20, no. 4, pp. 222–226, 2011.
      24. M. A. Cikrikcioglu, M. Hursitoglu, H. Erkal, et al., “Oxidative stress and autonomic nervous system functions in restless legs syndrome,” *European Journal of Clinical Investigation*, vol. 41, no. 7, pp. 734–742, 2011.
      25. T. Permpongpaiboon, A. Nagila, P. Pidetcha, K. Tuangmungsakulchai, S. Tantrarongroj and S. Porntadavity, “Decreased paraoxonase 1 activity and increased oxidative stress in low lead-exposed workers,” *Human and Experimental Toxicology*, vol. 30, no. 9, pp. 1196–1203, 2011.
      26. A. Demirkol, M. Uludag, N. Soran, et al., “Total oxidative stress and antioxidant status in patients with carpal tunnel syndrome,” *Redox Report*, vol. 17, no. 6, pp. 234–238, 2012.
      27. A. Acar, M. Ugur Cevik, O. Evliyaoglu, et al., “Evaluation of serum oxidant/antioxidant balance in multiple sclerosis,” *Acta Neurologica Belgica*, vol. 112, no. 3, pp. 275–280, 2012.
      28. M. Aldemir, E. Okulu, S. Neşelioğlu, O. Erel, K. Ener and Ö. Kayıgil, “Evaluation of serum oxidative and antioxidative status in patients with erectile dysfunction,” *Andrologia*, vol. 44, suppl. 1, pp. 266–271, 2012.
      29. M. T. Gokdemir, O. Sogut, H. Kaya, et al., “Role of oxidative stress in the clinical outcome of patients with multiple blunt trauma,” *The Journal of International Medical Research*, vol. 40, no. 1, pp. 167–173, 2012.
      30. C. Ertürk, M. A. Altay, S. Selek and A. Koçyiğit, “Paraoxonase-1 activity and oxidative status in patients with knee osteoarthritis and their relationship with radiological and clinical parameters,” *Scandinavian Journal of Clinical and Laboratory Investigation*, vol. 72, no. 5, pp. 433–439, 2012.
      31. C. Ertürk, M. A. Altay, S. Selek and A. Koçyiğit, “Paraoxonase-1 activity and oxidative status in patients with knee osteoarthritis and their relationship with radiological and clinical parameters,” *Scandinavian Journal of Clinical and Laboratory Investigation*, vol. 72, no. 5, pp. 433–439, 2012.
      32. A. Turgut, A. Özler, N. Y. Görük, S. Y. Tunc, O. Evliyaoglu and T. Gül, “Copper, ceruloplasmin and oxidative stress in patients with advanced-stage endometriosis,” *European Review for Medical and Pharmacological Sciences*, vol. 17, no. 11, pp. 1472–1478, 2013.
      33. M. Abuhandan, M. Calik, A. Taskin, I. Yetkin, S. Selek and A. Iscan, “The oxidative and antioxidative status of simple febrile seizure patients,” *The Journal of the Pakistan Medical Association*, vol. 63, no. 5, pp. 594–597, 2013.
      34. A. Kirbas, S. Kirbas, O. Anlar, H. Efe and A. Yilmaz, “Serum paraoxonase and arylesterase activity and oxidative status in patients with multiple sclerosis,” *Journal of Clinical Neuroscience*, vol. 20, no. 8, pp. 1106–1109, 2013.
      35. H. Kayabasi, Z. Yilmaz, D. Sit, A. K. Kadiroglu and E. Yilmaz, “The effects of losartan on oxidative stress and inflammation in non-diabetic patients undergoing chronic hemodialysis,” *European Review for Medical and Pharmacological Sciences*, vol. 17, no. 2, pp. 235–242, 2013.
      36. M. Atli, M. Aslan, M. Emin Kucukoglu, H. B. Temur, A. Taskin and H. Celik, “Peripheral lymphocyte DNA damage and oxidative status in football players after a three-day football tournament,” *Internal Medicine*, vol. 52, no. 2, pp. 213–217, 2013.
      37. W. Hao, Y. Zhu, L. Meng, C. Ni, J. Yang and H. Zhou, “Serum paraoxonase, arylesterase activity, and oxidative status in patients with nasal polyp,” *European Archives of Oto-rhino-laryngology*, vol. 270, no. 6, pp. 1861–1865, 2013.
      38. M. Neyal, F. Yimenicioglu, A. Aydeniz, et al., “Plasma nitrite levels, total antioxidant status, total oxidant status, and oxidative stress index in patients with tension-type headache and fibromyalgia,” *Clinical Neurology and Neurosurgery*, vol. 115, no. 6, pp. 736–740, 2013.
      39. E. Varol, A. Icli, F. Aksoy, et al., “Evaluation of total oxidative status and total antioxidant capacity in patients with endemic fluorosis,” *Toxicology and Industrial Health*, vol. 29, no. 2, pp. 175–180, 2013.
      40. B. Liang, Y. H. Li and H. Kong, “Serum paraoxonase, arylesterase activities and oxidative status in patients with insomnia,” *European Review for Medical and Pharmacological Sciences*, vol. 17, no. 18, pp. 2517–2522, 2013.
      41. A. Durmus, A. Mentese, M. Yilmaz, et al., “Increased oxidative stress in patients with essential thrombocythemia,” *European Review for Medical and Pharmacological Sciences*, vol. 17, no. 21, pp. 2860–2866, 2013.
      42. F. Bozkus, I. San, T. Ulas, et al., “Evaluation of total oxidative stress parameters in patients with nasal polyps,” *Acta Otorhinolaryngologica Italica*, vol. 33, no. 4, pp. 248–253, 2013.
      43. T. Kaldur, J. Kals, V. Ööpik, et al., “Effects of heat acclimation on changes in oxidative stress and inflammation caused by endurance capacity test in the heat,” *Oxidative Medicine and Cellular Longevity*, vol. 2014, pp. 107137, 2014.
      44. K. A. Turkdogan, O. Akpinar, M. Karabacak, H. Akpinar, F. T. Turkdogan and O. Karahan, “Association between oxidative stress index and serum lipid levels in healthy young adults,” *The Journal of the Pakistan Medical Association*, vol. 64, no. 4, pp. 379–381, 2014.
      45. E. Savas, N. Aksoy, Y. Pehlivan, et al., “Evaluation of oxidant and antioxidant status and relation with prolidase in systemic sclerosis,” *Wiener Klinische Wochenschrift*, vol. 126, no. 11-12, pp. 341–346, 2014.
      46. A. Durmus, A. Mentese, M. Yilmaz, et al., “The thrombotic events in polycythemia vera patients may be related to increased oxidative stress,” *Medical Principles and Practice*, vol. 23, no. 3, pp. 253–258, 2014.
      47. M. Cevik, P. Yazgan and N. Aksoy, “Evaluation of antioxidative/oxidative status and prolidase parameters in cases of inguinal hernia with joint hypermobility syndrome,” *Hernia*, vol. 18, no. 6, pp. 849–853, 2014.
      48. I. Toprak, V. Kucukatay, C. Yildirim, E. Kilic-Toprak and O. Kilic-Erkek, “Increased systemic oxidative stress in patients with keratoconus,” *Eye*, vol. 28, no. 3, pp. 285–289, 2014.
      49. M. Bozkurt, M. Caglayan, P. Oktayoglu, et al., “Serum prolidase enzyme activity and oxidative status in patients with fibromyalgia,” *Redox Report*, vol. 19, no. 4, pp. 148–153, 2014.
      50. M. Bozkurt, H. Yüksel, S. Em, et al., “Serum prolidase enzyme activity and oxidative status in patients with Behçet's disease,” *Redox Report*, vol. 19, no. 2, pp. 59–64, 2014.
      51. M. Karabacak, E. Varol, F. Kahraman, M. Ozaydin, A. K. Türkdogan and I. H. Ersoy, “Low high-density lipoprotein cholesterol is characterized by elevated oxidative stress,” *Angiology*, vol. 65, no. 10, pp. 927–931, 2014.
      52. S. Zengin, B. Al, P. Yarbil, et al., “An assessment of oxidant/antioxidant status in patients with snake envenomation,” *Emergency Medicine Journal*, vol. 31, no. 1, pp. 48–52, 2014.
      53. T. Göncü, A. Akal, F. M. Adıbelli, S. Çakmak, H. Sezen and Ö. F. Yılmaz, “Tear film and serum prolidase activity and oxidative stress in patients with keratoconus,” *Cornea*, vol. 34, no. 9, pp. 1019–1023, 2015.
      54. M. A. Altay, C. Ertürk, A. Bilge, M. Yaptı, A. Levent and N. Aksoy, “Evaluation of prolidase activity and oxidative status in patients with knee osteoarthritis: relationships with radiographic severity and clinical parameters,” *Rheumatology International*, vol. 35, no. 10, pp. 1725–1731, 2015.

# S. Tuğrul, R. Doğan, A. Kocyigit, E. Torun, E. Senturk and O. Ozturan, “DNA damage and oxidative status in PFAPA syndrome,” *Auris Nasus Larynx*, vol. 42, no. 5, pp. 406–411, 2015.

- - - 1. Y. Eren, E. Dirik, S. Neşelioğlu and Ö. Erel, “Oxidative stress and decreased thiol level in patients with migraine: cross-sectional study,” *Acta Neurologica Belgica*, vol. 115, no. 4, pp. 643–649, 2015.
      2. N. Aktepe, A. Kocyigit, Y. Yukselten, A. Taskin, C. Keskin and H. Celik, “Increased DNA damage and oxidative stress among silver jewelry workers,” *Biological Trace Element Research*, vol. 164, no. 2, pp. 185–191, 2015.
      3. I. Gecit, I. Meral, M. Aslan, et al., “Peripheral mononuclear leukocyte DNA damage, plasma prolidase activity, and oxidative status in patients with benign prostatic hyperplasia,” *Redox Report*, vol. 20, no. 4, pp. 163–169, 2015.
      4. U. Kilic, O. Gok, U. Erenberk, et al., “A remarkable age-related increase in SIRT1 protein expression against oxidative stress in elderly: SIRT1 gene variants and longevity in human,” *PLoS One*, vol. 10, no. 3, pp. e0117954, 2015.
      5. H. Uçar, M. Gür, M. Y. Gözükara, et al., “Gamma glutamyl transferase activity is independently associated with oxidative stress rather than SYNTAX score,” *Scandinavian Journal of Clinical and Laboratory Investigation*, vol. 75, no. 1, pp. 7–12, 2015.
      6. İ. Atik, N. Kozacı, İ. Beydilli, M. Avcı, H. Ellidağ and M. Keşaplı, “Investigation of oxidant and antioxidant levels in patients with acute stroke in the emergency service,” *American Journal of Emergency Medicine*, vol. 34, no. 12, pp. 2379–2383, 2016.
      7. I. Ates, M. Altay, F. M. Yilmaz, et al., “The impact of levothyroxine sodium treatment on oxidative stress in Hashimoto's thyroiditis,” *European Journal of Endocrinology*, vol. 174, no. 6, pp. 727–734, 2016.
      8. R. Altintas, C. Ediz, H. Celik, et al., “The effect of varicocoelectomy on the relationship of oxidative stress in peripheral and internal spermatic vein with semen parameters,” *Andrology*, vol. 4, no. 3, pp. 442–446, 2016.
      9. S. Geyik, E. Altunısık, A. M. Neyal and S. Taysi, “Oxidative stress and DNA damage in patients with migraine,” *The Journal of Headache and Pain*, vol. 17, pp. 10, 2016.
      10. A. Dur, O. Kocaman, A. Koçyiğit, et al., “Oxidative status and lymphocyte DNA damage in patients with acute pancreatitis and its relationship with severity of acute pancreatitis,” *The Turkish Journal of Gastroenterology*, vol. 27, no. 1, pp. 68–72, 2016.
      11. M. N. Turan, M. Aslan, F. F. Bolukbas, C. Bolukbas, S. Selek snd T. Sabuncu, “The effect of upper gastrointestinal system endoscopy process on serum oxidative stress levels,” *Wiener Klinische Wochenschrift*, vol.128, suppl. 8, pp. 572–575, 2016.
      12. F. Davran, V. T. Yilmaz, B. K. Erdem, M. Gultekin, G. Suleymanlar and H. Akbas, “Association of interleukin 18-607A/C and -137C/G polymorphisms with oxidative stress in renal transplant recipients,” *Renal Failure*, vol. 38, no. 5, pp. 717–722, 2016.

# B. Pietrucha, E. Heropolitanska-Pliszka, M. Maciejczyk, et al., “Comparison of selected parameters of redox homeostasis in patients with ataxia-telangiectasia and Nijmegen Breakage Syndrome,” *Oxidative Medicine and Cellular Longevity*, vol. 2017, pp. 6745840, 2017.

- - - 1. R. Zhang, Q. Song, H. Liu, et al., “Effect of the R92H and A379V genotypes of platelet-activating factor acetylhydrolase on its enzyme activity, oxidative stress and metabolic profile in Chinese women with polycystic ovary syndrome,” *Lipids in Health and Disease*, vol. 16, no. 1, pp. 57, 2017.
      2. F. Gul, T. Muderris, G. Yalciner, et al., “A comprehensive study of oxidative stress in sudden hearing loss,” *European Archieves of Oto-rhino-laryngology*, vol. 274, no. 3, pp. 1301–1308, 2017.
      3. R. Zhang, H. Liu, H. Bai, et al., “Oxidative stress status in Chinese women with different clinical phenotypes of polycystic ovary syndrome,” *Clinical Endocrinology*, vol. 86, no. 1, pp. 88–96, 2017.
      4. M. A. Altay, C. Ertürk, A. Levent, B. V. Çetin and N. Aksoy, “Serum prolidase activity and oxidative-antioxidative status in patients with developmental dysplasia of the hip and its relationship with radiographic severity,” *Redox Report*, vol. 22, no. 5, pp. 227–234, 2017.
      5. E. Baysal, S. Gulsen, I. Aytac, et al., “Oxidative stress in otosclerosis,” *Redox Report*, vol. 22, no. 5, pp. 235–239, 2017.
      6. M. Więcek, M. Maciejczyk, J. Szymura, S. Wiecha, M. Kantorowicz and Z. Szygula, “Effect of body composition, aerobic performance and physical activity on exercise-induced oxidative stress in healthy subjects,” *The Journal of Sports Medicine and Physical Fitness*, vol. 57, no. 7-8, pp. 942–952, 2017.
      7. E. Kılıç and M. Uğur, “Effect of therapeutic hypothermia on superficial surgical site infection and postoperative pain in urgent abdominal surgery,” *Ulusal Travma ve Acil Cerrahi Dergisi*, vol. 24, no. 5, pp. 417–422, 2018.
      8. S. H. Eren, I. Korkmaz, F. M. K. Guven, Y. K. Tekin and L. Ozdemir, “Serum paraoxonase, arylesterase, and glutathione-S-transferase activities and oxidative stress levels in patients with mushroom poisoning,” *Clinics*, vol. 73, pp. e16550, 2018.
      9. A. F. Cӑtoi, A. E. Pârvu, A. Mironiuc, et al., “Chemerin, inflammatory, and nitrooxidative stress marker changes six months after sleeve gastrectomy,” *Oxidative Medicine and Cellular Longevity*, vol. 201, pp. 1583212, 2018.
      10. A. Stanek, A. Cholewka, T. Wielkoszyński, E. Romuk and A. Sieroń, “Decreased oxidative stress in male patients with active phase ankylosing spondylitis who underwent whole-body cryotherapy in closed cryochamber,” *Oxidative Medicine and Cellular Longevity*, vol. 2018, pp. 7365490, 2018.
      11. B. Kumru, D. S. Kaplan, B. Oztürk Hismi and H. Celik, “Effect of blood phenylalanine levels on oxidative stress in classical phenylketonuric patients,” *Cellular and Molecular Neurobiology*, vol. 38, no. 5, pp. 1033–1038, 2018.
      12. R. Kocabaş, E. S. Namiduru, A. M. Bagçeci, et al., “The acute effects of interval exercise on oxidative stress and antioxidant status in volleyball players,” *The Journal of Sports Medicine and Physical Fitness*, vol. 58, no. 4, pp. 421–427, 2018.
      13. I. Ates, M. F. Arikan, M. Altay, et al., “The effect of oxidative stress on the progression of Hashimoto's thyroiditis,” *Archives of Physiology and Biochemistry*, vol. 124, no. 4, pp. 351–356, 2018.
      14. İ. Akar, İ. İnce, C. Aslan, et al., “Oxidative stress and prolidase enzyme activity in the pathogenesis of primary varicose veins,” *Vascular*, vol. 26, no. 3, pp. 315–321, 2018.
      15. F. Beyazit, H. Türkön, E. Pek, F. H. Ozturk and M. Ünsal, “Elevated circulating nitric oxide levels correlates with enhanced oxidative stress in patients with hyperemesis gravidarum,” *Journal of Obstetrics and Gynaecology*, vol. 38, no. 5, pp. 668–673, 2018.
      16. A. Ekinci and K. Kamasak, “Evaluation of serum prolidase enzyme activity and oxidative stress in patients with tinnitus,” *Braziliean Journal of Otorhinolaryngology*, 2019 Mar 15. pii: S1808-8694(18)30832-2. [Epub ahead of print]
      17. L. Albayrak, O. Sogut, S. Çakmak, M. T. Gökdemir and H. Kaya, “Plasma oxidative-stress parameters and prolidase activity in patients with various causes of abdominal pain,” *American Journal of Emergency Medicine*, 2019 Apr 16. pii: S0735-6757(19)30252-9. [Epub ahead of print]
